# High-quality assembly of the T2T genome for *Isodon rubescens* f. *lushanensis* reveals genomic structure variations between 2 typical forms of *Isodon rubescens*

**DOI:** 10.1093/gigascience/giae075

**Published:** 2024-10-10

**Authors:** Hao Yang, Conglong Lian, Jinlu Liu, Hongwei Yu, Le Zhao, Ni He, Xiuyu Liu, Shujuan Xue, Xiaoya Sun, Liping Zhang, Lili Wang, Jingfan Yang, Yu Fu, Rui Ma, Bao Zhang, Lidan Ye, Suiqing Chen

**Affiliations:** College of Pharmacy, Henan Key Laboratory of Chinese Medicine Resources and Chemistry, Henan University of Chinese Medicine, Zhengzhou 450046, PR China; Collaborative Innovation Center of Research and Development on the Whole Industry Chain of Yu-Yao, Zhengzhou 450046, PR China; Co-Construction Collaborative Innovation Centre for Chinese Medicine and Respiratory Diseases by Henan & Education Ministry of China, Zhengzhou 450046, PR China; College of Pharmacy, Henan Key Laboratory of Chinese Medicine Resources and Chemistry, Henan University of Chinese Medicine, Zhengzhou 450046, PR China; Collaborative Innovation Center of Research and Development on the Whole Industry Chain of Yu-Yao, Zhengzhou 450046, PR China; Co-Construction Collaborative Innovation Centre for Chinese Medicine and Respiratory Diseases by Henan & Education Ministry of China, Zhengzhou 450046, PR China; College of Pharmacy, Henan Key Laboratory of Chinese Medicine Resources and Chemistry, Henan University of Chinese Medicine, Zhengzhou 450046, PR China; Institute of Bioengineering, College of Chemical and Biological Engineering, Zhejiang University, Hangzhou 310058, PR China; College of Pharmacy, Henan Key Laboratory of Chinese Medicine Resources and Chemistry, Henan University of Chinese Medicine, Zhengzhou 450046, PR China; Institute of Bioengineering, College of Chemical and Biological Engineering, Zhejiang University, Hangzhou 310058, PR China; College of Pharmacy, Henan Key Laboratory of Chinese Medicine Resources and Chemistry, Henan University of Chinese Medicine, Zhengzhou 450046, PR China; College of Pharmacy, Henan Key Laboratory of Chinese Medicine Resources and Chemistry, Henan University of Chinese Medicine, Zhengzhou 450046, PR China; Collaborative Innovation Center of Research and Development on the Whole Industry Chain of Yu-Yao, Zhengzhou 450046, PR China; Co-Construction Collaborative Innovation Centre for Chinese Medicine and Respiratory Diseases by Henan & Education Ministry of China, Zhengzhou 450046, PR China; College of Pharmacy, Henan Key Laboratory of Chinese Medicine Resources and Chemistry, Henan University of Chinese Medicine, Zhengzhou 450046, PR China; Collaborative Innovation Center of Research and Development on the Whole Industry Chain of Yu-Yao, Zhengzhou 450046, PR China; Co-Construction Collaborative Innovation Centre for Chinese Medicine and Respiratory Diseases by Henan & Education Ministry of China, Zhengzhou 450046, PR China; College of Pharmacy, Henan Key Laboratory of Chinese Medicine Resources and Chemistry, Henan University of Chinese Medicine, Zhengzhou 450046, PR China; College of Pharmacy, Henan Key Laboratory of Chinese Medicine Resources and Chemistry, Henan University of Chinese Medicine, Zhengzhou 450046, PR China; College of Pharmacy, Henan Key Laboratory of Chinese Medicine Resources and Chemistry, Henan University of Chinese Medicine, Zhengzhou 450046, PR China; College of Pharmacy, Henan Key Laboratory of Chinese Medicine Resources and Chemistry, Henan University of Chinese Medicine, Zhengzhou 450046, PR China; College of Pharmacy, Henan Key Laboratory of Chinese Medicine Resources and Chemistry, Henan University of Chinese Medicine, Zhengzhou 450046, PR China; College of Pharmacy, Henan Key Laboratory of Chinese Medicine Resources and Chemistry, Henan University of Chinese Medicine, Zhengzhou 450046, PR China; Institute of Bioengineering, College of Chemical and Biological Engineering, Zhejiang University, Hangzhou 310058, PR China; College of Pharmacy, Henan Key Laboratory of Chinese Medicine Resources and Chemistry, Henan University of Chinese Medicine, Zhengzhou 450046, PR China; Collaborative Innovation Center of Research and Development on the Whole Industry Chain of Yu-Yao, Zhengzhou 450046, PR China; Co-Construction Collaborative Innovation Centre for Chinese Medicine and Respiratory Diseases by Henan & Education Ministry of China, Zhengzhou 450046, PR China

**Keywords:** telomere-to-telomere genome sequencing, *I. rubescens* f. *lushanensis*, plant diterpenoid biosynthesis, diterpene synthase, evolution

## Abstract

**Background:**

Rabdosiae rubescentis herba (*Isodon rubescens*) is widely used as a folk medicine to treat esophageal cancer and sore throat in China. Its germplasm resources are abundant in China, with *I. rubescens* (Hemsl.) Hara and *I. rubescens* f. *lushanensis* as 2 typical forms. *I. rubescens* (Hemsl.) Hara is featured by biosynthesis of the diterpenoid oridonin with strong anticancer activity, while *I. rubescens* f. *lushanensis* produces another diterpenoid with anticancer activity, lushanrubescensin. However, the biosynthetic pathways of both still need to be fully understood. In particular, little is known about the genetic background of *I. rubescens* f. *lushanensis*.

**Findings:**

We used Pacific Biosciences (PacBio) single-molecule real-time and Nanopore Ultra-long sequencing platforms, respectively, and obtained 139.07 Gb of high-quality data, with a sequencing depth of about 328×. We also obtained a high-quality reference genome for *I. rubescens* f. *lushanensis*, with a genome size of 349 Mb and a contig N50 of 28.8 Mb. The heterozygosity of the genome is 1.7% and the repeatability is 83.43%. In total, 34,865 protein-coding genes were predicted. Moreover, we found that most of the variant or unique genes in the diterpenoid synthesis pathways of *I. rubescens* f. *lushanensis* and *I. rubescens* (Hemsl.) Hara were enriched in diterpene synthases.

**Conclusions:**

We provide the first genome sequence and gene annotation for the *I. rubescens* f. *lushanensis*, which provides molecular evidence for understanding the chemotypic differences of *I. rubescens*.

## Data Description

### Background information

*Isodon* is an important genus in Lamiaceae, a large family rich in medicinal plant resources and widely distributed worldwide. There are about 150 species in the world and more than 90 species and 25 varieties in China, of which 30 species are used for folk medicine. Nearly 300 new diterpenoids have been isolated from more than 40 species of *Isodon* [1]. Most *Isodon* are rich in *ent*-kaurane diterpenoids, featuring an α-methylene cyclopentanone structure and strong anticancer activity [2]. Among them, oridonin is one of the representative components, which is the unique medicinal ingredient for *Isodon rubescens*, reported to have good anticancer and antitumor activities, few side effects, and the capability to reduce the adverse reactions caused by chemotherapy drugs. The domestic reference quotation for this product is 180,000 to 220,000 CNY/kg, and the quotation for the international market is even higher [3].

During the survey of *I. rubescens* germplasm resources, a new form was found, named *Rabdosia rubescens* f. *lushanensis* (NCBI:txid3134017), which differs from *Rabdosia rubescens* (Hemsl.) Hara (the original form) in plant morphology [4], chemical composition [5–8], and molecular phylogenetic tree [9, 10]. The generic name of the genus has changed from *Rabdosia* to *Isodon* [11]; therefore, this article uses the new generic name (*I. rubescens* f. *lushanensis*). As for chemical composition, these 2 forms of *I. rubescens* differ in the production of diterpenoids. Especially, *I. rubescens* (Hemsl.) Hara produces oridonin while *I. rubescens* f. *lushanensis* produces lushanrubescensin with a similar tetracyclic backbone and different modifications [12]. This kind of chemotypic variation is ubiquitous in plants and is an evolutionary choice for species to adapt to their living environment in the long term. Environment and genotype factors that affect the formation of plant secondary metabolites may combinatorically lead to the generation of different chemotypic variations [13]. Since the chemical type is divided based on the chemical composition, which is based on the secondary metabolites of plants, the study of the synthetic pathways related to plant secondary metabolites and the functional genes that regulate these synthetic pathways can directly elucidate the mechanism of the formation of chemical types [14]. Therefore, it is of great significance to get a full scan of the genomic information for these 2 forms of *I. rubescens*, with a focus on the diterpenoid synthetic pathway genes, to get insight into the mechanism of chemotype formation.

A recent study of the chromosome-level genome sequencing of *I. rubescens* (Hemsl.) Hara found multiple *I. rubescens* CYP706 family proteins that can introduce hydroxyl groups at different positions on the kaurane skeleton structure, getting clues about the P450s involved in oridonin formation [15]. This known genomic information, which partially reveals the oridonin synthesis pathway of *I. rubescens* (Hemsl.) Hara in the above work, could serve as a reference for the comparative research on the genomic difference between the 2 forms of *I. rubescens*. By comparing the genes involved in GGPP cyclization and hydroxyl modification between the 2 forms, it is possible to find the unique genes responsible for the synthesis of oridonin and lushanrubescensin, respectively. In this study, telomere-to-telomere (T2T) genome sequencing was conducted for *I. rubescens* f. *lushanensis* to obtain a high-quality genome for comparative analysis with the reported genome of *I. rubescens* (Hemsl.) Hara. The discovery of new diterpenoid synthases and P450s involved in diterpenoid synthesis of *I. rubescens* in this work by comparative genomic analysis would provide insight into the chemotypic variation of *I. rubescens* and the synthesis pathway of *I. rubescens* diterpenoids.

### Plant material

*Isodon rubescens* f. *lushanensis* was sourced from Lushan County, Henan Province, China (33°72′N, 112°29′E). It was independently identified as *I. rubescens* f. *lushanensis* by Professor Suiqing Chen of Henan University of Chinese Medicine. The collected samples are preserved in the Molecular Pharmacology Laboratory of Henan University of Chinese Medicine (room number: BM628). Two types of living materials are preserved and cultivated in the medicinal botanical garden of Henan University of Chinese Medicine (available for sharing for scientific research collaboration). Plant specimens (voucher specimen number: HZYYHJY20221023 and HZYYHLS20221021) are preserved in the herbarium of Henan University of Chinese Medicine (room number:BS716). The fresh leaves were harvested and rinsed, and the surface moisture was removed. After being individually wrapped in tinfoil, they were immediately flash-frozen in liquid nitrogen for 30 minutes and stored in a −80°C refrigerator. The samples were then dispatched to Wuhan Beina Technology Co., Ltd. for sequencing.

### PacBio HiFi sequencing

High-quality genomic DNA was extracted from leaves of *I. rubescens* f. *lushanensis* following a cetyltrimethylammonium bromide (CTAB) DNA extraction protocol [16]. Then, 0.75% agarose gel electrophoresis was used to detect the size of DNA fragments and the degree of DNA degradation, a NanoDrop One spectrophotometer (Thermo Fisher Scientific) was used to detect DNA purity, and a Qubit 3.0 fluorescence analyzer (Life Technologies) was used to detect DNA concentration and accurately quantify DNA. Subsequently, a PCR-free SMRT bell library was constructed, and the library template and enzyme complex were transferred to the nanopores of the Sequel II sequencer (PacBio) for sequencing. The data were read using the official PacBio software (SMRTlink v8.0) for quality control and statistical analysis of the output data. The raw data were filtered using CCS v6.0.0 (RRID:SCR_021174) to obtain valid data for subsequent analysis. A total of 1,604,661 PacBio postfiltered reads were generated. This produced 30.55 Gb of single-molecule sequencing data, with an average read length of 19,037 bp (Supplementary Fig. S1 and Supplementary Table S1).

### Nanopore ONT Ultra-long

ONT sequencing was a single-molecule real-time sequencing technology that relied on nanopore electrical signals. Under the influence of motor proteins, DNA double strands bound to octamer nanopore proteins anchored to the biofilm and unwound [17]. Owing to a potential difference across the biofilm, the unwound single strands of DNA moved through the nanopores at a set speed. Due to their distinct chemical properties, as different bases passed through the nanopores, they triggered variations in electrical signals [18]. The detection and interpretation of these signal variations allowed for real-time sequencing [19]. High-quality DNA samples were extracted and tested for purity, concentration, and integrity. The purified product was then linked using sequencing adapters from the assay kit (SQK-LSK109). Once constructed, the DNA library was loaded into the Flow cell and transferred to the Oxford Nanopore sequencer (PromethION) for real-time single-molecule sequencing. The raw data were filtered using Filtlong v0.2.4 (RRID:SCR_024020) to obtain valid data for subsequent analysis. A total of 465,394 reads were generated. This produced 19.37 Gb of raw sequencing data, with an average cleaned read length of 41,620.42 bp (Supplementary Fig. S2 and Supplementary Table S2).

### Estimation of genome size, heterozygosity, and repeat content

This experiment employed the *k*-mer–based analysis method to estimate genome size and heterozygosity rates. The genome size was assessed using GCE v1.0.0 (RRID:SCR_017332) [20], Frequency-depth distribution was obtained by Jellyfish v2.2.10 (RRID:SCR_005491) [21], and the genome size was estimated based on the *k*-mer frequency-depth distribution. In this study, 98,556,357,522 *k*-mers were generated, and the peak *k*-mer depth was 282.355 (Supplementary Fig. S3). The genome size was estimated to be approximately 349 Mb, and the final cleaned data corresponded to the coverage of about 328-fold. Repeat and heterozygosity rates were estimated at 83.43% and 1.7%, respectively (Supplementary Table S3).

### Genome assembly of *I. rubescens* f. *lushanensis*

This study used 2 sequencing techniques (Nanopore ONT Ultra-long and PacBio HiFi) to assemble the T2T genome separately. Next-Denovo v2.5.0 (RRID:SCR_025033) [22], NECAT v202000119 (RRID:SCR_025350) [23], Flye v2.9-b1768 (RRID:SCR_017016) [24], and Hifiasm v0.16.1 (RRID:SCR_021069) [25] genome assembly software were used to preliminarily assemble ONT ultra-long and HiFi sequencing data, respectively. The preliminary assembly results of ONT ultra-long/PacBio HiFi data were evaluated for quality value (qv), and the results showed that the qv of genome quality was 54.704, the assembled genome length was 382,388,585 bp, and the contig N50 was 28,807,450 bp (Supplementary Table S4). Winnowmap v1.11 (RRID:SCR_025349) [26] was used to fill in gaps in the genome gap interval and then align with the gap-filled version of the genome (HiFi reads ≥10 kbp). Then samtools v1.10 (RRID:SCR_002105) [27] was used to filter the aligned fragments, and finally Racon v1.6.0 (RRID:SCR_017642) was used for error correction to obtain the final T2T version genome. We used the software BUSCO v4.14 (RRID:SCR_015008) [28] to evaluate the integrity of the genome assembly (Supplementary Table S5). We identified whether our previous operations were correct by checking the abnormality of the Hi-C interaction heatmap signal. In this experiment, a total of 375,924,825 bp of genome sequence were mapped to 12 chromosomes, accounting for 100% (Supplementary Fig. S4 and Supplementary Table S6).

### T2T genome annotation of *I. rubescens* f. *lushanensis*

The annotation of genome repeat sequences began with the *de novo* method, employing software RepeatModeler v1.0.11 (RRID:SCR_015027) [29] to predict model sequences based on the genome sequence. Additionally, software LTR_Finder v1.2 (RRID:SCR_015247) [30] was utilized to predict long terminal repeat (LTR) sequences, followed by LTR_retriever (RRID:SCR_017623) [31] to eliminate redundancy in the predicted sequences to produce nonredundant LTR sequences. These 2 *de novo* sequences were combined to form the *de novo* repeat sequence library. Subsequently, the RepBase library [32] was integrated with the *de novo* library [33] and aligned using RepeatMasker v4.0.9 (RRID:SCR_012954) [34] to predict repeat sequences and generate the *de novo* + RepBase results. RepeatProteinMask (subsoftware of RepeatMasker) was employed to predict repeat sequences of the TE_proteion type, yielding transposable element (TE) protein results. Finally, all predicted repeat sequences were merged and redundant elements were eliminated to obtain the final set of genome repeat sequences (combined TEs). This analysis found that the total repetitive sequences in the genome of *I. rubescens* f. *lushanensis* accounted for 58.47% of the entire genome (Supplementary Fig. S5 and Supplementary Table S7).

In this study, transcriptome prediction, homology prediction, and *ab initio* prediction were used to predict the gene structure, the full-length sequences obtained by Nanopore ONT Ultra-long and PacBio Hifi sequencing were compared with the genome using Minimap2 v2.17-r941 (RRID:SCR_018550) [35, 36], and then the bam files of the comparison results were combined and the transcripts were reconstructed by StringTie v2.1.4 (RRID:SCR_016323) [37]. Then the predicted transcripts were predicted by TransDecoder v5.1.0 (RRID:SCR_017647) to predict the coding box, and finally the predicted coding genes were obtained. Homology prediction selected the protein sequence files of near-source species for prediction analysis, and the homologous protein sequences were aligned to the genome using Tblastn v2.7.1 (RRID:SCR_011822) [38], and then Exonerate v2.4.0 (RRID:SCR_016088) [39, 40] was used to predict transcripts and coding regions based on the comparison results, and Augustus v3.3.2 (RRID:SCR_008417) [41] was used for *ab initio* prediction. GlimmerHMM v3.0.4 (RRID:SCR_002654) [42] performed *de novo* prediction based on the results of homologous prediction. Finally, MAKER v2.31.10 (RRID:SCR_005309) [43] was used to integrate the genes predicted by various methods. A total of 34,865 genes were predicted in the T2T genome structure prediction, with an average mRNA length of 3,787.28 bp and an average CDS length of 1,163.68 bp (Supplementary Table S8). The predicted genes were annotated against several functional databases, including the NCBI nonredundant protein database (NR) [44], the KEGG database [45], the Uniprot database [46], the Interpro database [47], the protein families database (Pfam) [48], the Cluster of Orthologous Groups for eukaryotic complete genomes (KOG) [49] database, and Gene Ontology (GO) [50, 51]. It was found that 94.32% of all predicted genes could be annotated with the following databases: KEGG (22.56%), KEGG Pathway (17.73%), NR (89.15%), Uniprot (88.12%), GO (65.21%), KOG (3.20%), Pfam (64.55%), and Interpro (92.06%) (Supplementary Table S9).

### Comparison of genome assembly and annotation information

**Figure 1:**
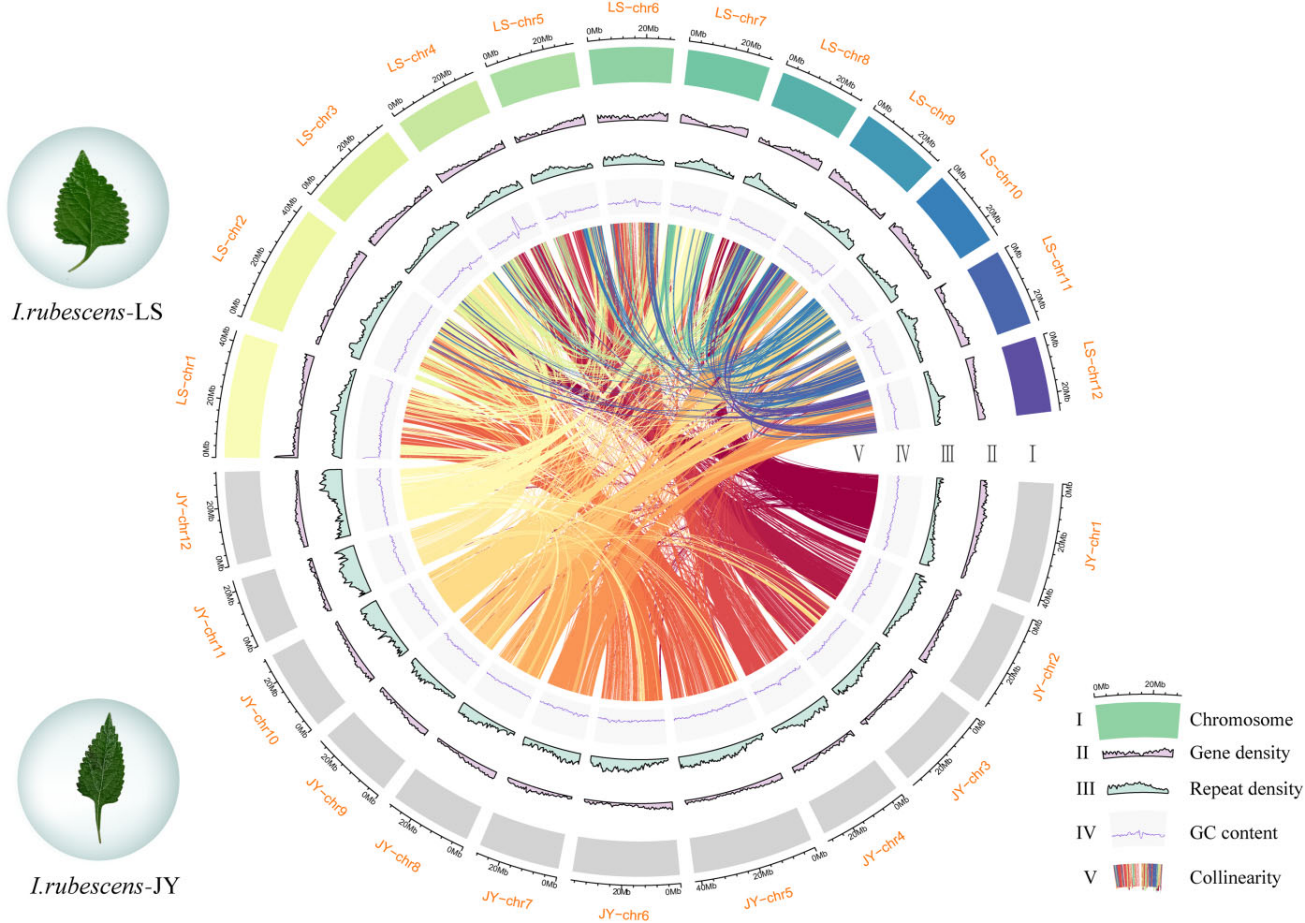
Genome assembly characterization and chromosome locations of *I. rubescens* f. *lushanensis* and *I. rubescens* (Hemsl.) Hara. Landscape of *I. rubescens* f. *lushanensis* and *I. rubescens* (Hemsl.) Hara genomes: I chromosome, II gene density, III repeat density, Ⅳ GC content, and V collinearity between and within *I. rubescens* f. *lushanensis* and *I. rubescens* (Hemsl.) Hara genomes.

This assembly of *I. rubescens* f. *lushanensis* (hereafter mentioned as *I. rubescens*-LS) represents the highest continuity and completeness compared with the recently released genome assemblies for the published genome of *I. rubescens* (Hemsl.) Hara (hereafter mentioned as *I. rubescens*-JY) [15]. In order to clearly display the genomic information of the 2 forms of *I. rubescens*, we conducted a comparative analysis of their genomic information. Based on *k*-mer distribution analysis, the genome size of *I. rubescens*-LS was estimated to be 349 MB, with a heterozygosity of 1.7%. In comparison, the genome size of *I. rubescens*-JY was 384 MB, and the heterozygosity was 1.6%. The total data volume generated by *I. rubescens*-LS was 139.07 Gb, much more than that of *I. rubescens*-JY (55.21 Gb). Relying on the Hi-C technology, a total of 89.15Gb of data was obtained for *I. rubescens*-LS. All bases obtained by sequencing were attached to 12 chromosomes, with coverage rates of 100% and 94.37% for *I. rubescens*-LS and *I. rubescens*-JY, respectively [15]. The genome contig N50 of *I. rubescens*-LS was 28.8 MB, much longer than that of *I. rubescens*-JY (0.3 MB). In addition, BUSCO analysis between *I. rubescens-*LS and *I. rubescens*-JY showed that the completeness of the genome assembly was 94.4% and 88% (Table 1), respectively. These results demonstrated the outstanding quality of the genome assembled for *I. rubescens*-LS, which together with the published *I. rubescens*-JY genome laid the foundation for comparative genomic analysis of these 2 forms (Fig. 1).

**Table 1:** Summary of genome assembly of *I. rubescens*-LS and comparison with the *I. rubescens*-JY genome

Item	*I. rubescens*-LS	*I. rubescens*-JY
Size of genome (MB)	349	384
Total_Data (Gb)	139.07	55.21
Contig N50 (MB)	28.8	0.3
Gene chromosome coverage (%)	100	94.3
Heterozygosity rate (%)	1.7	1.6
GC_content (%)	35.95	N
Pseudochromosomes	12	12
Percentage of LTR (%)	58.47	55
Protein-coding genes	34,865	35,436
Non-coding RNA	8,032	3,456
BUSCO (%)	98.9	88
Reference	This study	[15]

The BUSCO evaluation values of the *I. rubescens*-LS and *I. rubescens*-JY genome annotation results were both higher than 90%, proving that the annotation results were reliable (Supplementary Table S5). The total numbers of protein-coding genes in *I. rubescens*-LS and *I. rubescens*-JY were 34,865 and 30,789, the average lengths of the mRNAs were 4,110.25 bp and 3,711.47 bp, and the average lengths of CDS were 1,209.58 bp and 1,200.50 bp, respectively (Supplementary Table S8). The proportions of repetitive sequences in the entire genome of *I. rubescens*-LS and *I. rubescens*-JY were 58.47% and 55%, respectively. The main repeat type in both genomes was LTRs, with proportions of 28.98% and 25.15%, respectively. The main retrotransposons were LTR/*Copia* and LTR/*Gypsy*, with 7.57% of LTR/*Copia* and 17.35% of LTR/*Gypsy* in *I. rubescens*-LS and 7.56% of LTR/*Copia* and 14.07% of LTR/*Gypsy* in *I. rubescens*-JY (Supplementary Fig. S6A). A total of 8,032 and 3,456 noncoding RNAs were found in *I. rubescens*-LS and *I. rubescens*-JY, respectively. There was a significant difference in the amount of ribosomal RNAs (rRNAs) between the 2 *I. rubescens* forms, which was specifically reflected in the 5S RNA amount (Supplementary Fig. S6B). With the functions of constituting ribosomes, catalyzing protein synthesis, and recognizing promoters and stops, rRNAs played a crucial role in protein synthesis and were generally regarded as conservative. Therefore, the different number of rRNAs annotated might be caused by the difference in the completeness of the 2 genome assemblies.

### Identification and analysis of orthologous genes

**Figure 2:**
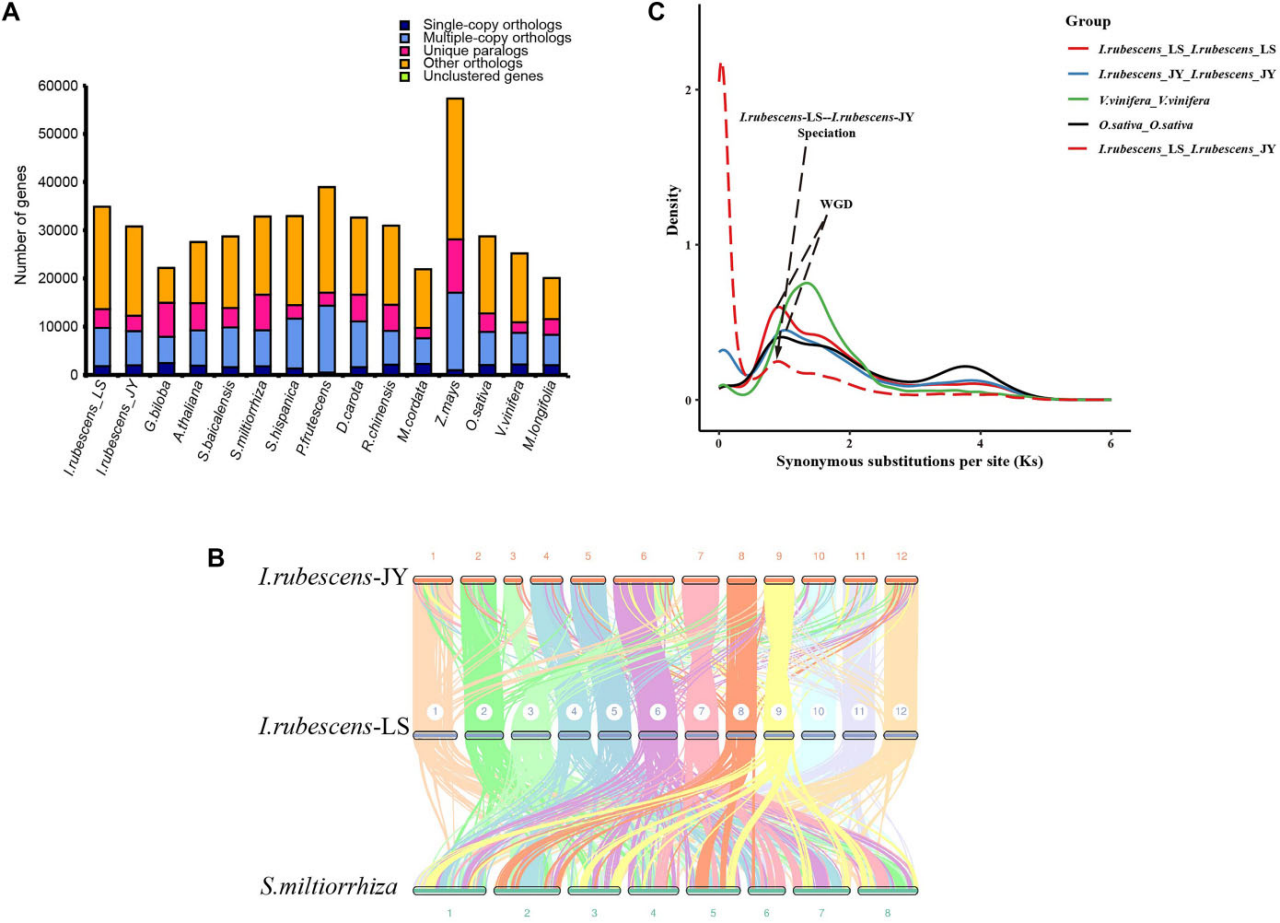
Genome-wide collinearity analysis and KS analysis of *I. rubescens*. (A) Number of homologous genes in different species. (B) Collinearity analysis between *I. rubescens* f. *lushanensis, I. rubescens* (Hemsl.) Hara, and *S. miltiorrhiza* chromosomes. (C) Curve-fitting analysis of the distribution of synonymous substitution rates (Ks) of homologous genes.

Identification of homologous genes was a very important aspect of evolutionary analysis. First, based on all amino acid sequences of the selected species, Orthofinder v2.3.12 (RRID:SCR_017118) [52] was used to cluster gene families, and Blastp v2.6.0 (RRID:SCR_001010) [38] was used for comparison. The statistical analysis of gene family identification results showed that a total of 55,076 orthologous gene families were found in all species, including 465,541 genes, among which the number of single-copy genes was 158, and the number of gene families common to all species was 4,119, including 151,827 genes. There were 2,817 gene families unique for *I. rubescens*-LS, including 3,920 genes (Fig. 2A). This analysis used Last v1170 (RRID:SCR_006119) [53] to compare the gene sequences of *I. rubescens*-LS and *I. rubescens*-JY to determine similar gene pairs. Then, JCVI v0.9.13 (RRID:SCR_011269) [54] was used to determine whether similar gene pairs were adjacent on the chromosome according to the annotation file (gff3) to finally obtain the genes in all collinear blocks. The collinearity maps of *I. rubescens*-LS, *I. rubescens*-JY, and *Salvia miltiorrhiza* suggested that a whole-genome duplication event occurred between the 2 forms of *I. rubescens* (Fig. 2B). The number of shared gene families between *I. rubescens*-LS and *I. rubescens*-JY accounts for 75% of the total gene families in their genomes. The significant overlap in the gene families suggested that the 2 forms of *I. rubescens* were closely related (Supplementary Fig. S7A). Most genes were conserved in *I. rubescens*-LS and *I. rubescens*-JY, implying that specific and unique gene families might cause differences in metabolites. To verify this assumption, Clusterprofiler v3.19 (RRID:SCR_016884) [55] was used to perform on the unique gene family members found in the 2 forms of *I. rubescens* (Supplementary Fig. S8A, B), screening for genes enriched in the diterpenoid synthesis pathway, followed by locating them on chromosomes. It turned out that 14 unique genes in *I. rubescens*-JY and *I. rubescens*-LS are enriched in the diterpenoid synthesis pathway (Supplementary File 1, Supplementary Fig. S7B, C). This result revealed that the abundant variations in diterpene synthetic genes might contribute to the chemotypic variation of *I. rubescens*-LS and *I. rubescens*-JY.

### Phylogenetic and duplication analysis of whole-genome

**Figure 3:**
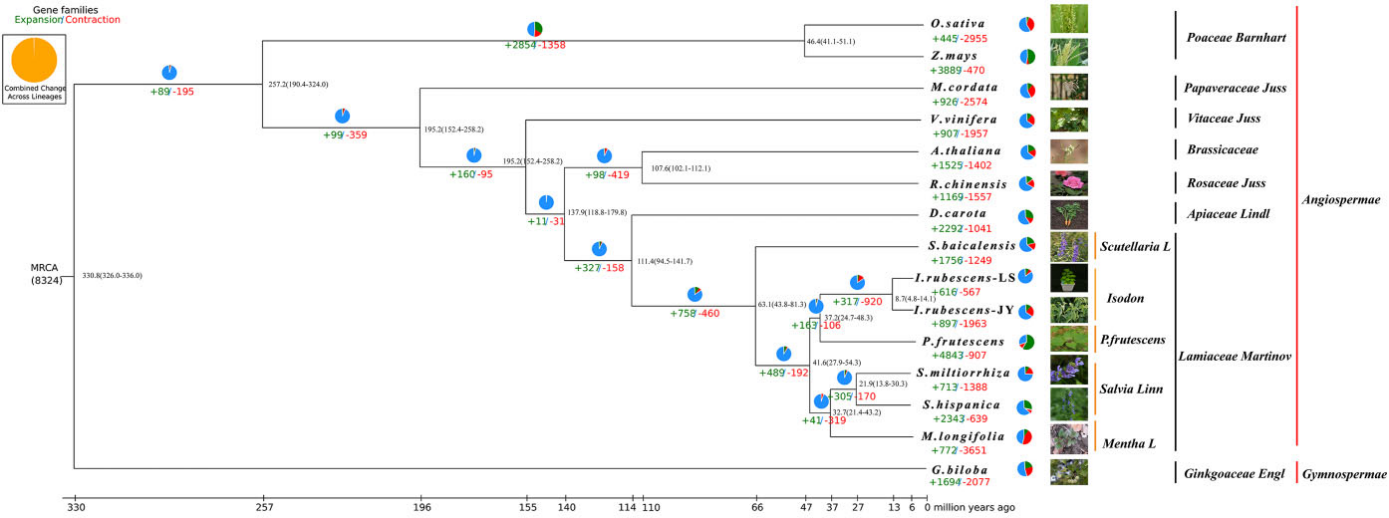
Inferred phylogenetic tree with 158 single-copy genes from 15 plant species (or forms). Gene family expansions are indicated in green, and gene family contractions are indicated in red. The timing of WGD is superimposed on the tree. Divergence times were estimated by maximum likelihood (PAML).

The software Muscle v3.8.31 (RRID:SCR_011812) [56] was used to perform multiple sequence alignment of protein sequences of each single-copy gene family, and then Trimal v1.2rev59 (RRID:SCR_017334) [57] was used to filter the alignment results, and then the filtered alignment results were combined. Finally, the maximum likelihood (ML) species phylogenetic tree was constructed based on the merged results using RAXML v8.2.10 (RRID:SCR_006086) [58]. Based on the topological structure of the phylogenetic tree and fossil time node table [59], MCMCtreeR v4.9 (RRID:SCR_025348) was used to estimate the differentiation time of selected species. The current classification of *I. rubescens*-LS and *I. rubescens*-JY was based on plant classification and chemical composition classification. Phylogenetic analysis based on whole-genome data was expected to more accurately and objectively determine the genetic relationship between the 2 forms of *I. rubescens*. For this purpose, genomes of 15 representative plants with good assembly quality and located in different evolutionary branches were downloaded from the NCBI database to conduct a comparative analysis of *I. rubescens*-LS and *I. rubescens*-JY from an evolutionary perspective (Supplementary Table S10). These species were distributed in 8 major families, including the gymnosperm *Ginkgo biloba* (maidenhair tree) and the angiosperms *Arabidopsis thaliana* (thale cress), *Scutellaria baicalensis* (Baikal skullcap), *S. miltiorrhiza* (Chinese salvia), *Mentha longifolia* (horsemint), *Salvia hispanica, Perilla frutescens* (beefsteak-mint), *Daucus carota* subsp. *Sativus, Rosa chinensis* (China rose), *Macleaya cordata, Zea mays* subsp*. mays* (maize), *Oryza sativa* (Asian cultivated rice), and *Vitis vinifera* (wine grape). Among these, thale cress, rice, and grape were included due to their well-documented background of the whole-genome doubling (WGD) events, which can serve as reference species in later WGD analysis. Genome phylogenetic analysis showed that 7 species (or forms) from the family Lamiaceae were clustered as monophyletic. Among them, *S. baicalensis* was first separated from the others, and the remaining 6 species (or forms) were divided into 2 categories (*S. miltiorrhiza, M. longifolia*, and *S. hispanica; Perilla frutescens, I. rubescens*-LS, and *I. rubescens*-JY). *I. rubescens* and *Perilla* separated the latest, suggesting a close genetic relationship between them. This was consistent with the results of a previous phylogenetic study on common genera of labialis [60]. *I. rubescens*-LS and *I. rubescens*-JY belong to separate groups. CAFE v3.1 (RRID:SCR_005983) [61] was used to estimate the number of gene family members in the ancestor of each branch based on the species evolutionary tree and gene family clustering results. Compared with their most recent common ancestor (MRCA), gene families had obviously contracted. Among them, the numbers of contractions of the *I. rubescens*-LS and *I. rubescens*-JY gene families were 567 and 1,963, and the numbers of expanded gene families were 616 and 897, respectively. These numbers were relatively lower than most other Lamiaceae species, suggesting that *I. rubescens* was relatively conservative compared to other plants of the Lamiaceae family (Fig. 3).

In order to have a clearer and more in-depth understanding of the whole-genome duplication event in *I. rubescens*, PAML v4.9 (RRID:SCR_014932) [62] was used to calculate the ratio of Ka/Ks, and ggplot2 v2.2.1 (RRID:SCR_014601) [63] was used to draw a density map. Grape and rice, the classic species of genome doubling events, were used as references for Ks curve-fitting analysis (Fig. 2C); it was found that the median Ks curves of *I. rubescens*-LS and *I. rubescens*-JY peaked at the same position. In addition, the peak of the separation curves and the peak of the whole-genome duplication event overlap were also at the same position. Taking together the results of phylogeny and divergence time analysis, we could reasonably speculate that a whole-genome duplication event occurred between *I. rubescens*-LS and *I. rubescens*-JY, which led to evolutionary differentiation between the 2 forms. The multiplied genes had multiple possibilities such as pseudogenization, neofunctionalization, and subfunctionalization. Among them, the neofunctionalization and pseudogenization genes might lead to the occurrence or disappearance of unique metabolites, thus causing the chemotypic variation of *I. rubescens*.

### Comparative analysis of genomic structure variations

**Figure 4:**
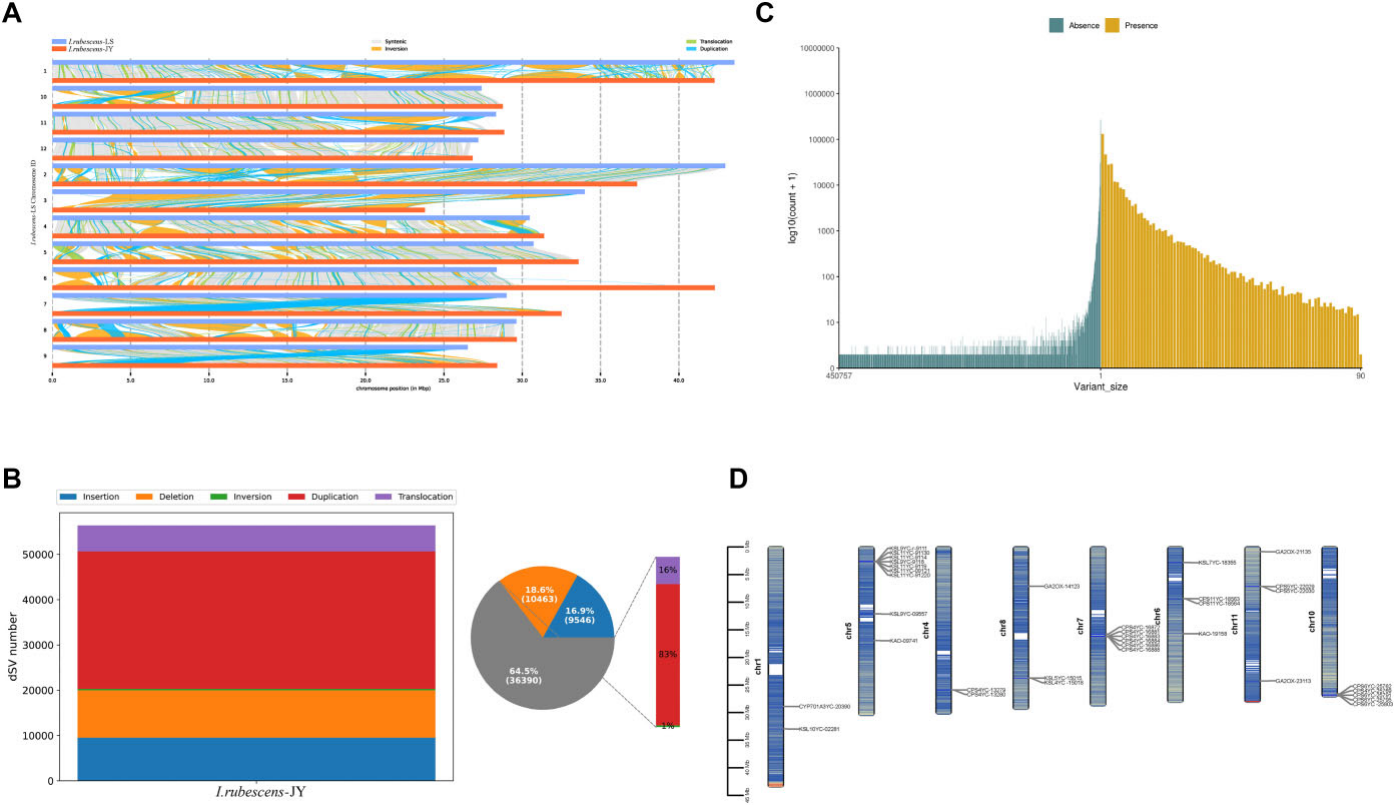
Analysis of whole-genome structural variation of *I. rubescens* f. *lushanensis* and *I. rubescens* (Hemsl.) Hara. (A) Collinear syri diagram of *I. rubescens* f. *lushanensis* and *I. rubescens* (Hemsl.) Hara. The blue skeleton displays the *I. rubescens* f. *lushanensis* genome, the red skeleton displays the *I. rubescens* (Hemsl.) Hara genome, and the middle displays the collinearity and identified SV distribution. (B) Statistical results of SV types and quantities. (C) Length distribution of PAVs. (D) Chromosome distribution map of genes affected by SV and PAV on the reference genome.

MUMmer v4.0.0rc1 (RRID:SCR_018171) [64] and SyRI v1.6 (RRID:SCR_023008) [65] were used to conduct whole-genome comparison and mutation type detection, respectively, using the genome of *I. rubescens-*LS as the reference genome. TBtools v2.069 (RRID:SCR_023018) [66] was used to annotate gene function and pathway enrichment and map the distribution of gene family genes on the chromosome. *I. rubescens*-LS and *I. rubescens*-JY were both diploid (2n = 24). The *I. rubescens*-LS genome with higher genome assembly quality was used as the reference genome to detect presence/absence variations (PAVs) and structure variations (SVs). The whole genomes of *I. rubescens*-LS and *I. rubescens*-JY corresponding to a total of 367,304,545 bp were used in the collinear sequence alignment, with a coverage rate of 95.68% (Fig. 4A). SVs usually referred to large-scale sequence changes and positional relationship changes on the genome, including long-segment chromosomal inversions and chromosomal translocations and duplication, which had a more significant impact on the genome. In humans, such structural variants were associated with many diseases (including autism, obesity, schizophrenia, cancer, etc.) [67]. In plants, SVs were associated with many phenotypic variations and biotic/abiotic stresses [68]. A total of 56,399 SVs were found in this study, mainly distributed in intergenic regions. The predominant type was repeated mutations, accounting for 53.7% (Fig. 4B). Another important type of structural variation was PAV, which might be why individuals produced different traits (disease resistance, cold resistance, etc.) [69]. A total of 34,443 PAVs were found, with almost equal numbers of presence and absence mutations, and the length span of the mutations was significant (Fig. 4C). Genes affected by PAVs and SVs were identified and subjected to pathway functional enrichment analysis (Supplementary Fig. S9A, B). Genes enriched in the diterpenoid synthesis pathway were annotated and located on the chromosome for visual analysis (Supplementary File 2 and Fig. 4D). The results showed the distribution of 35 mutated genes on 8 chromosomes, most of which were diterpene synthase genes, together with a CYP450 encoding gene and 5 genes of the gibberellin synthesis pathway. This result supported the speculation that the variations in diterpenoid synthetic genes might be one of the driving forces for the chemotypic variation of *I. rubescens*.

## Discussion

The biosynthesis of active ingredients in traditional Chinese medicine had emerged as a research focus in recent years. However, it was often hindered by the unclear molecular mechanism and pathway for synthesizing the target natural product. Analysis of the complete genome information could help us understand the evolutionary relationship among medicinal plants with different types or contents of active ingredients and shed light on the biosynthesis pathway and its regulation mechanism [70]. Due to differences in genome duplication, transposon expansion, and repetitive sequences, genome sizes varied widely among different species. Around 16 medicinal plants in the Lamiaceae family had completed genome sequencing, and their size ranged from 338 Mb to 2.07 G (Supplementary Table S11). Most of the genome sequencing results included incomplete assembly of gaps, centromere regions, and telomere regions, as well as high heterozygosity and high repetitive assembly errors or incompleteness. Recently, T2T genome sequencing employing multiple sequencing platforms and high-depth sequencing had emerged to assemble gap-free or near-gap-free high-quality genomes. This sequencing method overcame the difficulty of assembling centromeres or highly repetitive regions and greatly improved the continuity and integrity of chromosomes [71, 72]. In the present study, the complete genome information of *I. rubescens*-LS was revealed for the first time, with a size of 349 Mb and a GC content of 35.78%, similar to that of the *I. rubescens-*JY previously sequenced. The high genome heterozygosity (1.7%) and duplication degree (83.43%) suggested that the genome of *I. rubescens*-LS is highly heterozygous and highly repetitive. Generally, a genome with a heterozygous rate >0.5% and a repetitive sequence content >50% is considered a highly heterozygous and highly repetitive genome [73]. The quality of the *I. rubescens*-LS genome obtained using T2T genome sequencing had been dramatically improved compared with the previous sequenced *I. rubescens*-JY genome. The successful sequencing annotation of the complete genome sequences of the 2 closely related *I. rubescens* forms would provide the basis for understanding the origin of the chemotypic variation and meanwhile supply genetic elements for reconstruction of the synthetic pathway for valuable plant natural products in model classes.

Whole-genome evolution helped to understand the genetic relationship between the 2 forms of *I. rubescens*. The number of gene families shared by *I. rubescens*-LS and *I. rubescens*-JY was 17,632, accounting for 75.4% of the total number of families, proving that most genes of the 2 forms were derived from the same ancestor. They had apparent similarities in structure and function, encoding similar proteins and performing similar functions. As an important driving factor for the emergence of new traits and functions, whole-genome duplication events occurred throughout the entire plant evolution process. Both *I. rubescens*-LS and *I. rubescens*-JY experienced a whole-genome duplication event, and the whole-gene duplication peaks and the speciation peak of these 2 forms coincided. *I. rubescens*-LS and *I. rubescens*-JY diverged. Taken together with the species divergence time, it could be reasonably inferred that a whole-genome duplication event occurred between the 2 forms of *I. rubescens*, leading to their separation. Through genome-wide evolutionary analysis, we provided a molecular basis for the phylogenetic classification of *I. rubescens-*JY and *I. rubescens-*LS. This result supported current plant and chemical taxonomy. Variations covering SV and PAV were found in the 2 forms. SVs and PAVs were more enriched in the diterpenoid synthesis pathway, particularly in diterpene synthase genes. Consistent with the results of the gene variation analysis, the results of the unique gene family analysis of *I. rubescens*-JY and *I. rubescens*-LS showed that most of the unique genes enriched in the diterpenoid synthesis pathway were diterpene synthase genes. Therefore, we could carry out functional verification analysis on these diterpene synthase genes affected by structural variation in the later stage and provided more of a molecular basis for the analysis of the diterpene biosynthesis pathway and chemical-type variation of related species in *I. rubescens*.

## Supplementary Material

giae075_Supplemental_Files

giae075_GIGA-D-24-00177_Original_Submission

giae075_GIGA-D-24-00177_Revision_1

giae075_Response_to_Reviewer_Comments_Original_Submission

giae075_Reviewer_1_Report_Original_Submission Henrik Toft Simonsen -- 6/19/2024

giae075_Reviewer_1_Report_Revision_1 Henrik Toft Simonsen -- 7/23/2024

giae075_Reviewer_2_Report_Original_Submission Wei Sun -- 7/1/2024

## Data Availability

The data supporting the findings of this work are available in the article and its supplementary information file. Genome Data NCBI BioProject number: PRJNA1089255 and BioSample accession: SAMN40534968. All additional supporting data are available in the *GigaScience* repository, GigaDB [74].

## Additional Files

**Supplementary Fig. S1**. HiFi data length statistical histogram.

**Supplementary Fig. S2**. Histogram showing distribution of read lengths across quality passing sequences. The 2 black vertical lines from left to right are the average length and N50 of all data, and different colors correspond to pass reads and fail reads, respectively.

**Supplementary Fig. S3**. The *k*-mer frequency distribution at *k*-mer size of 19.

**Supplementary Fig. S4**. Interval interaction diagram in the chromosome. The blue border represents each chromosome.

**Supplementary Fig. S5**. Statistical map of genome repeat sequence distribution. DNA: DNA transposons; LINE: long interspersed nuclear element; LTR: long terminal repeat; SINE: short interspersed element.

**Supplementary Fig. S6**. Genomic repeated sequences and noncoding RNA annotation information of *I. rubescens*-LS and *I. rubescens*-JY. (A) Comparison of genomic repeated sequences of *I. rubescens*-LS and *I. rubescens*-JY. (B) Comparison of noncoding RNAs in *I. rubescens*-LS and *I. rubescens*-JY.

**Supplementary Fig. S7**. Analysis of unique gene families in *I. rubescens*-LS and *I. rubescens*-JY. (A) Shared and unique gene families in the *I. rubescens*-LS and *I. rubescens*-JY genomes. (B) Chromosome distribution map of unique diterpenoid pathway genes in *I. rubescens*-JY. (C) Chromosome distribution map of unique diterpenoid pathway genes in *I. rubescens*-LS.

**Supplementary Fig. S8**. Bubble plot reference for KEGG enrichment analysis of unique genes in *I. rubescens*-LS and *I. rubescens*-JY. (A) Bubble plot reference for KEGG enrichment analysis of unique genes in *I. rubescens*-LS. (B) Bubble plot reference for KEGG enrichment analysis of unique genes in *I. rubescens*-JY.

**Supplementary Fig. S9**. KEGG enrichment analysis of SV and PAV. (A) Bubble plot reference for KEGG enrichment analysis of genes affected by SV on the reference genome. (B) Bubble plot reference for KEGG enrichment analysis of genes affected by PAV on the reference genome.

**Supplementary Table S1**. Statistical table of data volume information of the third-generation sequencing (ONT ultra long). Rank is the data length gradient, >0 is all data. Flag is the data type, all is the total sequencing data, pass is the valid sequencing data, and fail is the filtered data.

**Supplementary Table S2**. Statistical table of PacBio Hifi.

**Supplementary Table S3**. Estimation of genome characteristics based on 19-mer statistics.

**Supplementary Table S4**. Statistical table of QV value of genome assembly.

**Supplementary Table S5**. Comparative analysis of BUSCO annotated genome structure of *I. rubescens*-LS and *I. rubescens*-JY.

**Supplementary Table S6**. Statistical map of genome chromosome length distribution.

**Supplementary Table S7**. Genome repeat sequence distribution of *I. rubescens*-LS.

**Supplementary Table S8**. Comparison of genomic protein-encoding gene annotations of *I. rubescens*-LS and *I. rubescens*-JY.

**Supplementary Table S9**. Statistics of functional annotation results of coding genes.

**Supplementary Table S10**. Sequence information used in phylogenetic tree analysis for genome evolution.

**Supplementary Table S11**. Genome information of medicinal plants of Lamiaceae.

**Supplementary File 1**. Statistical table of functional enrichment analysis of specific gene family of *I. rubescens*-JY and *I. rubescens*-LS.

**Supplementary File 2**. Functional enrichment analysis of diterpenoid synthesis pathway genes in *I. rubescens*-LS under PVA and SV influence.

## Abbreviations

BUSCO: Benchmarking Universal Single-Copy Orthologs; CTAB: cetyltrimethylammonium bromide; GO: Gene Ontology; KEGG: Kyoto Encyclopedia of Genes and Genomes; KOG: Cluster of Orthologous Groups for eukaryotic complete genomes; LTR: long terminal repeat; MRCA: most recent common ancestor; NCBI: National Center for Biotechnology Information; NR: nonredundant protein database; PAV: presence/absence variation; rRNA: ribosomal RNA; SV: structure variation; T2T: telomere-to-telomere; WGD: whole-genome doubling.
