# Supplementary material for: High-quality assembly of the T2T genome for Isodon rubescens f. lushanensis reveals genomic structure variations between 2 typical forms of Isodon rubescens
Source: Gigascience. 2024 Oct 10;13:giae075. doi: 10.1093/gigascience/giae075 (PMC11466039; doi:10.1093/gigascience/giae075)
Supplement: giae075_Supplemental_Files [file giae075_supplemental_files.zip › Table_S1.docx]

| Rank | Flag | TotalBase | TotalReads | MaxLen | AvgLen | N50 | meanQ |
| --- | --- | --- | --- | --- | --- | --- | --- |
| >0 | all | 20,122,556,902 | 482,055 | 1,132,566 | 41,743.28 | 50,000 | 12.05 |
| >0 | pass | 19,369,891,879 | 465,394 | 419,129 | 41,620.42 | 50,000 | 12.29 |
| >0 | fail | 752,665,023 | 16,661 | 1,132,566 | 45,175.26 | 50,004 | 5.45 |
| >10000 | all | 20,122,556,902 | 482,055 | 1,132,566 | 41,743.28 | 50,000 | 12.05 |
| >10000 | pass | 19,369,891,879 | 465,394 | 419,129 | 41,620.42 | 50,000 | 12.29 |
| >10000 | fail | 752,665,023 | 16,661 | 1,132,566 | 45,175.26 | 50,004 | 5.45 |
| >20000 | all | 18,636,989,577 | 394,788 | 1,132,566 | 47,207.59 | 52,949 | 11.98 |
| >20000 | pass | 17,884,324,554 | 378,127 | 419,129 | 47,297.14 | 53,070 | 12.27 |
| >20000 | fail | 752,665,023 | 16,661 | 1,132,566 | 45,175.26 | 50,004 | 5.45 |
| >30000 | all | 15,770,675,522 | 278,923 | 1,132,566 | 56,541.32 | 59,215 | 11.97 |
| >30000 | pass | 15,155,912,158 | 267,792 | 419,129 | 56,595.84 | 59,311 | 12.24 |
| >30000 | fail | 614,763,364 | 11,131 | 1,132,566 | 55,229.84 | 57,129 | 5.47 |
| >40000 | all | 12,818,233,146 | 193,922 | 1,132,566 | 66,099.94 | 66,775 | 11.94 |
| >40000 | pass | 12,331,197,894 | 186,471 | 419,129 | 66,129.31 | 66,856 | 12.20 |
| >40000 | fail | 487,035,252 | 7,451 | 1,132,566 | 65,365.09 | 64,745 | 5.49 |
| >50000 | all | 10,061,393,279 | 132,278 | 1,132,566 | 76,062.48 | 75,479 | 11.91 |
| >50000 | pass | 9,685,054,533 | 127,309 | 419,129 | 76,075.18 | 75,530 | 12.16 |
| >50000 | fail | 376,338,746 | 4,969 | 1,132,566 | 75,737.32 | 73,963 | 5.47 |
| >60000 | all | 7,723,180,891 | 89,508 | 1,132,566 | 86,284.81 | 84,632 | 11.88 |
| >60000 | pass | 7,440,484,157 | 86,251 | 419,129 | 86,265.48 | 84,636 | 12.12 |
| >60000 | fail | 282,696,734 | 3,257 | 1,132,566 | 86,796.66 | 84,401 | 5.48 |
| >70000 | all | 5,866,171,170 | 60,777 | 1,132,566 | 96,519.59 | 94,220 | 11.85 |
| >70000 | pass | 5,654,973,809 | 58,630 | 419,129 | 96,451.88 | 94,176 | 12.09 |
| >70000 | fail | 211,197,361 | 2,147 | 1,132,566 | 98,368.59 | 95,562 | 5.46 |
| >80000 | all | 4,422,251,100 | 41,446 | 1,132,566 | 106,699.1 | 103,864 | 11.81 |
| >80000 | pass | 4,261,475,928 | 39,975 | 419,129 | 106,603.53 | 103,787 | 12.04 |
| >80000 | fail | 160,775,172 | 1,471 | 1,132,566 | 109,296.51 | 106,261 | 5.49 |
| >90000 | all | 3,313,181,041 | 28,342 | 1,132,566 | 116,900.04 | 113,493 | 11.78 |
| >90000 | pass | 3,190,660,956 | 27,324 | 419,129 | 116,771.37 | 113,426 | 12.01 |
| >90000 | fail | 122,520,085 | 1,018 | 1,132,566 | 120,353.72 | 114,889 | 5.46 |
| >100000 | all | 2,477,198,751 | 19,513 | 1,132,566 | 126,951.2 | 122,986 | 11.74 |
| >100000 | pass | 2,383,220,092 | 18,797 | 419,129 | 126,787.26 | 122,938 | 11.98 |
| >100000 | fail | 93,978,659 | 716 | 1,132,566 | 131,255.11 | 124,749 | 5.42 |
| >110000 | all | 1,844,535,781 | 13,469 | 1,132,566 | 136,946.75 | 132,495 | 11.70 |
| >110000 | pass | 1,773,266,866 | 12,969 | 419,129 | 136,731.19 | 132,433 | 11.94 |
| >110000 | fail | 71,268,915 | 500 | 1,132,566 | 142,537.83 | 133,247 | 5.42 |
| >120000 | all | 1,358,976,282 | 9,235 | 1,132,566 | 147,154.98 | 141,952 | 11.67 |
| >120000 | pass | 1,307,213,229 | 8,905 | 419,129 | 146,795.42 | 141,876 | 11.90 |
| >120000 | fail | 51,763,053 | 330 | 1,132,566 | 156,857.74 | 144,987 | 5.45 |
| >130000 | all | 1,001,983,744 | 6,372 | 1,132,566 | 157,247.92 | 151,898 | 11.61 |
| >130000 | pass | 961,337,970 | 6,131 | 419,129 | 156,799.54 | 151,773 | 11.86 |
| >130000 | fail | 40,645,774 | 241 | 1,132,566 | 168,654.66 | 154,989 | 5.39 |
| >140000 | all | 725,839,707 | 4,320 | 1,132,566 | 168,018.45 | 162,007 | 11.58 |
| >140000 | pass | 696,332,742 | 4,162 | 419,129 | 167,307.24 | 161,700 | 11.82 |
| >140000 | fail | 29,506,965 | 158 | 1,132,566 | 186,752.94 | 168,926 | 5.27 |
| >150000 | all | 532,124,449 | 2,980 | 1,132,566 | 178,565.25 | 171,416 | 11.55 |
| >150000 | pass | 509,433,995 | 2,869 | 419,129 | 177,565.0 | 171,162 | 11.80 |
| >150000 | fail | 22,690,454 | 111 | 1,132,566 | 204,418.5 | 179,900 | 5.22 |
| >160000 | all | 389,075,705 | 2,055 | 1,132,566 | 189,331.24 | 181,178 | 11.50 |
| >160000 | pass | 371,348,145 | 1,976 | 419,129 | 187,929.22 | 180,971 | 11.75 |
| >160000 | fail | 17,727,560 | 79 | 1,132,566 | 224,399.49 | 199,865 | 5.14 |
| >170000 | all | 278,378,456 | 1,383 | 1,132,566 | 201,285.94 | 191,237 | 11.36 |
| >170000 | pass | 264,121,346 | 1,325 | 419,129 | 199,336.86 | 190,708 | 11.64 |
| >170000 | fail | 14,257,110 | 58 | 1,132,566 | 245,812.24 | 216,492 | 5.04 |
| >180000 | all | 201,295,618 | 942 | 1,132,566 | 213,689.62 | 203,116 | 11.24 |
| >180000 | pass | 190,017,460 | 901 | 419,129 | 210,896.18 | 202,285 | 11.54 |
| >180000 | fail | 11,278,158 | 41 | 1,132,566 | 275,077.02 | 243,487 | 4.84 |
| >190000 | all | 145,251,585 | 639 | 1,132,566 | 227,310.77 | 216,159 | 11.07 |
| >190000 | pass | 135,644,195 | 607 | 419,129 | 223,466.55 | 213,900 | 11.42 |
| >190000 | fail | 9,607,390 | 32 | 1,132,566 | 300,230.94 | 262,481 | 4.57 |
| >200000 | all | 111,796,168 | 467 | 1,132,566 | 239,392.22 | 227,194 | 10.95 |
| >200000 | pass | 102,976,106 | 439 | 419,129 | 234,569.72 | 225,046 | 11.36 |
| >200000 | fail | 8,820,062 | 28 | 1,132,566 | 315,002.21 | 263,733 | 4.51 |

Note: rank is the data length gradient,>0 is all data; Flag is the data type, all is the total sequencing data, pass is the valid sequencing data, and fail is the filtered data
