# Supplementary material for: High-quality assembly of the T2T genome for Isodon rubescens f. lushanensis reveals genomic structure variations between 2 typical forms of Isodon rubescens
Source: Gigascience. 2024 Oct 10;13:giae075. doi: 10.1093/gigascience/giae075 (PMC11466039; doi:10.1093/gigascience/giae075)
Supplement: giae075_Supplemental_Files [file giae075_supplemental_files.zip › Table_S10.docx]

| Number | Name of | Download address | Version |
| --- | --- | --- | --- |
| 1 | *I. rubescens* (Hemsl.) Hara | https://db.cngb.org/search/assembly/CNA0047362/ | CNP0002852 |
| 2 | *I. rubescens* f. *lushanensis* | This study | This study |
| 3 | *Ginkgo biloba* (maidenhair tree) | https://www.ncbi.nlm.nih.gov/data-hub/genome/GCA_024626585.1/ | GCA_024626585.1 |
| 4 | *Arabidopsis thaliana* (thale cress) | https://www.ncbi.nlm.nih.gov/data-hub/taxonomy/3702/ | GCF_000001735.4 |
| 5 | *Scutellaria baicalensis* (Baikal skullcap) | https://www.ncbi.nlm.nih.gov/data-hub/genome/GCA_005771605.1/ | GCA_005771605.1 |
| 6 | *Salvia miltiorrhiza* (Chinese salvia) | https://www.ncbi.nlm.nih.gov/data-hub/genome/GCA_016432925.1/ | GCA_016432925.1 |
| 7 | *Mentha longifolia* (horsemint) | https://www.ncbi.nlm.nih.gov/data-hub/genome/GCA_001642375.2/ | GCA_001642375.2 |
| 8 | *Salvia hispanica* | https://www.ncbi.nlm.nih.gov/labs/data-hub/genome/GCF_023119035.1/ | GCF_023119035.1 |
| 9 | *Perilla frutescens* (beefsteak-mint) | https://www.ncbi.nlm.nih.gov/data-hub/genome/GCA_026008535.1/ | GCA_026008535.1 |
| 10 | *Daucus carota* subsp. sativus | https://www.ncbi.nlm.nih.gov/data-hub/genome/GCF_001625215.1/ | GCF_001625215.1 |
| 11 | *Rosa chinensis* (China rose) | https://www.ncbi.nlm.nih.gov/data-hub/genome/GCF_002994745.2/ | GCF_002994745.2 |
| 12 | *Macleaya cordata* | https://www.ncbi.nlm.nih.gov/data-hub/genome/GCA_002174775.1/ | GCA_002174775.1 |
| 13 | *Zea mays* subsp. mays (maize) | https://www.ncbi.nlm.nih.gov/data-hub/genome/GCA_001644905.2/ | GCA_001644905.2 |
| 14 | *Oryza sativa* (Asian cultivated rice) | https://www.ncbi.nlm.nih.gov/data-hub/genome/GCA_001648735.1/ | GCA_001648735.1 |
| 15 | *Nymphaea colorata* (pocket water lily) | https://www.ncbi.nlm.nih.gov/data-hub/genome/GCF_008831285.2/ | GCF_008831285.2 |
