# Supplementary material for: High-quality assembly of the T2T genome for Isodon rubescens f. lushanensis reveals genomic structure variations between 2 typical forms of Isodon rubescens
Source: Gigascience. 2024 Oct 10;13:giae075. doi: 10.1093/gigascience/giae075 (PMC11466039; doi:10.1093/gigascience/giae075)
Supplement: giae075_Supplemental_Files [file giae075_supplemental_files.zip › Table_S2.docx]

| sampleid | HIFI reads | Total Bases(Gb) | HIFI Read length(bp) | HIFI reads length N50(bp) |
| --- | --- | --- | --- | --- |
| *I. rubescens* f.lushanensis | 1,604,661 | 30.55 | 19,037 | 19,277 |
