# Supplementary material for: High-quality assembly of the T2T genome for Isodon rubescens f. lushanensis reveals genomic structure variations between 2 typical forms of Isodon rubescens
Source: Gigascience. 2024 Oct 10;13:giae075. doi: 10.1093/gigascience/giae075 (PMC11466039; doi:10.1093/gigascience/giae075)
Supplement: giae075_Supplemental_Files [file giae075_supplemental_files.zip › Table_S3.docx]

| Sample | K-mer | K-mer Number | K-mer Depth | Genome Size (M) | Data Size(G) | X | Heterozygous Ratio(%) | Duplication Ratio(%) |
| --- | --- | --- | --- | --- | --- | --- | --- | --- |
| *I. rubescens* f.lushanensis | 19 | 98，556，357，522 | 282.355 | 349 | 114.57 | 328 | 1.7 | 83.43 |
