# Supplementary material for: High-quality assembly of the T2T genome for Isodon rubescens f. lushanensis reveals genomic structure variations between 2 typical forms of Isodon rubescens
Source: Gigascience. 2024 Oct 10;13:giae075. doi: 10.1093/gigascience/giae075 (PMC11466039; doi:10.1093/gigascience/giae075)
Supplement: giae075_Supplemental_Files [file giae075_supplemental_files.zip › Table_S5.docx]

|  |  | Assembly | | Annotation | |
| --- | --- | --- | --- | --- | --- |
| Forms | BUSCOs | Proteins | Percentage (%) | Proteins | Percentage (%) |
| I. rubescens-JY | Complete BUSCOs | 1522 | 94.3 | 1518 | 94 |
| I. rubescens-LS |  | 1594 | 98.7 | 1586 | 98.2 |
| I. rubescens-JY | Complete Single-Copy BUSCOs | 1420 | 88 | 1345 | 83.3 |
| I. rubescens-LS |  | 1524 | 94.4 | 1526 | 94.5 |
| I. rubescens-JY | Complete Duplicated BUSCOs | 102 | 6.3 | 173 | 10.7 |
| I. rubescens-LS |  | 70 | 4.3 | 60 | 3.7 |
| I. rubescens-JY | Fragmented BUSCOs | 30 | 1.9 | 23 | 1.4 |
| I. rubescens-LS |  | 3 | 0.2 | 9 | 0.6 |
| I. rubescens-JY | Missing BUSCOs | 62 | 3.8 | 73 | 4.6 |
| I. rubescens-LS |  | 17 | 1.1 | 19 | 1.2 |
| I. rubescens-JY | Total BUSCO groups searched | 1614 | 100 | 1614 | 100 |
| I. rubescens-LS |  | 1614 | 100 | 1614 | 100 |
