# Supplementary material for: High-quality assembly of the T2T genome for Isodon rubescens f. lushanensis reveals genomic structure variations between 2 typical forms of Isodon rubescens
Source: Gigascience. 2024 Oct 10;13:giae075. doi: 10.1093/gigascience/giae075 (PMC11466039; doi:10.1093/gigascience/giae075)
Supplement: giae075_Supplemental_Files [file giae075_supplemental_files.zip › Table_S6.docx]

| Chromosome | Length(bp) | contig number |
| --- | --- | --- |
| chr1 | 43,550,678 | 1 |
| chr2 | 42,809,313 | 1 |
| chr3 | 33,847,246 | 1 |
| chr4 | 30,327,435 | 1 |
| chr5 | 30,570,578 | 1 |
| chr6 | 28,219,675 | 1 |
| chr7 | 28,843,235 | 1 |
| chr8 | 29,480,909 | 1 |
| chr9 | 26,375,046 | 1 |
| chr10 | 27,251,472 | 1 |
| chr11 | 28,179,645 | 1 |
| chr12 | 27,047,962 | 1 |
