# Supplementary material for: High-quality assembly of the T2T genome for Isodon rubescens f. lushanensis reveals genomic structure variations between 2 typical forms of Isodon rubescens
Source: Gigascience. 2024 Oct 10;13:giae075. doi: 10.1093/gigascience/giae075 (PMC11466039; doi:10.1093/gigascience/giae075)
Supplement: giae075_Supplemental_Files [file giae075_supplemental_files.zip › Table_S7.docx]

|  | TE protiens | | De novo + repbase | | Combined TEs | |
| --- | --- | --- | --- | --- | --- | --- |
| Type | Length (Bp) | % in genome | Length (Bp) | % in genome | Length (Bp) | % in genome |
| DNA | 2,714,140 | 0.72 | 16,530,938 | 4.40 | 16,959,544 | 4.51 |
| LINE | 1,997,755 | 0.53 | 4,439,388 | 1.18 | 4,623,747 | 1.23 |
| SINE | 0 | 0.00 | 476,453 | 0.13 | 476,453 | 0.13 |
| LTR | 30,888,871 | 8.22 | 107,856,111 | 28.69 | 108,937,680 | 28.98 |
| LTR-Gypsy | 17,221,494 | 4.58 | 64,473,009 | 17.15 | 65,209,736 | 17.35 |
| LTR-Copia | 12,722,309 | 3.38 | 28,050,350 | 7.46 | 28,450,653 | 7.57 |
| Satellite | 0 | 0.00 | 529,057 | 0.14 | 529,057 | 0.14 |
| Simple_repeat | 0 | 0.00 | 48,585 | 0.01 | 48,585 | 0.01 |
| Other | 0 | 0.00 | 2,451 | 0.00 | 2,451 | 0.00 |
| Unknown | 8,496 | 0.00 | 102,821,739 | 27.35 | 102,830,199 | 27.35 |
| Total | 35,606,289 | 9.47 | 214,676,708 | 57.11 | 219,812,669 | 58.47 |
