# Supplementary material for: High-quality assembly of the T2T genome for Isodon rubescens f. lushanensis reveals genomic structure variations between 2 typical forms of Isodon rubescens
Source: Gigascience. 2024 Oct 10;13:giae075. doi: 10.1093/gigascience/giae075 (PMC11466039; doi:10.1093/gigascience/giae075)
Supplement: giae075_Supplemental_Files [file giae075_supplemental_files.zip › Table_S8.docx]

| Item | Number（I. rubescens-JY） | Number（I. rubescens-LS） |
| --- | --- | --- |
| the total number of gene | 30,789 | 34,865 |
| the average of mRNA_length (bp) | 3,711.47 | 3,787.28 |
| the average cds_length of per gene (bp) | 1,200.50 | 1,163.68 |
| the average exon_number of per gene | 4.84 | 4.47 |
| the average of exon_length (bp) | 290.7 | 405.77 |
| the average of intron_length (bp) | 594.05 | 562.75 |
| the total number of exon | 149,143 | 155,923 |
| the total number of intron | 118,354 | 121,058 |
| the total intron length (bp) | 70,308,119 | 68,125,450 |
