# Supplementary material for: High-quality assembly of the T2T genome for Isodon rubescens f. lushanensis reveals genomic structure variations between 2 typical forms of Isodon rubescens
Source: Gigascience. 2024 Oct 10;13:giae075. doi: 10.1093/gigascience/giae075 (PMC11466039; doi:10.1093/gigascience/giae075)
Supplement: giae075_Supplemental_Files [file giae075_supplemental_files.zip › Table_S9.docx]

| Item | Count | Percentage |
| --- | --- | --- |
| All | 34,865 | 100.00% |
| Annotation | 32,885 | 94.32% |
| KEGG | 7,866 | 22.56% |
| Pathway | 6,182 | 17.73% |
| Nr | 31,081 | 89.15% |
| Uniprot | 30,722 | 88.12% |
| GO | 22,734 | 65.21% |
| KO | 1,116 | 3.20% |
| Pfam | 22,505 | 64.55% |
| Interpro | 32,098 | 92.06% |
