# Supplementary figures and images for: High-quality assembly of the T2T genome for Isodon rubescens f. lushanensis reveals genomic structure variations between 2 typical forms of Isodon rubescens
Source: Gigascience. 2024 Oct 10;13:giae075. doi: 10.1093/gigascience/giae075 (PMC11466039; doi:10.1093/gigascience/giae075)

HiFi Read Length Distribution

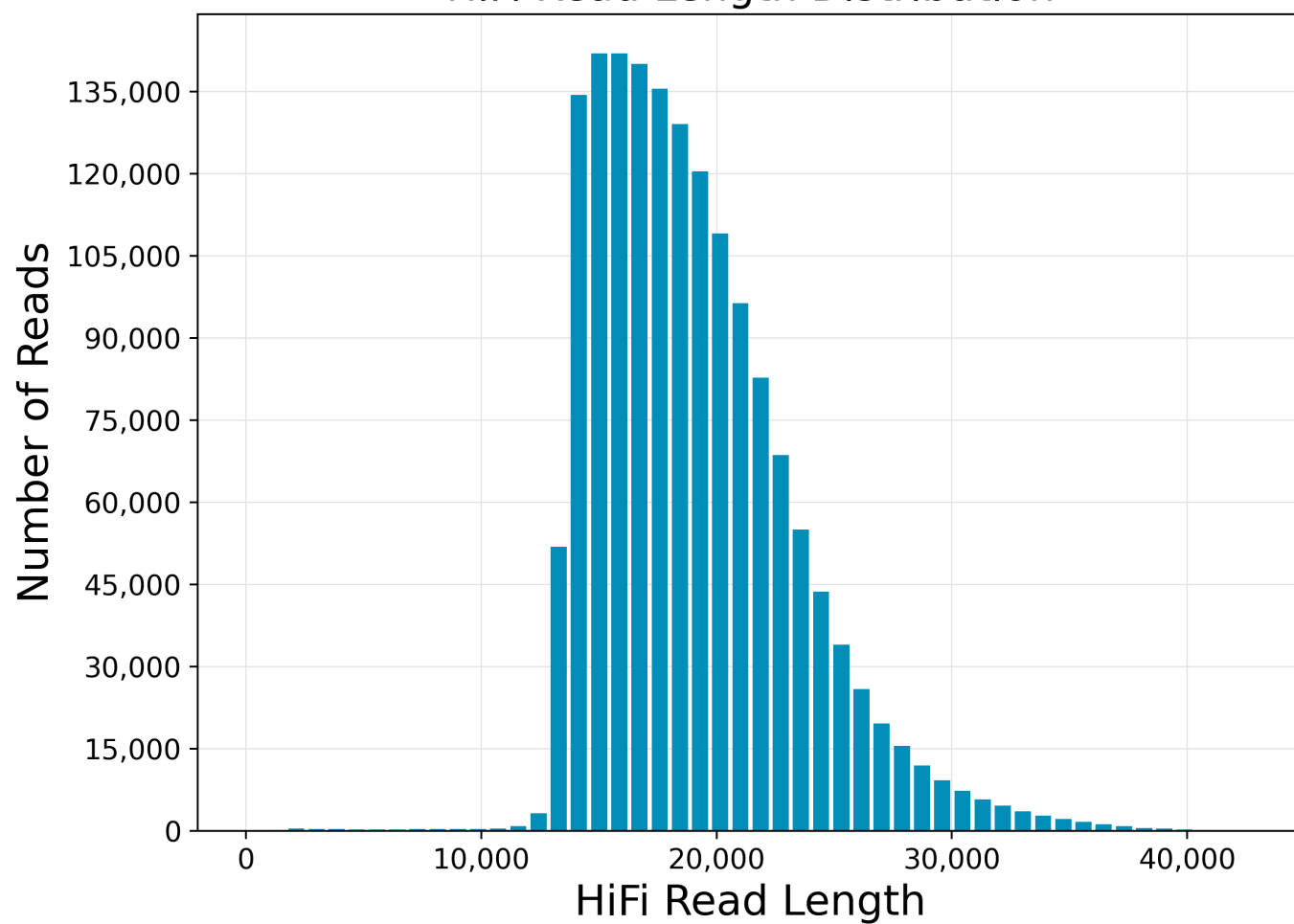

Supplement: giae075_Supplemental_Files [file giae075_supplemental_files.zip › Fig_S1.pdf]

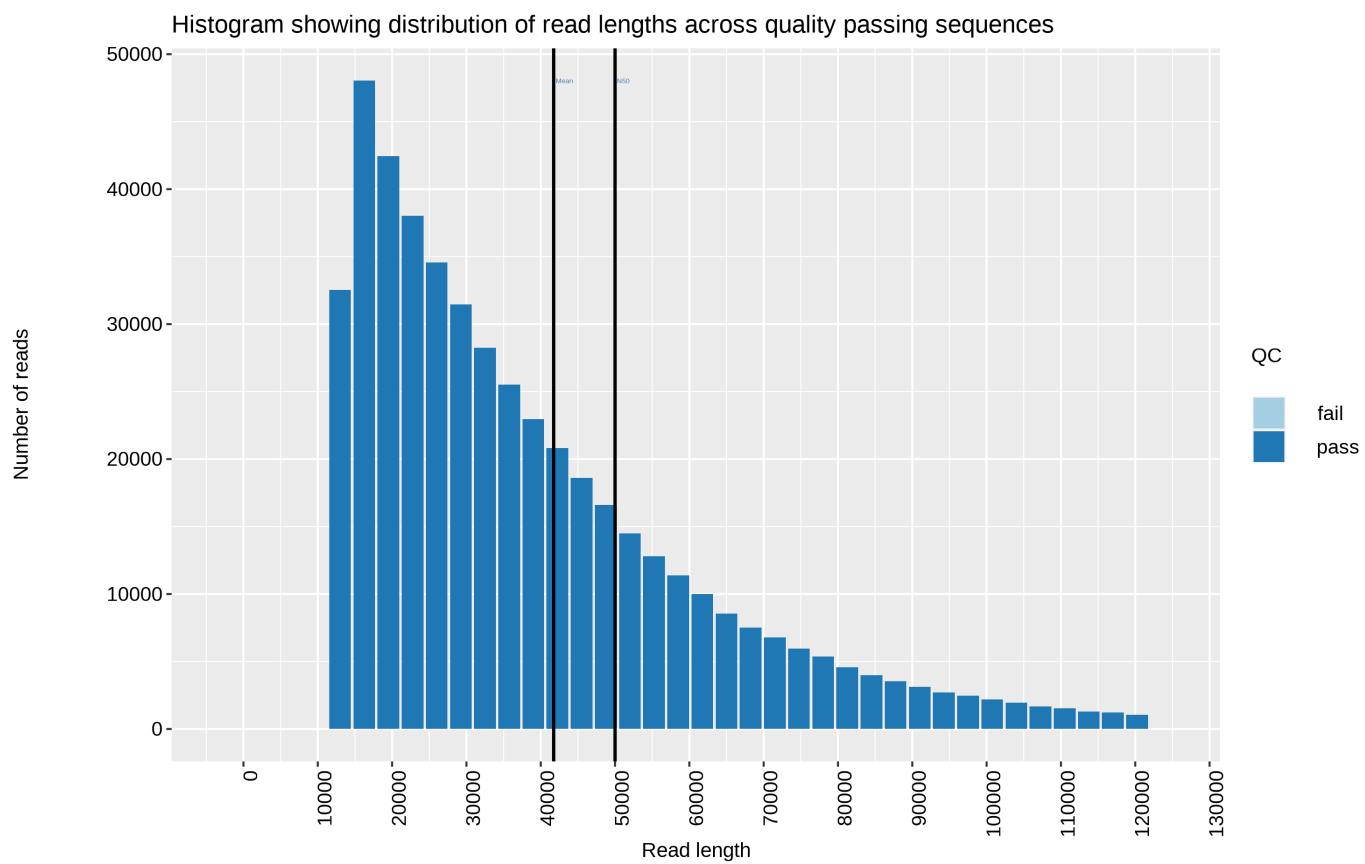

Supplement: giae075_Supplemental_Files [file giae075_supplemental_files.zip › Fig_S2.pdf]

## Kmer Depth-Frequency Distribution

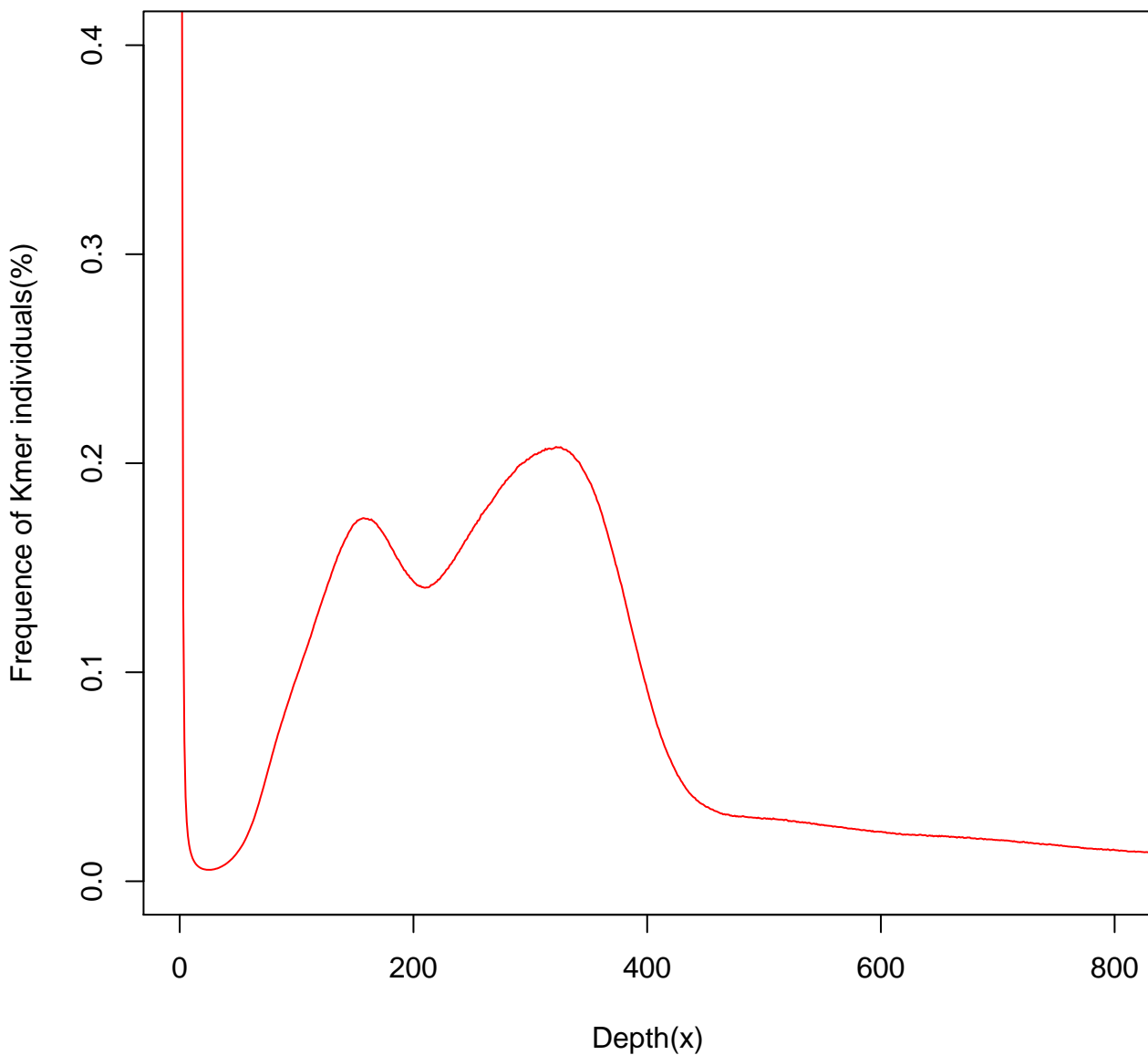

Supplement: giae075_Supplemental_Files [file giae075_supplemental_files.zip › Fig_S3.pdf]

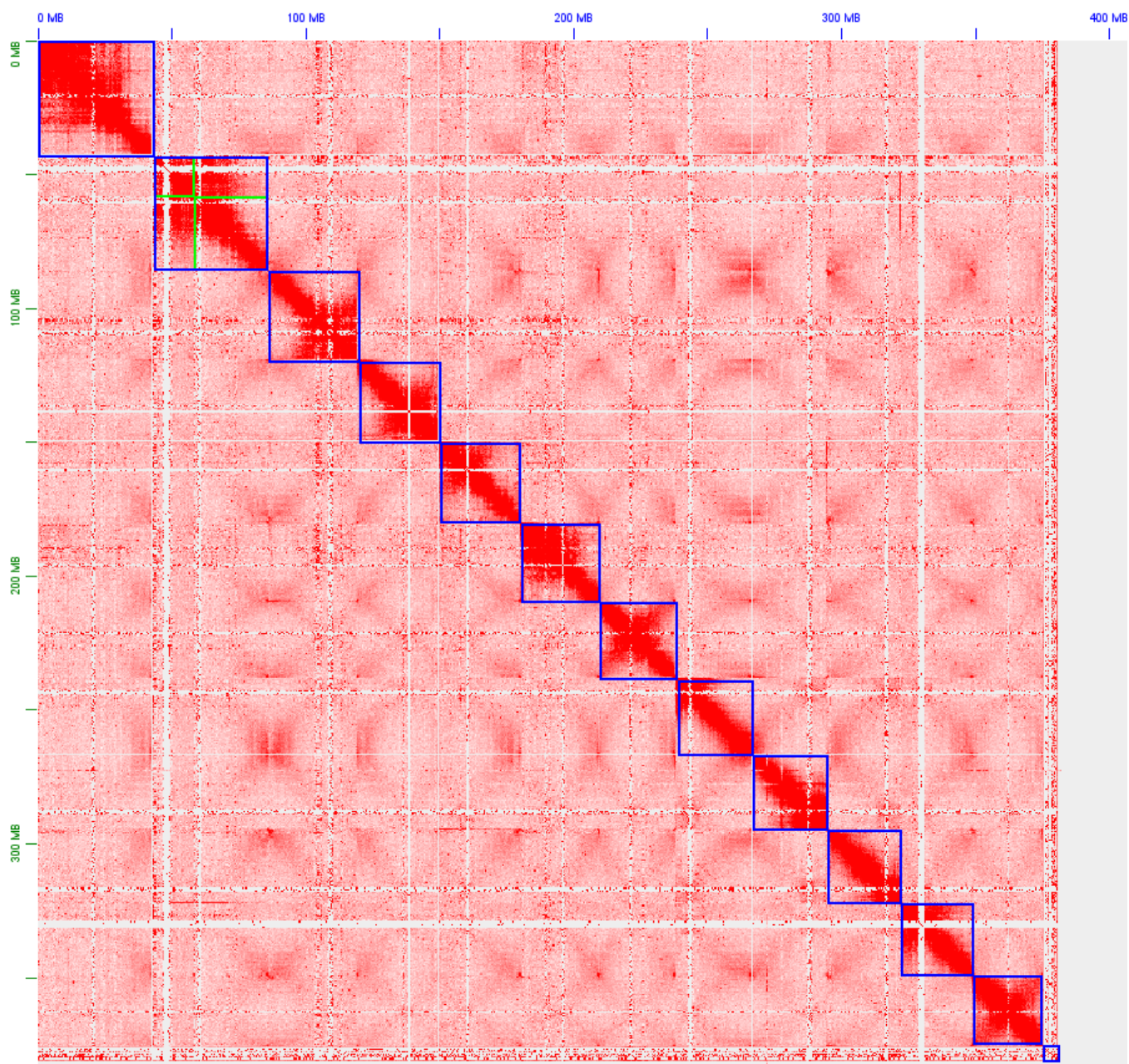

Supplement: giae075_Supplemental_Files [file giae075_supplemental_files.zip › Fig_S4.pdf]

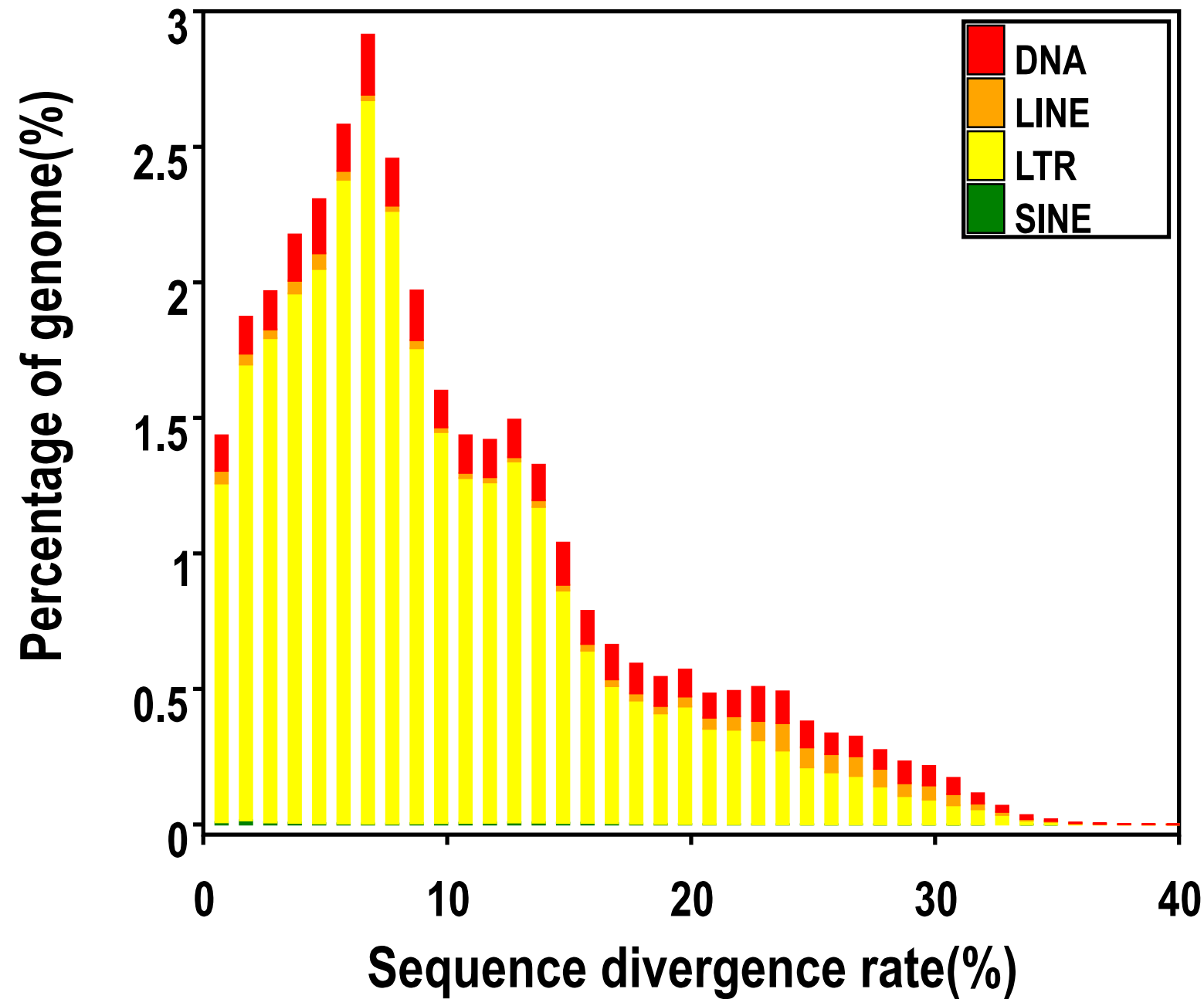

Supplement: giae075_Supplemental_Files [file giae075_supplemental_files.zip › Fig_S5.pdf]

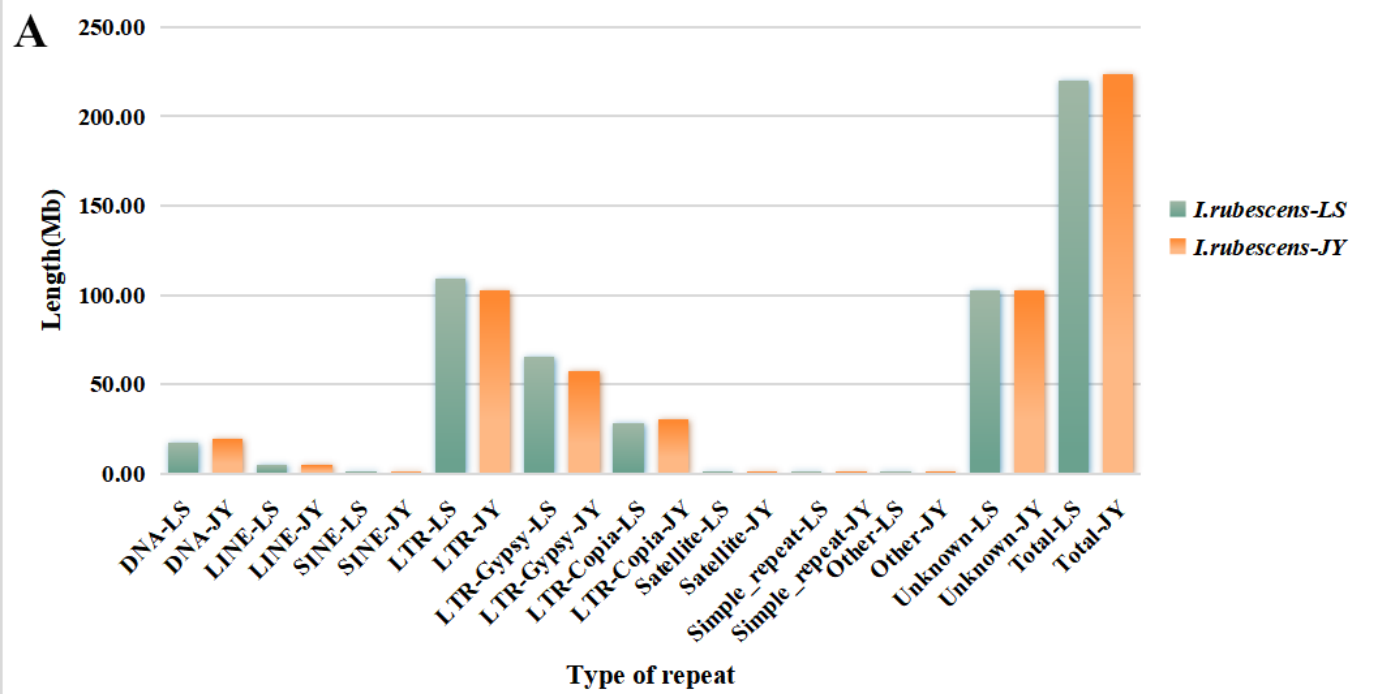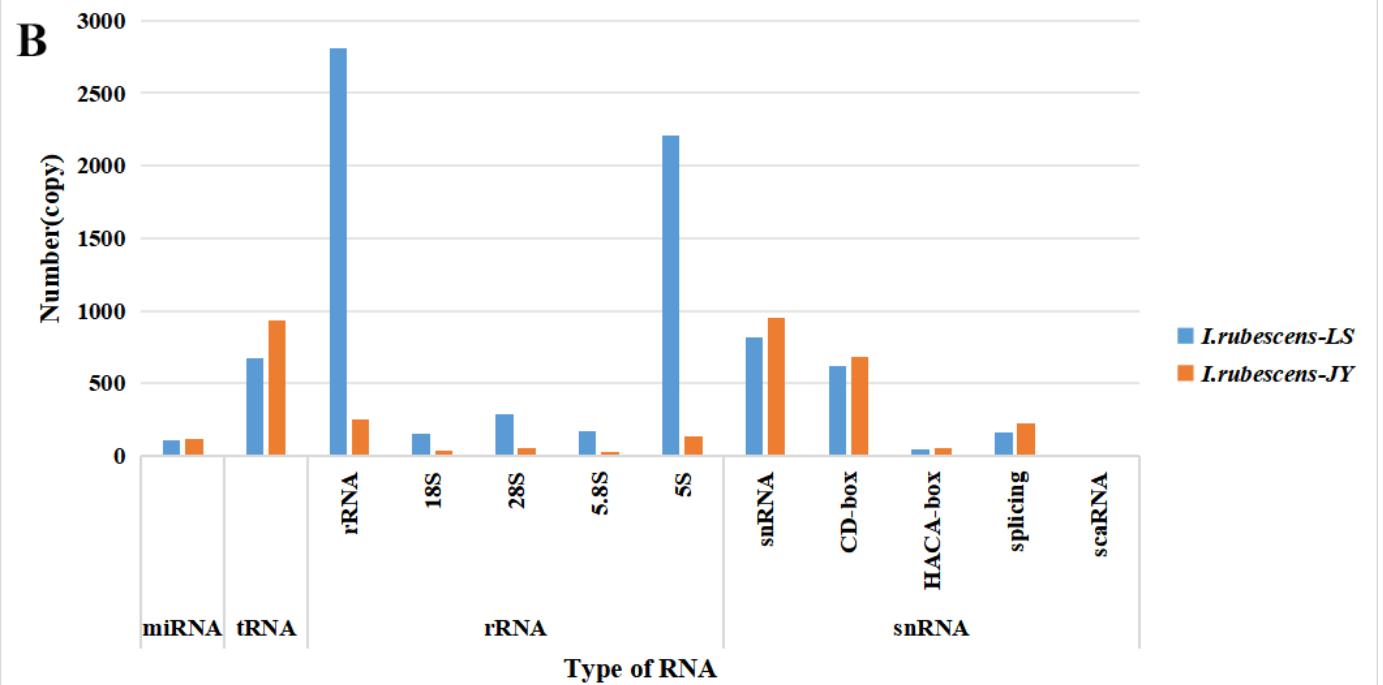

Supplement: giae075_Supplemental_Files [file giae075_supplemental_files.zip › Fig_S6.pdf]

A

*I. rubescens*-LS

*I. rubescens*-JY

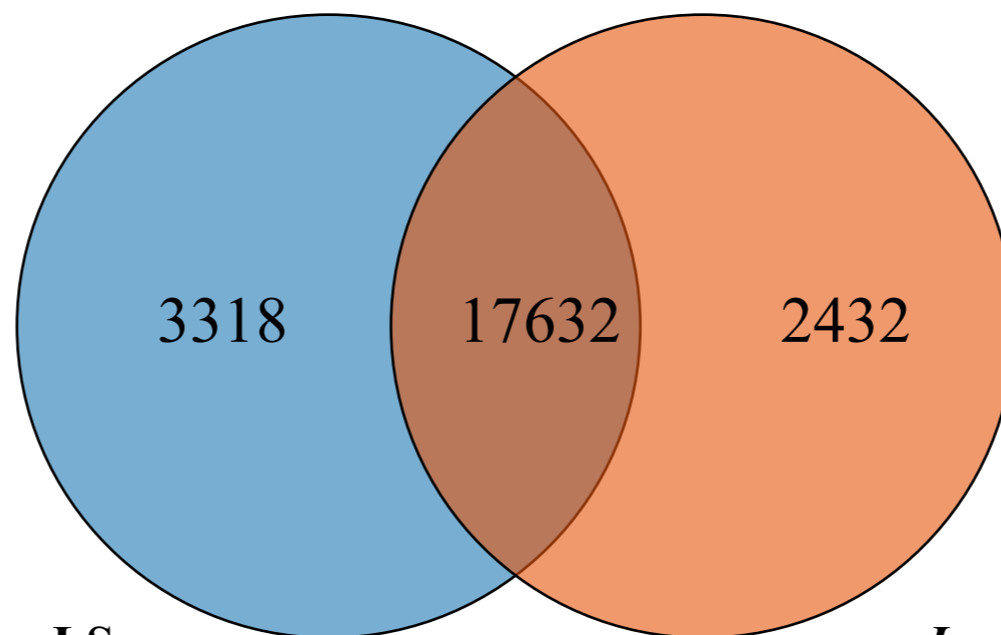

B

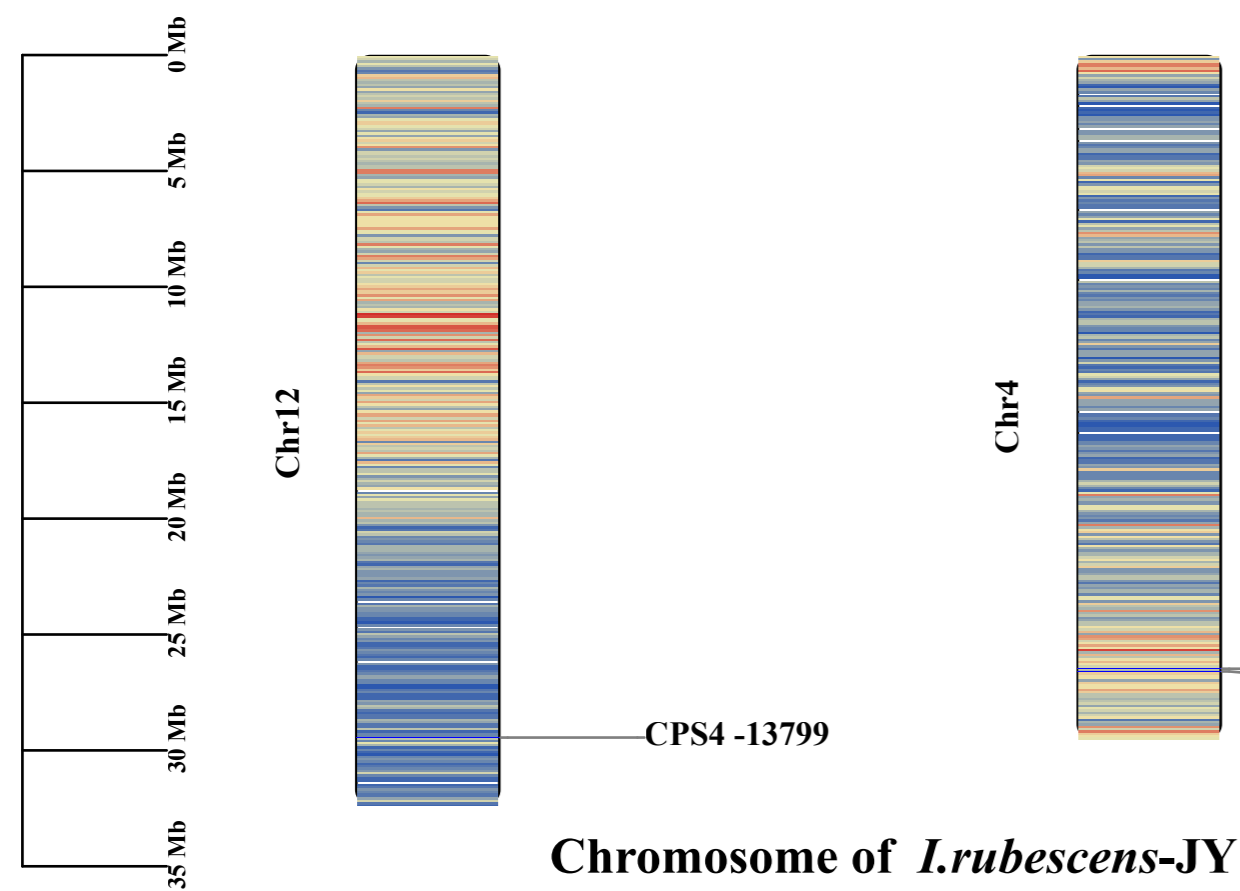

C

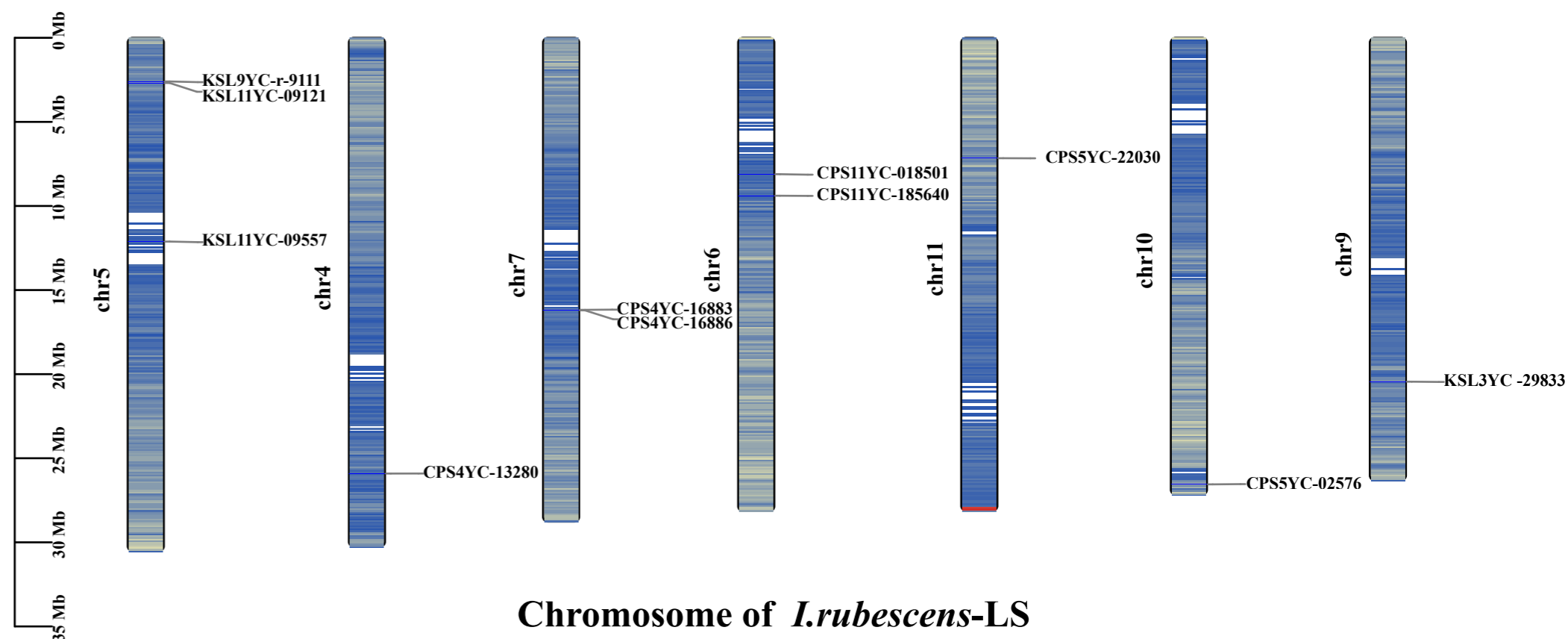

Supplement: giae075_Supplemental_Files [file giae075_supplemental_files.zip › Fig_S7.pdf]

A

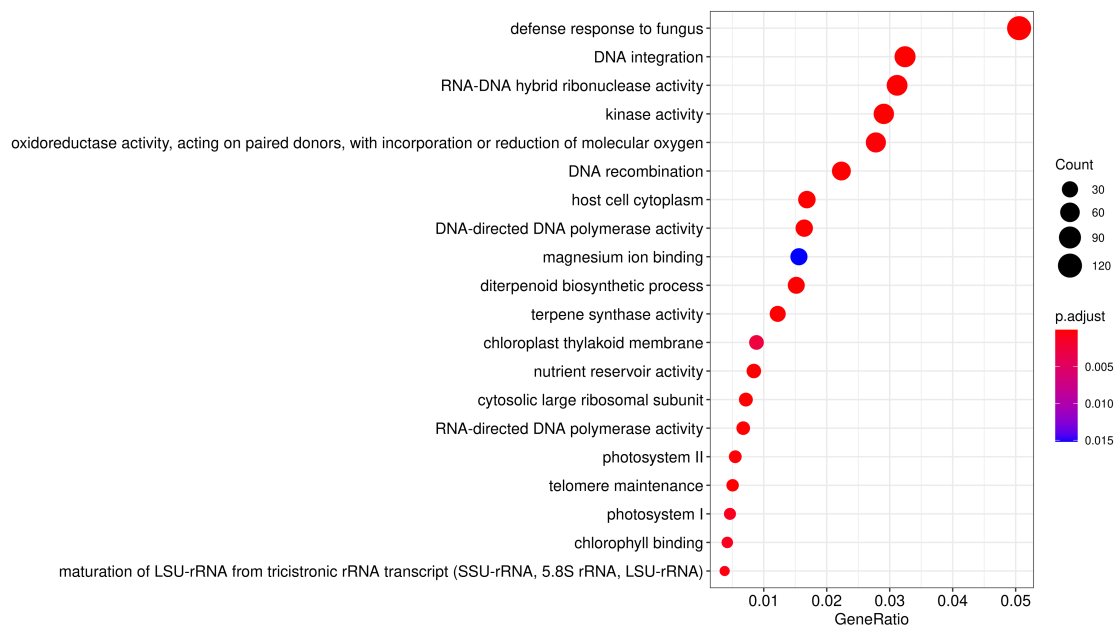

B

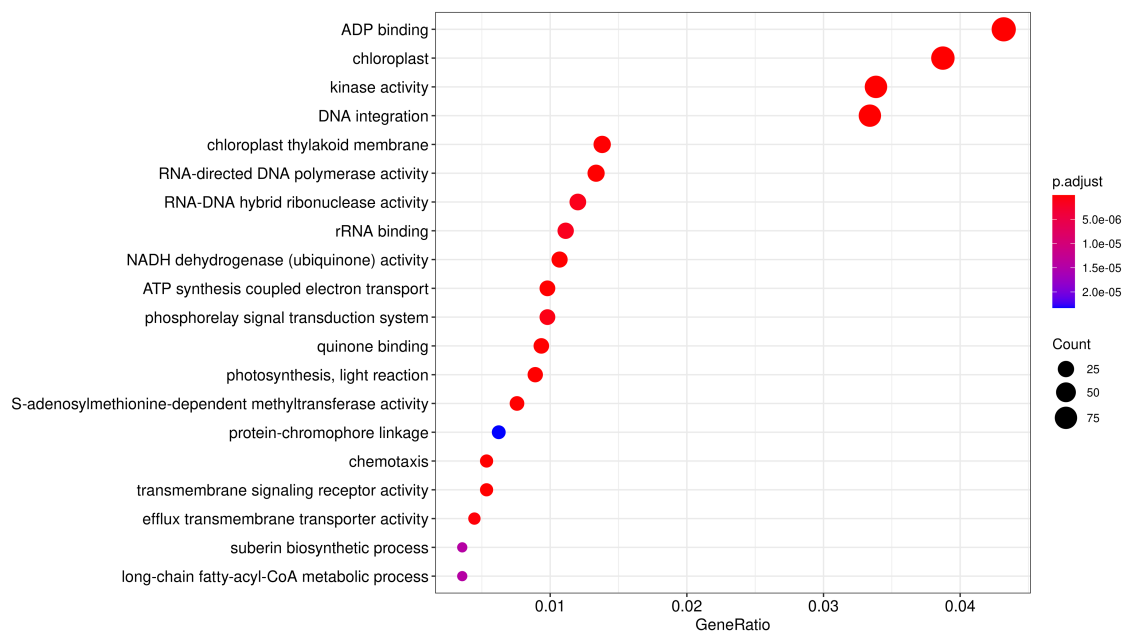

Supplement: giae075_Supplemental_Files [file giae075_supplemental_files.zip › Fig_S8.pdf]

A

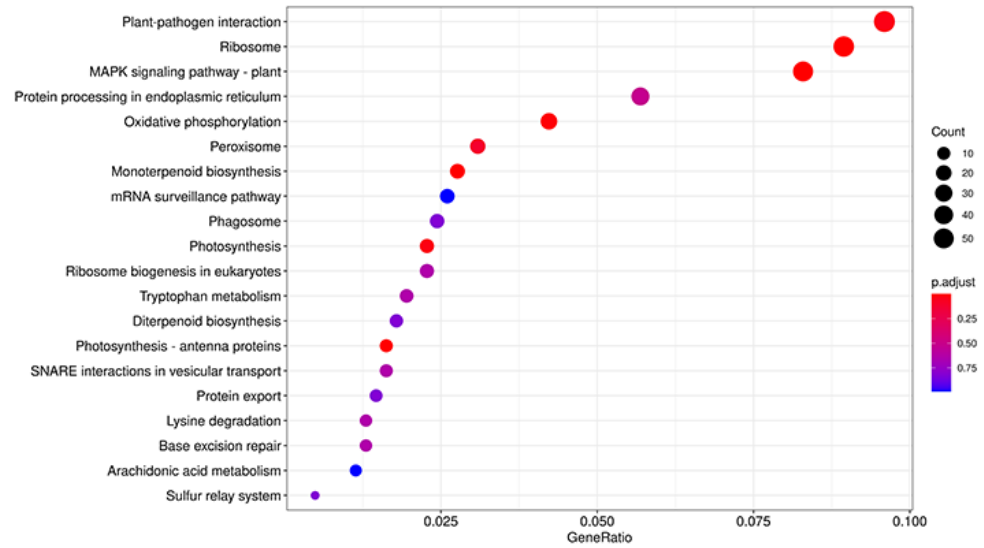

B

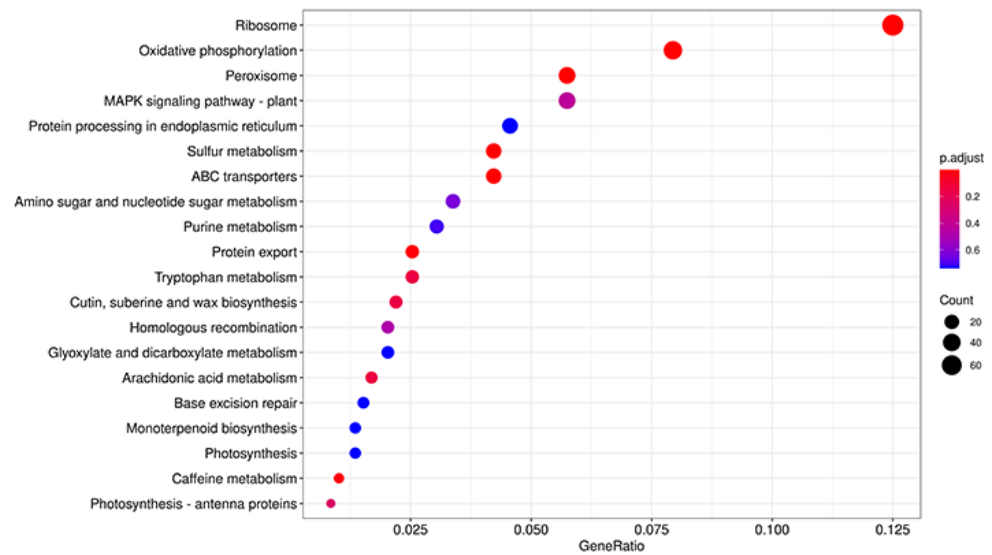

Supplement: giae075_Supplemental_Files [file giae075_supplemental_files.zip › Fig_S9.pdf]
