# Supplementary material for: High-quality assembly of the T2T genome for Isodon rubescens f. lushanensis reveals genomic structure variations between 2 typical forms of Isodon rubescens
Source: Gigascience. 2024 Oct 10;13:giae075. doi: 10.1093/gigascience/giae075 (PMC11466039; doi:10.1093/gigascience/giae075)
Supplement: giae075_GIGA-D-24-00177_Original_Submission [file giae075_giga-d-24-00177_original_submission.pdf]

GigaScience

High-quality assembly of the T2T genome for Isodon rubescens f. lushanensis reveals key enzymes involved in structural variation of diterpenoid biosynthetic Pathways between two typical forms of Isodon rubescens

--Manuscript Draft--

**Manuscript Number:**	GIGA-D-24-00177
**Full Title:**	High-quality assembly of the T2T genome for Isodon rubescens f. lushanensis reveals key enzymes involved in structural variation of diterpenoid biosynthetic Pathways between two typical forms of Isodon rubescens
**Article Type:**	Data Note
**Funding Information:**	Project of Science and Technology Research and Development of Henan Province (Superior discipline Cultivation) (Grant No.232301420078) — professor Suiqing Chen<br>Innovative Research Group Project of the National Natural Science Foundation of China (Frant No. 81173486) — professor Suiqing Chen<br>Chinese Herbal Medicine Industry Technology System of Henan Province (Grant No.14 [2018]) — professor Suiqing Chen
**Abstract:**	Background: Rabdosiae rubescentis herba (Isodon rubescens) is widely used as a folk medicine to treat esophageal cancer and sore throat in China. Its germplasm resources are abundant in China, with I. rubescens (Hemsl.) Hara and I. rubescens f. lushanensis as two typical forms. I. rubescens (Hemsl.) Hara is featured by biosynthesis of the diterpenoid oridonin with strong anti-cancer activity, while I. rubescens f. lushanensis produces another diterpenoid with anti-cancer activity, lushanrubescensin. However, the biosynthetic pathways of both still need to be fully understood. In particular, little is known about the genetic background of I. rubescens f. lushanensis. Findings: We used Pacific Biosciences (PacBio) single-molecule real-time and Nanopore Ultra-long sequencing platforms respectively, and finally obtained 139.07 Gb of high-quality data, with a sequencing depth of about 328X, We finally obtained a high-quality reference genome for I. rubescens f. lushanensis, with a genome size of 349 Mb and a contig N50 of 28.8 Mb. The heterozygosity of the genome is 1.7% and the repeatability is 83.43%. 34,865 protein-coding genes were predicted. The divergence time between I. rubescens (Hemsl.) Hara and I. rubescens f. lushanensis was estimated at 8.7 million years ago (Mya). Moreover,We found that most of the variant or unique genes in the diterpenoid synthesis pathways of I. rubescens f. lushanensis and I. rubescens (Hemsl.) Hara were enriched in diterpene synthases. Conclusions:We provide the first genome sequence and gene annotation for the I. rubescens f. lushanensis, which provides molecular evidence for understanding the chemotypic differences of I. rubescens.
**Corresponding Author:**	Hao Yang<br>Henan University of Chinese Medicine<br>Zhengzhou, CHINA
**Corresponding Author Secondary Information:**	
**Corresponding Author's Institution:**	Henan University of Chinese Medicine
**Corresponding Author's Secondary Institution:**	
**First Author:**	Hao Yang
**First Author Secondary Information:**	
**Order of Authors:**	Hao Yang<br>Conglong Lian

Powered by Editorial Manager® and ProduXion Manager® from Aries Systems Corporation

	Jinlu Lliu
	Hongwei Yu
	Le Zhao
	Ni He
	Xiuyu Liu
	Shujuan Xue
	Xiaoya Sun
	Liping Zhang
	Lili Wang
	Jingfan Yang
	Yu Fu
	Rui Ma
	Bao Zhang
	Lidan Ye
	Suiqing Chen
**Order of Authors Secondary Information:**	
**Additional Information:**	
**Question**	**Response**
Are you submitting this manuscript to a special series or article collection?	No
**Experimental design and statistics**<br><br>Full details of the experimental design and statistical methods used should be given in the Methods section, as detailed in our Minimum Standards Reporting Checklist. Information essential to interpreting the data presented should be made available in the figure legends.<br><br>Have you included all the information requested in your manuscript?	Yes
**Resources**<br><br>A description of all resources used, including antibodies, cell lines, animals and software tools, with enough information to allow them to be uniquely identified, should be included in the Methods section. Authors are strongly encouraged to cite Research Resource	Yes

Identifiers (RRIDs) for antibodies, model organisms and tools, where possible.<br><br>Have you included the information requested as detailed in our Minimum Standards Reporting Checklist?	
**Availability of data and materials**<br><br>All datasets and code on which the conclusions of the paper rely must be either included in your submission or deposited in publicly available repositories (where available and ethically appropriate), referencing such data using a unique identifier in the references and in the “Availability of Data and Materials” section of your manuscript.<br><br>Have you have met the above requirement as detailed in our Minimum Standards Reporting Checklist?	Yes

Manuscript Click here to access/download;Manuscript;Manuscript.docx

High-quality assembly of the T2T genome for *Isodon rubescens* f. lushanensis reveals key enzymes involved in structural variation of diterpenoid biosynthetic Pathways between two typical forms of *Isodon rubescens*

Hao Yang[1,3,4†], Conglong Lian[1,3,4†], Jinlu Liu[1†], Hongwei Yu[2], Le Zhao[1], Ni He[2], Xiuyu Liu[1], Shujuan Xue[1,3,4], Xiaoya Sun[1,3,4], Liping Zhang[1], Lili Wang[1], Jingfan Yang[1], Yu Fu[1], Rui Ma[1], Bao Zhang[1], Lidan Ye[2,*] and Suiqing Chen[1,3,4*]

1 College of Pharmacy, Henan Key Laboratory of Chinese Medicine Resources and Chemistry, Henan University of Chinese Medicine, Henan, PR China.

2 Institute of Bioengineering, College of Chemical and Biological Engineering, Zhejiang University, Zhejiang, PR China.

3 Collaborative Innovation Center of Research and Development on the Whole Industry Chain of Yu-Yao, Henan, PR China.

4 Co-Construction Collaborative Innovation Centre for Chinese Medicine and Respiratory Diseases by Henan & Education Ministry of China, Henan, PR China.

*Correspondence: Lidan Ye, Institute of Bioengineering, College of Chemical and Biological Engineering, Zhejiang University, Zhejiang, 310058, China; E-mail:yelidan@zju.edu.cn; Suiqing Chen, College of Pharmacy, Henan Key Laboratory of Chinese Medicine Resources and Chemistry, Henan University of Chinese Medicine, Henan, 450046, China; E-mail:chsq@hactcm.edu.cn

†These authors contributed equally

Abstract

Background: Rabdosiae rubescentis herba (*Isodon rubescens*) is widely used as a folk medicine to treat esophageal cancer and sore throat in China. Its germplasm resources are abundant in China, with *I. rubescens* (Hemsl.) Hara and *I. rubescens* f. *lushanensis* as two typical forms. *I. rubescens* (Hemsl.) Hara is featured by biosynthesis of the diterpenoid oridonin with strong anti-cancer activity, while *I. rubescens* f. *lushanensis*

produces another diterpenoid with anti-cancer activity, lushanrubescensin. However, the biosynthetic pathways of both still need to be fully understood. In particular, little is known about the genetic background of *I. rubescens* f. *lushanensis.* **Findings:** We used Pacific Biosciences (PacBio) single-molecule real-time and Nanopore Ultra-long sequencing platforms respectively, and finally obtained 139.07 Gb of high-quality data, with a sequencing depth of about 328X, We finally obtained a high-quality reference genome for *I. rubescens* f. *lushanensis*, with a genome size of 349 Mb and a contig N50 of 28.8 Mb. The heterozygosity of the genome is 1.7% and the repeatability is 83.43%. 34,865 protein-coding genes were predicted. The divergence time between *I. rubescens* (Hemsl.) Hara and *I. rubescens* f. lushanensis was estimated at 8.7 million years ago (Mya). Moreover,We found that most of the variant or unique genes in the diterpenoid synthesis pathways of *I. rubescens* f. *lushanensis* and *I. rubescens* (Hemsl.) Hara were enriched in diterpene synthases. **Conclusions:**We provide the first genome sequence and gene annotation for the *I. rubescens* f. lushanensis, which provides molecular evidence for understanding the chemotypic differences of *I. rubescens.*

Key words: telomere-to-telomere genome sequencing; *I. rubescens* f. *lushanensis*; plant diterpenoid biosynthesis; diterpene synthase; evolution

Data Description

Background information

Isodon is an important genus in Lamiaceae, a large family rich in medicinal plant resources and widely distributed worldwide. There are about 150 species in the world, and more than 90 species and 25 varieties in China, of which 30 species are used for folk medicine. Nearly 300 new diterpenoids have been isolated from more than 40 species of *Isodon* [1]. Most *Isodon* are rich in ent-kaurane diterpenoids, featured by an α-methylene cyclopentanone structure and strong anti-cancer activity [2]. Among them, oridonin is one of the representative components, which is the unique medicinal ingredient for *Isodon rubescens*, reported to have good anti-cancer and anti-tumor activities, few side effects, and the capability to reduce the adverse reactions caused

by chemotherapy drugs. The domestic reference quotation for this product is 180,000 to 220,000 CNY/kg, and the quotation for the international market is even higher [3].

During the survey of *I. rubescens* germplasm resources, *I. rubescens* was divided into two forms, namely, *I. rubescens* (Hemsl.) Hara (original variant) and *I. rubescens* f.lushanensis (new variant), which differs in the production of diterpenoids. Especially, *I. rubescens* (Hemsl.) Hara produces oridonin while *I. rubescens* f. *lushanensis* produces lushanrubescensin with similar tetracyclic backbone and different modifications [4]. This kind of chemotypic variation is ubiquitous in plants and is an evolutionary choice for species to adapt to their living environment in the long term. Environment and genotype factors that affect the formation of plant secondary metabolites may combinatorically lead to the generation of different chemotypic variations [5]. The result of gene fragment sequence comparison shows low proportion (0.3%) of base deletions in the ITS (internal transcribed space) sequences of the two chemotypic variations of *I. rubescen*, and suggests the most relevant genes for the formation of chemotypes are the genes that regulate the synthesis and regulation of secondary metabolites [6]. Therefore, it is of great significance to get a full scan of the genomic information for these two forms of *I. rubescens*, with focus on the diterpenoid synthetic pathway genes, so as to get insight into the mechanism of chemotype formation.

A recent study on the chromosome-level genome sequencing of *I. rubescens* (Hemsl.) Hara found multiple *I. rubescens* CYP706 family proteins that can introduce hydroxyl group at different positions on the kaurane skeleton structure, getting clue on the P450s involved in oridonin formation [7]. This known genomic information and partially revealed oridonin synthesis pathway of *I. rubescens* (Hemsl.) Hara in the above work could serve as a reference for the comparative research on the genomic difference of the two forms of *I. rubescens*. By comparing the genes involved in GGPP cyclization and hydroxyl modification between the two forms, it is possible to find out the unique genes responsible for synthesis of oridonin and lushanrubescensin, respectively. In this study, T2T (Telomere-to-Telomere) genome sequencing was conducted for *I. rubescens* f. *lushanensis* to obtain a high-quality genome for

comparative analysis with the reported genome of *I. rubescens* (Hemsl.) Hara. Discovery of new diterpenoid synthases and P450s involved in diterpenoid synthesis of *I. rubescens* in this work by comparative genomic analysis would provide insight into the chemotypic variation of *I. rubescens* and the synthesis pathway of *I. rubescens* diterpenoids.

Plant material

Isodon rubescens f. lushanensis was sourced from Lushan County, Henan Province, China (33°72′N, 112°29′E). It was independently identified as *I. rubescens* f.lushanensis by Professor Suiqing Chen of Henan University of Chinese Medicine. The collected samples are preserved in the Molecular Pharmacology Laboratory of Henan University of Chinese Medicine (Room number:BM628). When we use d materials, fresh leaves were harvested, rinsed, and the surface moisture was removed. After being individually wrapped in tin foil, they were immediately flash-frozen in liquid nitrogen for 30 minutes and stored in a -80°C refrigerator. The samples were then dispatched to Wuhan Beina Technology Co., Ltd. for sequencing.

PacBio HiFi sequencing

High-quality genomic DNA was extracted from leaves of *I. rubescens* f.lushanensis following CTAB (Cetyltrimethylammonium Bromide) DNA extraction protocol [8]. 0.75% agarose gel electrophoresis was used to detect the size of DNA fragments and the degree of DNA degradation; NanoDrop One spectrophotometer (Thermo Fisher Scientific) was used to detect DNA purity; Qubit 3.0 fluorescence analyzer (Life Technologies) was used to detect DNA concentration and accurately quantify DNA. Subsequently, a PCR-free SMRT bell library was constructed, and the library template and enzyme complex were transferred to the nanopores of the Sequel II sequencer (PacBio) for sequencing. The data was read using the official PacBio software (SMRTlink v8.0) for quality control and statistical analysis of the output data, The raw data was filtered using CCS v6.0.0 (CCS, RRID:SCR_021174) to obtain valid data for subsequent analysis. A total of 1,604,661 PacBio post-filtered reads were

generated. This produced 30.55Gb of single-molecule sequencing data, with an average read length of 19,037 bp (Supplementary Fig. S1 and Table S1)

Nanopore ONT Ultra-long

ONT sequencing was a single-molecule real-time sequencing technology that relied on nanopore electrical signals. Under the influence of motor proteins, DNA double strands bind to octamer nanopore proteins anchored to the biofilm and unwind [9]. Owing to a potential difference across the biofilm, the unwound single strands of DNA moved through the nanopores at a set speed. Due to their distinct chemical properties, as different bases passed through the nanopores, they triggered variations in electrical signals [10]. The detection and interpretation of these signal variations allowed for real-time sequencing [11].Initially, high-quality DNA samples were extracted and tested for purity, concentration, and integrity. The purified product was then linked using sequencing adapters from the assay kit (SQK-LSK109). Once the library was constructed, the DNA library was loaded into the Flow cell and transferred to the Oxford Nanopore sequencer (PromethION) for real-time single-molecule sequencing. The raw data was filtered using Filtlong v0.2.4 (Filtlong, RRID:SCR_024020) to obtain valid data for subsequent analysis. A total of 465,394 reads were generated. This produced 19.37 Gb of raw sequencing data, with an average cleaned read length of 41,620.42 bp (Supplementary Fig. S2 and Table S2)

Estimation of genome size, heterozygosity, and repeat content

This experiment employed the K-mer-based analysis method to estimate genome size and heterozygosity rates. The genome size was assessed using GCE v1.0.0 (GCE, RRID:SCR_017332) [12], Frequency-depth distribution was obtained by Jellyfish v2.2.10 (Jellyfish, RRID:SCR 005491) [13] and the genome size was estimated based on the Kmer frequency-depth distribution. In this study, 98,556,357,522 k-mers were generated, and the peak kmer depth was 282.355 (Supplementary Fig. S3). The genome size was estimated to be approximately 349 Mb and the final cleaned data corresponded

to the coverage of about 328-fold. Repeat and heterozygosity rates were estimated to be 83.43% and 1.7%, respectively (Supplementary Table S3) .

Genome assembly of *I. rubescens* f.lushanensis

This study used two sequencing techniques (Nanopore ONT Ultra-long and PacBio HiFi) to assemble the T2T genome separately. Next-Denovo v2.5.0 (NextDenovo, RRID:SCR_025033) [14], NECAT v202000119 (NECAT, RRID:SCR_025350) [15], Flye v2.9-b1768 (Flye,RRID:SCR_017016) [16] and Hifiasm v0.16.1 (Hifiasm, RRID:SCR_021069) [17] genome assembly software were used to preliminarily assemble ONT ultra long and HIFI sequencing data, respectively. The preliminary assembly results of ONT ultra long/PacBio HiFi data were evaluated for qv value, and the results showed that the qv value of genome quality was 54.704, the assembled genome length was 382,388,585bp, and the contig N50 was 28,807,450bp (Supplementary Table S4) . Winnowmap v1.11 (Winnowmap, RRID:SCR_025349) [18] was used to fill in gaps in the genome gap interval, and then align with the gap-filled version of the genome (HiFi reads ≥ 10 kbp). Then samtools v1.10 (samtools, RRID:SCR_002105) [19] was used to filter the aligned fragments, and finally Racon v1.6.0 (Racon, RRID:SCR_017642) was used for error correction to obtain the final T2T version genome. We used software Benchmarking Universal Single-Copy Orthologs (BUSCO) v4.14 (BUSCO, RRID:SCR 015008) [20] to evaluate the integrity of the genome assembly(Supplementary Table S5). We identified whether our previous operations were correct by checking the abnormality of the Hi-C interaction heatmap signal. In this experiment, a total of 375,924,825 bp of genome sequence was mapped to 12 chromosomes, accounting for 100% (Supplementary Fig. S4 and Table S6).

T2T genome annotation of *I. rubescens* f.lushanensis

The annotation of genome repeat sequences began with the denovo method, employing software RepeatModeler v1.0.11 (RepeatModeler, RRID:SCR 015027) [21] to predict model sequences based on the genome sequence. Additionally, software

LTR_Finder v1.2 (LTR_Finder, RRID:SCR_015247) [22] was utilized to predict LTR sequences, followed by LTR_retriever (LTR_retriever, RRID:SCR_017623) [23] to eliminate redundancy in the predicted sequences to produce non-redundant LTR sequences. These two types of denovo sequences were then combined to form the denovo repeat sequence library. Subsequently, the RepBase library [24] was integrated with the denovo library [25] and aligned using RepeatMasker v4.0.9 (RepeatMasker, RRID:SCR 012954) [26] to predict repeat sequences and generate the Denovo + repbase results. RepeatProteinMask (sub software of RepeatMasker) was employed to predict repeat sequences of the TE_proteion type, yielding TE proteins results. Finally, all predicted repeat sequences were merged and redundant elements were eliminated to obtain the final set of genome repeat sequences (Combined TEs). This analysis found that the total repetitive sequences in the genome of *I. rubescens* f.lushanensis accounted for 58.47% of the entire genome(Supplementary Fig. S5 and Table S7).

In this study, transcriptome prediction, homology prediction and ab initio prediction were used to predict the gene structure, the full-length sequences obtained by Nanopore ONT Ultra-long and PacBio Hifi sequencing were compared with the genome using minimap2 v2.17-r941 (Minimap2, RRID:SCR_018550) [27, 28], and then the bam files of the comparison results were combined and the transcripts were reconstructed by StringTie v2.1.4 (StringTie, RRID:SCR 016323) [29]. Then the predicted transcripts were predicted by TransDecoder v5.1.0(TransDecoder, RRID:SCR_017647) to predict the coding box, and finally the predicted coding genes were obtained. Homology prediction selected the protein sequence files of near source species for prediction analysis, and the homologous protein sequences were aligned to the genome using Tblastn v2.7.1 (Tblastn, RRID:SCR_011822) [30], and then the Exonerate v2.4.0 (Exonerate, RRID:SCR_016088) [31, 32] was used to predict transcripts and coding regions based on the comparison results, and Augustus v3.3.2 (Augustus, RRID:SCR 008417) [33] was used for ab initio prediction. GlimmerHMM v3.0.4 (GlimmerHMM, RRID:SCR_002654) [34] performed denovo prediction based on the results of homologous prediction. Finally, MAKER v2.31.10 (MAKER,

RRID:SCR 005309) [35] was used to integrate the genes predicted by various methods. A total of 34865 genes were predicted in the T2T genome structure prediction, with an average mRNA length of 3787.28bp and an average CDs length of 1163.68bp (Supplementary Table S8). The predicted genes were annotated against several functional databases, including the NCBI nonredundant protein database (NR;) [36], the KEGG database [37], the Uniprot database [38], the Interpro database [39], the protein families database (Pfam;) [40], the Cluster of Orthologous Groups for eukaryotic complete genomes (KOG) [41] database and Gene Ontology (GO) [42, 43]. It was found that 94.32% of all predicted genes could be annotated with the following protein related databases: KEGG(22.56%), KEGG Pathway(17.73%), NR (89.15%), Uniprot (88.12%), GO (65.21%), KOG (3.20%), Pfam (64.55%), and Interpro (92.06%) (Supplementary Table S9).

Comparison of genome assembly and annotation information

This assembly of *I. rubescens* f.lushanensis (hereafter mentioned as *I. rubescens*-LS) represents the highest continuity and completeness compared with the recently released genome assemblies for the published genome of *I. rubescens* (Hemsl.) Hara (hereafter mentioned as *I. rubescens*-JY) [7], In order to clearly display the genomic information of the two forms of *I. rubescens*, we conducted a comparative analysis of their genomic information. Based on K-mer distribution analysis, the genome size of *I. rubescens*-LS was estimated to be 349 MB, with a heterozygosity of 1.7%. In comparison, the genome size of *I. rubescens*-JY was 384 MB and the heterozygosity was 1.6%. The total data volume generated by *I. rubescens*-LS was 139.07 Gb, much more than that of *I. rubescens*-JY (55.21 Gb). Relying on the Hi-C technology, A total of 89.15Gb of data was obtained of *I. rubescens*-LS, all bases obtained by sequencing were attached to 12 chromosomes, with coverage rates of 100% and 94.37% for *I. rubescens*-LS and *I. rubescens*-JY, respectively [7]. The genome Contig N50 of *I. rubescens*-LS was 28.8 MB, much longer than that of *I. rubescens*-JY (0.3 MB). In addition, BUSCO (Benchmarking Universal Single-Copy Orthologs) analysis between *I. rubescens*-LS and *I. rubescens*-JY showed that the completeness of the genome

assembly was 94.4% and 88% (Table 1), respectively. These results demonstrated the outstanding quality of the genome assembled for *I. rubescens*-LS, which together with the published *I. rubescens*-JY genome laid the foundation for comparative genomic analysis of these two forms. (Figure 1).

Table 1. Summary of genome assembly of *I. rubescens*-LS and comparison with the *I. rubescens*-JY genome.

Item	*I. rubescens*-LS	*I. rubescens*-JY
Size of genome (MB)	349	384
Total_Data (Gb)	139.07	55.21
Contig N50 (MB)	28.8	0.3
Gene chromosome coverage (%)	100	94.3
Heterozygosity rate (%)	1.7	1.6
GC_content (%)	35.95	N
Pseudochromosomes	12	12
Percentage of LTR (%)	58.47%	55%
Protein-coding genes	34,865	35,436
Non-coding RNA	8032	3456
BUSCO (%)	98.9%	88%
Reference	This study	(Wang et al., 2022)

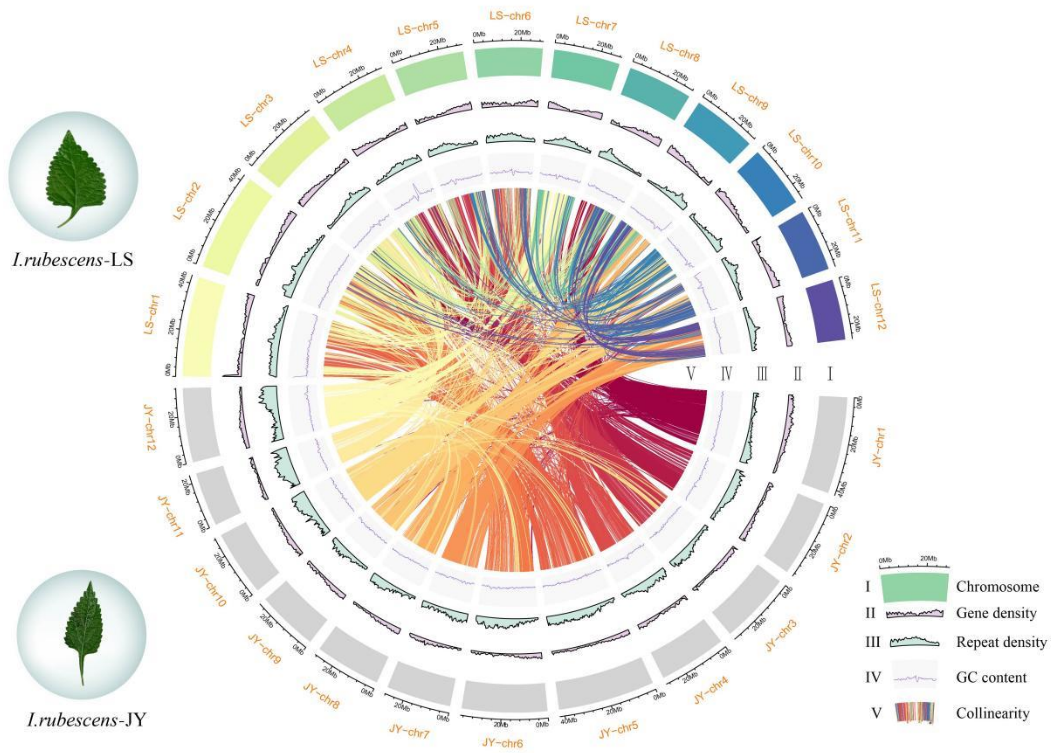

Figure 1. Genome assembly characterization and chromosome locations of *I. rubescens*-LS and *I. rubescens*-JY.

Landscape of *I. rubescens*-LS and *I. rubescens*-JY genomes: I collinearity, II GC content, III repeat density, IV gene density, V chromosome.

The BUSCO evaluation values of the *I. rubescens*-LS and *I. rubescens*-JY genomes'annotation results were both higher than 90%, proving that the annotation results were reliable (Supplementary Table S5). The total numbers of protein-coding genes in *I. rubescens*-LS and *I. rubescens*-JY were 34,865 and 30,789, the average lengths of the mRNAs were 4,110.25 bp and 3,711.47 bp, and the average lengths of CDS were 1,209.58 bp and 1,200.50 bp, respectively (Supplementary Table S8). The proportions of repetitive sequences in the entire genome of *I. rubescens*-LS and *I. rubescens*-JY were 58.47% and 55%, respectively. The main repeat type in both genomes was LTRs (long terminal repeats), with proportions of 28.98% and 25.15%, respectively. The main retrotransposons were LTR/Copia and LTR/Gypsy, with 7.57% of LTR/Copia and 17.35% of LTR/Gypsy in *I. rubescens*-LS, and 7.56% of LTR/Copia

and 14.07% of LTR/Gypsy in *I. rubescens*-JY (Supplementary Fig. S6A). A total of 8,032 and 3,456 non-coding RNAs were found in *I. rubescens*-LS and *I. rubescens*-JY, respectively. There was a significant difference in the amount of rRNAs between the two *I. rubescens* forms, which was specifically reflected in the 5sRNA amount (Supplementary Fig. S6B). With the functions of constituting ribosomes, catalyzing protein synthesis, and recognizing promoters and stops, rRNAs played a crucial role in protein synthesis, and was generally regarded as conservative. Therefore, the different number of rRNA annotated might be caused by the difference in the completeness of the two genome assemblies.

Identification and analysis of orthologous genes

Identification of homologous genes was a very important aspect of evolutionary analysis. Firstly, based on all amino acid sequences of the selected species, Orthofinder v2.3.12 (OrthoFinder, RRID:SCR_017118) [44] was used to cluster gene families, and Blastp v2.6.0 (Blastp, RRID:SCR_001010) [30] was used for comparison. The statistical analysis of gene family identification results showed that a total of 55,076 orthologous gene families were found in all species, including 465,541 genes, among which the number of single-copy genes was 158, and the number of gene families common to all species was 4,119, including 151,827 genes. There were 2,817 gene families unique for *I. rubescens*-LS, including 3,920 genes (Figure 2A). This analysis used Last v1170 (Last, RRID:SCR_006119) [45] to compare the gene sequences of *I. rubescens*-LS and *I. rubescens*-JY to determine similar gene pairs. Then JCVI v0.9.13 (JCVI, RRID:SCR_011269) [46] was used to determine whether similar gene pairs were adjacent on the chromosome according to the annotation file (gff3), so as to finally obtained the genes in all collinear blocks. The collinearity maps of *I. rubescens*-LS, *I. rubescens*-JY and *Arabidopsis thaliana* suggested that a whole-genome duplication event occurred between the two forms of *I. rubescens* (Figure 2B). The number of shared gene families between *I. rubescens*-LS and *I. rubescens*-JY accounts for 75% of the total gene families in their genomes. The significant overlap in the gene families suggested that the two forms of *I. rubescens*

were closely related (Supplementary Figure S7A). Most genes were conserved in *I. rubescens*-LS and *I. rubescens*-JY, implying that specific and unique gene families might cause the differences in metabolites. To verify this assumption, Clusterprofiler v3.19 (Clusterprofiler, RRID:SCR_016884) [47] was used to perform on the unique gene family members found in the two forms of *I. rubescens* (Supplementary Figure S8A and Supplementary Figure S8B), screening for genes enriched in the diterpenoid synthesis pathway, followed by locating them on chromosomes. It turned out that 14 unique genes in *I. rubescens*-JY and *I. rubescens*-LS are enriched in the diterpenoid synthesis pathway (Supplementary File 1, Supplementary Figure S7B and Supplementary Figure S7C). This result revealed that the abundant variations in diterpene synthetic genes might contribute to the chemotypic variation of *I. rubescens*-LS and *I. rubescens*-JY.

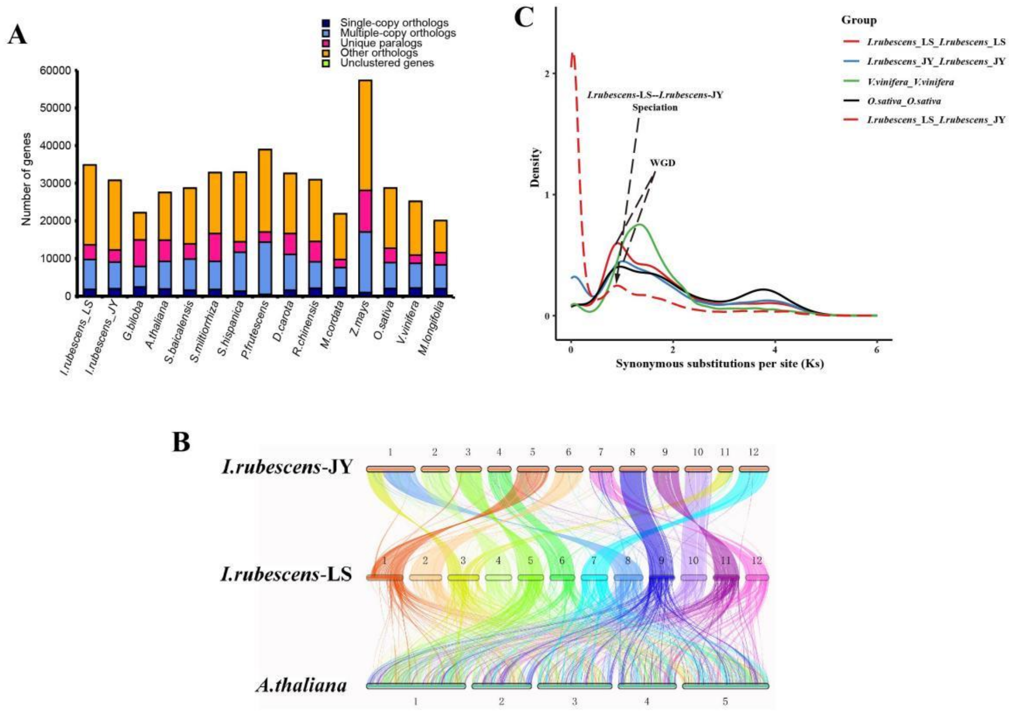

Figure 2. Genome-wide collinearity analysis and KS analysis of *I. rubescens*

(A) Number of homologous genes in different species.

(B) Collinearity analysis between *I. rubescens*-LS, *I. rubescens*-JY, and *A.*

thaliana chromosomes

(C) Curve fitting analysis of the distribution of synonymous substitution rates (Ks) of homologous genes.

Phylogenetic and duplication analysis of whole-genome

The software Muscle v3.8.31 (Muscle, RRID:SCR_011812) [48] was used to perform multiple sequence alignment of protein sequences of each single copy gene family, and then Trimal v1.2rev59 (Trimal, RRID:SCR_017334) [49] was used to filter the alignment results, and then the filtered alignment results were combined. Finally, the ML species phylogenetic tree was constructed based on the merged results using RAXML v8.2.10 (RAxML, RRID:SCR_006086) [50], Based on the topological structure of phylogenetic tree and fossil time node table [51], MCMCtreeR v4.9 (MCMCtreeR, RRID:SCR_025348)was used to estimate the differentiation time of selected species. The current classification of *I. rubescens*-LS and *I. rubescens*-JY was based on plant classification and chemical composition classification. Phylogenetic analysis based on whole-genome data was expected to more accurately and objectively determine the genetic relationship between the two forms of *I. rubescens*. For this purpose, genomes of 15 representative plants with good assembly quality and located in different evolutionary branches were downloaded from the NCBI (National Center for Biotechnology Information) database to conduct a comparative analysis of *I. rubescens*-LS and *I. rubescens*-JY from an evolutionary perspective (Supplementary Table S10). These species were distributed in eight major families, including the gymnosperm *Ginkgo biloba* (maidenhair tree) and the angiosperms *Arabidopsis thaliana* (thale cress), *Scutellaria baicalensis* (Baikal skullcap), *Salvia miltiorrhiza* (Chinese salvia), *Mentha longifolia* (horsemint), *Salvia hispanica*, *Perilla frutescens* (beefsteak-mint), *Daucus carota* subsp. *Sativus*, *Rosa chinensis* (China rose), *Macleaya cordata*, *Zea mays* subsp. *mays* (maize), *Oryza sativa* (Asian cultivated rice), and *Vitis vinifera* (wine grape). Genome phylogenetic analysis showed that seven species (or forms) from the family Lamiaceae were clustered as monophyletic. Among them, *Scutellaria baicalensis* was first separated from the others at about 63.1 MYA

(million years ago), and the remaining 6 species (or variants) were divided into two categories (*Salvia miltiorrhiza*, *Mentha longifolia*, *Salvia miltiorrhiza*) and (*Perilla frutescens, I. rubescens*-LS and *I. rubescens*-JY) at about 41.6 MYA. *I. rubescens* and *Perilla* separated the latest, at about 37.2 MYA, suggesting a close genetic relationship between them. This was consistent with the results of a previous phylogenetic study on common genera of labialis [52]. *I. rubescens*-LS and *I. rubescens*-JY belong to separate groups, which divided at about 8.7 MYA. CAFE v3.1 (CAFE, RRID:SCR_005983) [53] was used to estimate the number of gene family members in the ancestor of each branch based on the species evolutionary tree and gene family clustering results. Compared with their most recent common ancestor (MRCA), gene families had obviously contracted. Among them, the numbers of contractions of the *I. rubescens*-LS and *I. rubescens*-JY gene families were 567 and 1963, and the numbers of expanded gene families were 616 and 897, respectively. These numbers were relatively lower than most other Lamiaceae species, suggesting that *I. rubescens* was relatively conservative compared to other plants of the Lamiaceae family (Figure 2).

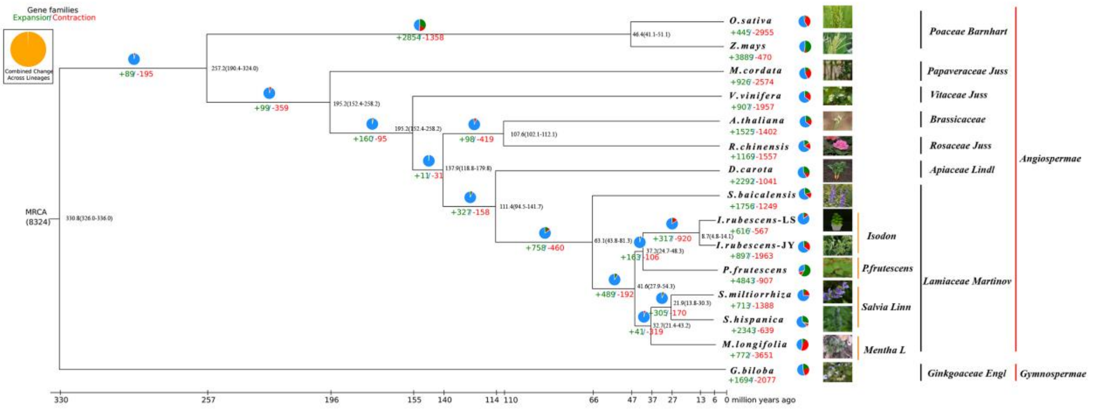

Figure 2. Inferred phylogenetic tree with 158 single-copy genes from 15 plant species (or forms).

Gene family expansions are indicated in green, and gene family contractions are indicated in red. The timing of WGD (Whole-genome doubling) is superimposed on the tree. Divergence times were estimated by maximum likelihood (PAML).

In order to have a clearer and more in-depth understanding of the whole-genome

duplication event in *I. rubescens*, PAML v4.9 (PAML, RRID:SCR_014932) [54] was used to calculate the ratio of Ka/Ks and ggplot2 v2.2.1 (ggplot2, RRID:SCR_014601) [55] was used to draw a density map. Grape and rice, the classic species of genome doubling events, were used as references for Ks curve fitting analysis (Figure 2C), it was found that the median KS curves of *I. rubescens*-LS and *I. rubescens*-JY peaked at the same position. In addition, the peak of the separation curves and the peak of the whole-genome duplication event overlap were also at the same position. Taking together the results of phylogeny and divergence time analysis, we could reasonably speculate that at 8.7 MYA, a whole-genome duplication event occurred between *I. rubescens*-LS and *I. rubescens*-JY, which led to evolutionary differentiation between the two forms. The multiplied genes had multiple possibilities such as pseudogenization, neofunctionalization, and subfunctionalization. Among them, the neofunctionalization and pseudogenization genes might lead to the occurrence or disappearance of unique metabolites, thus causing the chemotypic variation of *I. rubescens*.

Comparative analysis of genomic structure variations

MUMmer v4.0.0rc1 (MUMmer, RRID:SCR_018171) [56] and SyRI v1.6 (SyRI, RRID:SCR_023008) [57] were used to conduct whole-genome comparison and mutation type detection, respectively, using the genome of *I. rubescens*-LS as the reference genome, TBtools v2.069 (TBtools, RRID:SCR_023018) [58] was used to annotate gene function and pathway enrichment and map the distribution of gene family genes on the chromosome. *I. rubescens*-LS and *I. rubescens*-JY were both diploid (2n=24). The *I. rubescens*-LS genome with higher genome assembly quality was used as the reference genome to detect genomic SNP (single nucleotide polymorphism), PAVs (presence/absence variations), and SVs (structure variations). The whole genomes of *I. rubescens*-LS and *I. rubescens*-JY corresponding to a total of 367,304,545 bp were used in the collinear sequence alignment, with a coverage rate of 95.68% (Figure 4A). SNP variations led to changes in amino acids and stop codons, but did not cause frameshift mutations in protein-coding sequences. There were 2,996,964

SNP variations, of which 1,386,807 were in the genetic region, and 1,610,157 in the genetic intervals (Supplementary Figure S9A and Supplementary Figure S9B). Compared with SNP variations, SVs usually referred to large-scale sequence changes and positional relationship changes on the genome including long-segment chromosomal inversions and chromosomal translocations and duplication, which had a greater impact on the genome. In humans, such structural variants were associated with many diseases (including autism, obesity, schizophrenia, cancer, etc.) [59]. In plants, SVs were associated with many phenotypic variations and biotic/abiotic stresses [60]. A total of 56,399 SVs were found in this study, mainly distributed in intergenic regions. The predominant type was repeated mutations, accounting for 53.7% (Figure 4B). Another important type of structural variation was PAV, which might be the reason why individuals produced different traits (disease resistance, cold resistance, etc.) [61]. A total of 34,443 PAVs were found, with almost equal numbers of presence and absence mutations, and the length span of the mutations was large (Figure 4C). Genes affected by PAVs and SVs were identified and subjected to pathway functional enrichment analysis (Supplementary Figure S9C and Supplementary Figure S9D). Genes enriched in the diterpenoid synthesis pathway were annotated and located on the chromosome for visual analysis (Supplementary File 2 and Figure 4D). The results showed distribution of 35 mutated genes on 8 chromosomes, most of which were diterpene synthase genes, together with a CYP450 encoding gene and 5 genes of the gibberellin synthesis pathway. Based on the above results, This result supported the speculation that the variations in diterpenoid synthetic genes might be one of the driving forces for the chemotypic variation of *I. rubescens*.

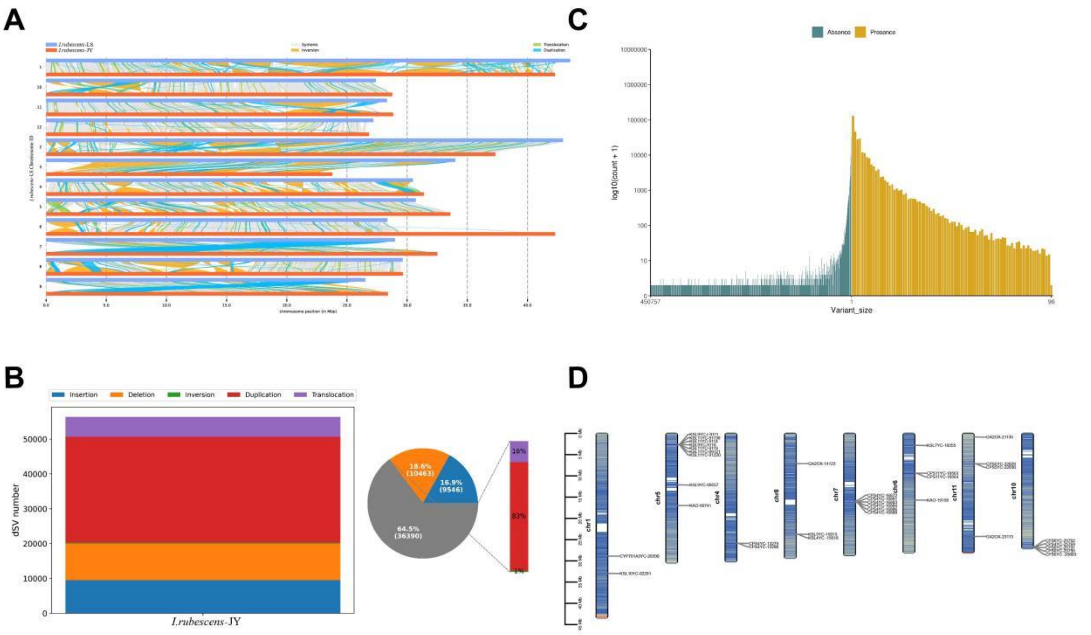

Figure 4. Analysis of whole-genome structural variation of *I. rubescens*-LS and *I. rubescens*-JY

(A) Collinear syri diagram of *I. rubescens*-LS and *I. rubescens*-JY.

The blue skeleton displays the *I. rubescens*-LS genome, the red skeleton displays the *I. rubescens*-JY genome, and the middle displays the collinearity and identified SV distribution.

(B) Statistical results of SV types and quantities.

(C) Length distribution of PAVs.

(D) Chromosome distribution map of genes affected by SV and PAV on the reference genome.

Discussion

The biosynthesis of active ingredients in traditional Chinese medicine had emerged as a research focus in recent years. However, it was often hindered by the unclear molecular mechanism and pathway for synthesizing the target natural product. Analysis of the complete genome information could help us understand the evolutionary relationship among medicinal plants with different types or contents of active ingredients and shed light on the biosynthesis pathway and its regulation

mechanism [62]. Due to differences in genome duplication, transposon expansion, and repetitive sequences, genome sizes varied widely among different species. About 16 medicinal plants in the Lamiaceae family had completed genome sequencing, and their size ranged from 338 Mb to 2.07 G (Supplementary Table S11). Most of the genome sequencing results included incomplete assembly of gaps, centromere, and telomere regions, as well as high heterozygosity, and high repetitive assembly errors or incompleteness. Recently, T2T (Telomere-to-Telomere) genome sequencing employing multiple sequencing platforms and high-depth sequencing had emerged to assemble Gap-free or near-Gap-free high-quality genomes. This sequencing method overcame the difficulty of assembling centromeres or highly repetitive regions, and greatly improved the continuity and integrity of chromosomes [63, 64].In the present study, the complete genome information of *I. rubescens*-LS was revealed for the first time, with a size of 349 Mb and a GC content of 35.78%, similar to that of the *I. rubescens*-JY previously sequenced. The high genome heterozygosity (1.7%) and duplication degree (83.43%) suggested that the genome of *I. rubescens*-LS is highly heterozygous and highly repetitive. Generally, a genome with a heterozygous rate >0.5% and a repetitive sequence content >50% is considered a highly heterozygous and highly repetitive genome [65].) The quality of the *I. rubescens*-LS genome obtained using T-T genome sequencing had been dramatically improved compared with the previous sequenced *I. rubescens*-JY genome. The successful sequencing an annotation of the complete genome sequences of the two closely related *I. rubescens* forms would provide the basis for understanding the origin of the chemotypic variation and meanwhile supply genetic elements for reconstruction of the synthetic pathway for the valuable plant natural products in model chasses.

Whole genome evolution helped to understand the genetic relationship between the two forms of *I. rubescens*. The number of gene families shared by *I. rubescens*-LS and *I. rubescens*-JY was 17632, accounting for 75.4% of the total number of families, proving that most genes of the two forms were derived from the same ancestor. They had apparent similarities in structure and function, encoding similar proteins and performing similar functions. As an important driving factor for the emergence of new

traits and functions, whole-genome duplication events occured throughout the entire plant evolution process. Both *I. rubescens*-LS and *I. rubescens*-JY experienced a whole-genome duplication event, and the whole-gene duplication peaks and the speciation peak of these two forms coincided. *I. rubescens*-LS and *I. rubescens*-JY diverged about 8.7 million years ago during the Tortonian stage of the late Miocene. Taken together with the species divergence time, it could be reasonably inferred that a whole-genome duplication event occurred between the two forms of *I. rubescens* 8.7 million years ago, leading to their separation. Through genome-wide evolutionary analysis, we provided a molecular basis for the phylogenetic classification of *I. rubescens*-JY and *I. rubescens*-LS. This result supported current plant and chemical taxonomy. Variations covering SNP, SV, and PAV were found in the two forms. SNP, as a high-density, genetically stable, and representative variation representative, affectd gene function by changing the protein structure or gene expression level [66]. Many SNPs were found in the intergenic and intron regions of genes, which might generate changes in the promoters/terminators controlling gene expression. Compared with SNPs, SVs and PAVs were more enriched in the diterpenoid synthesis pathway, particularly in diterpene synthase genes. In consistence with the results of the gene variation analysis, the results of the unique gene family analysis of *I. rubescens*-JY and *I. rubescens*-LS showed that most of the unique genes enriched in the diterpenoid synthesis pathway were diterpene synthase genes. Therefore, we could carry out functional verification analysis on these diterpene synthase genes affected by structural variation in the later stage, and provided more molecular basis for the analysis of diterpene biosynthesis pathway and chemical type variation of related species in *I. rubescens*.

Data availability

The data supporting the findings of this work are available in the paper and its supplementary information file. Genome Data NCBI BioProject number: PRJNA1089255 and BioSample accession: SAMN40534968.

Additional files

Figure S1. HiFi data length statistical histogram.

Figure S2. Histogram showing distribution of read lengths across quality passing sequences. The two black vertical lines from left to right are the average length and N50 of all data, and different colors correspond to pass reads and fail reads respectively.

Figure S3. K-mer frequency distribution at k-mer size of 19.

Figure S4. Interval interaction diagram in chromosome. The blue border represents each chromosome.

Figure S5. Statistical map of genome repeat sequence distribution. DNA: DNA transposons, LINE:long interspersed nuclear element, SINE:short iterspersed element, LTR:long terminal repeat.

Figure S6. Genomic repeated sequences and non-coding RNA annotation information of *I. rubescens*-LS and *I. rubescens*-JY. (A)Comparison of genomic repeated sequences of *I. rubescens*-LS and *I. rubescens*-JY; (B)Comparison of non-coding RNAs in *I. rubescens*-LS and *I. rubescens*-JY.

Figure S7. Analysis of unique gene families in *I. rubescens*-LS and *I. rubescens*-JY (A)Shared and unique gene families in the *I. rubescens*-LS and *I. rubescens*-JY genomes; (B)Chromosome distribution map of unique diterpenoid pathway genes in *I. rubescens*-JY; (C)Chromosome distribution map of unique diterpenoid pathway genes in *I. rubescens*-LS.

Figure S8. Bubble plot reference for KEGG enrichment analysis of unique genes in *I. rubescens*-LS and *I. rubescens*-JY, (A)Bubble plot reference for KEGG enrichment analysis of unique genes in *I. rubescens*-LS, (B)Bubble plot reference for KEGG enrichment analysis of unique genes in *I. rubescens*-JY.

Figure S9. Distribution of SNP, SV, and PAV. (A)SNP Location distribution ; (B)SNP function annotation; (C)Bubble plot reference for KEGG enrichment analysis of genes affected by SV on the reference genome; (D)Bubble plot reference for KEGG enrichment analysis of genes affected by PAV on the reference genome.

Table S1. Statistical table of data volume information of the third generation

sequencing (ONT ultra long), rank is the data length gradient,>0 is all data; Flag is the data type, all is the total sequencing data, pass is the valid sequencing data, and fail is the filtered data.

Table S2. Statistical table of Pacbio Hifi.

Table S3. Estimation of genome characteristics based on 19-mer statistics.

Table S4. Statistical table of QV value of genome assembly.

Table S5. Comparative analysis of BUSCOs annotated genome structure of *I. rubescens*-LS and *I. rubescens*-JY.

Table S6. Statistical map of genome chromosome length distribution.

Table S7. Genome repeat sequence distribution of *I. rubescens*-LS.

Table S8. Comparison of genomic protein-encoding gene annotations of *I. rubescens*-LS and *I. rubescens*-JY.

Table S9. Statistics of functional annotation results of coding genes.

Table S10. Sequence information used in phylogenetic tree analysis for genome evolution

Table S11. Genome information of medicinal plants of Lamiaceae

Supplementary File 1. Statistical table of functional enrichment analysis of specific gene family of *I. rubescens*-JY and *I. rubescens*-LS

Supplementary File 2. Functional enrichment analysis of diterpenoid synthesis pathway genes in *I. rubescens*-LS under PVA and SV influence.

Competing interests

The authors state that they do not have conflicts of interest related to this work.

Author contributions

Suiqing Chen and Hao Yang conceived and initiated this study. Lidan Ye and Hao Yang designed the yeast engineering experiments. Hao Yang performed the genome sequencing and bioinformatics analysis. Hongwei Yu and Lidan Ye provided yeast strain materials and methodology. Hao Yang and Jinlu Liu performed most of the experiments, assisted by Ni He, Le Zhao, Xiuyu Liu, Shujuan Xue, Xiaoya Sun, Zhang

Liping, Lili Wang, Yu Fu, Jingfan Yang, Rui Ma and Bao Zhang. Hao Yang wrote the manuscript, and Suiqing Chen, Conglong Lian, and Lidan Ye revised the manuscript. All authors read and approved the final version of the manuscript.

Acknowledgements

This study was funded by the Joint Fund Project of Science and Technology Research and Development of Henan Province (Superior discipline Cultivation) (Grant No.232301420078), National Natural Science Foundation of China (Frant No. 81173486), Chinese Herbal Medicine Industry Technology System of Henan Province (Grant No.14 [2018]).

References

1. Dong R, Gao H, Liu Z. Research progress on the biological activities of diterpenoids from Isodon. China Pharmacy. 2010;**21**:651-53.
2. Qiu X, Zhang Y, Chen Y. et al. Utilization status and developmental potential of Lamiaceae as medicinal plant resources in China. Chinese journal of applied and environmental biology. 2023;**29**:346-56.
3. Xu z. Isodon rubescens is expected to become the "second paclitaxel". Modern Chinese medicine. 2007;**9**:43-45.
4. Xie T, Yang Z, Xu W. et al. Research progress on chemical constituents, pharmacological effects and clinical application of *Isodon rubescens.* Chinese Traditional and Herbal Drugs. 2022;**53**:317-25.
5. Wei C, Guo B, Zhang C. et al. Perilla resources of China and essential oil chemotypes of Perilla leaves. China journal of Chinese materia medica. 2016;**41**:1823-34.
6. Wei J. Primary Study on the formation mechanism of chemotype in Isodon rubescens (Hemsl) H. Hara [Master's thesis]. Zhengzhou University: Zhengzhou University; 2012.
7. Sun Y, Shao J, Liu H. et al. A chromosome-level genome assembly reveals that tandem-duplicated CYP706V oxidase genes control oridonin biosynthesis in the shoot apex of Isodon rubescens. Mol Plant. 2023;**16**:517-32.
8. Porebski S, Bailey LG, Baum BR. Modification of a CTAB DNA extraction protocol for plants containing high polysaccharide and polyphenol components. Plant Molecular Biology Reporter. 1997;**15**:8-15.
9. David, Deamer, Mark. et al. Three decades of nanopore sequencing. Nature biotechnology. 2016;**34**:518-24.
10. Magi A, Semeraro R, Mingrino A. et al. Nanopore sequencing data analysis: state of the art, applications and challenges. Briefings in Bioinformatics. 2017:6.
11. Jain M, Olsen HE, Paten B. et al. The Oxford Nanopore MinION: delivery of nanopore sequencing to the genomics community. Genome Biology. 2016;**17**:239.

12. Liu B, Shi Y, Yuan J. et al. Estimation of genomic characteristics by analyzing k-mer frequency in de novo genome projects. Quantitative Biology. 2013;**35**:62-67.
13. Kingsford C. A fast, lock-free approach for efficient parallel counting of occurrences of k-mers. Bioinformatics. 2011;**27**:764.
14. Hu J, Wang Z, Sun Z. et al. NextDenovo: an efficient error correction and accurate assembly tool for noisy long reads. Genome biology. 2024;**25**:107.
15. Ying C, Fan N, Shang-Qian X. et al. Efficient assembly of nanopore reads via highly accurate and intact error correction. Nat Commun. 2021;**12**:1-10.
16. Kolmogorov M, Yuan J, Lin Y. et al. Assembly of Long Error-Prone Reads Using Repeat Graphs. Cold Spring Harbor Laboratory. 2018;
17. Haoyu C, Gregory T C, Xiaowen F. et al. Haplotype-resolved de novo assembly using phased assembly graphs with hifiasm. Nat Methods. 2021;**18**:170-75.
18. Jain C, Rhie A, Hansen NF. et al. Long-read mapping to repetitive reference sequences using Winnowmap2. Nature Methods. 2022;
19. Danecek P, Bonfield JK, Liddle J. et al. Twelve years of SAMtools and BCFtools. GigaScience. 2021;**10**
20. A. SF, Waterhouse RM, Panagiotis I. et al. BUSCO: assessing genome assembly and annotation completeness with single-copy orthologs. Bioinformatics. 2015;**31**:3210-12.
21. RepeatModeler - 1.0.10. http://www.repeatmasker.org/RepeatModeler/. Accessed 01 Jul 2017.
22. Ou S, Jiang N. LTR_FINDER_parallel: parallelization of LTR_FINDER enabling rapid identification of long terminal repeat retrotransposons. Cold Spring Harbor Laboratory. 2019;**10**:48.
23. Shujun O, Ning J. LTR_retriever: A Highly Accurate and Sensitive Program for Identification of Long Terminal Repeat Retrotransposons. Plant Physiol. 2017;**176**:1410-22.
24. W B, KK K, O K. Repbase Update, a database of repetitive elements in eukaryotic genomes. Mobile DNA. 2015;**6**:11.
25. Ultra-fast denovo assembler using long noisy reads. https://github.com/ruanjue/smartdenovo. Accessed 01 Dec 2017.
26. RepeatMasker. http://www.repeatmasker.org/. Access 01 Jul 2017.
27. Heng L. Minimap2: pairwise alignment for nucleotide sequences. Bioinformatics. 2018;**32**:2103-10.
28. Heng L. Minimap and miniasm: fast mapping and de novo assembly for noisy long sequences. Bioinformatics. 2016;**32**:2103-10.
29. Mihaela P, Geo M P, Corina M A. et al. StringTie enables improved reconstruction of a transcriptome from RNA-seq reads. Nat Biotechnol. 2015;**33**:290-5.
30. Camacho C, Coulouris G, Avagyan V. et al. BLAST+: architecture and applications. Bmc Bioinformatics. 2009;**10**:1-9.
31. A generic tool for sequence alignment.https://www.ebi.ac.uk/about/vertebrate-genomics/software/exonerate. Accessed 10 Jan 2018.
32. Guy St C S, Ewan B. Automated generation of heuristics for biological sequence comparison. BMC Bioinformatics. 2005;**6**:1-11.
33. Baertsch MSMDR, Haussler D. Using native and syntenically mapped cDNA alignments to improve de novo gene finding. Bioinformatics. 2008;**24**:637-44.
34. Arthur L D, Kirsten A B, Edwin C P. et al. Identifying bacterial genes and endosymbiont DNA with Glimmer. Bioinformatics. 2007;**23**:673-79.

35. Brandi L C, Ian K, Sofia M C R. et al. MAKER: an easy-to-use annotation pipeline designed for emerging model organism genomes. Genome Res. 2007;**18**:188-96.
36. National Center for Biotechnology Information. http://www.ncbi.nlm.nih.gov. Accessed 01 Dec 2017.
37. Ogata H, Goto S, Sato K. et al. KEGG: kyoto Encyclopedia of Genes and Genomes. Nucleic Acids Research. 1999;**27**:29-34.
38. UniProt: the Universal Protein Knowledgebase in 2023. Nucleic Acids Res. 2023;**51**:D523–D31.
39. Typhaine P-L, Matthias B, Sara C. et al. InterPro in 2022. Nucleic Acids Res. 2022;**51**
40. Pfam. http://pfam.xfam.org/. Accessed 01 Dec 2017.
41. The KOG Browser.http://genome.jgi-psf.org/help/kogbrowser.jsf. Accessed 01 Dec 2017.
42. Gene Ontology Consortium. http://www.geneontology.org. Accessed 01 Dec 2017.
43. Ashburner M, Ball CA, Blake JA. et al. Gene ontology: tool for the unification of biology. The Gene Ontology Consortium. Nature Genetics. 2000;**25**:25-9.
44. David M E, Steven K. OrthoFinder: phylogenetic orthology inference for comparative genomics. Genome Biol. 2019;**20**
45. Frith MC, Hamada M, Horton P. Parameters for accurate genome alignment. Bmc Bioinformatics. 2010;**11**:1-14.
46. Tang H, Bowers JE, Wang X. et al. Synteny and Collinearity in Plant Genomes. 2008;**320**:486-88.
47. Wu T, Hu E, Xu S. et al. clusterProfiler 4.0: A universal enrichment tool for interpreting omics data. The Innovation, Elsevier. 2021;**2**:1100141.
48. Fábio M, Matt P, Adrian R N T. et al. Search and sequence analysis tools services from EMBL-EBI in 2022. Nucleic Acids Res. 2022;**50**
49. Salvador C-G, José M S-M, Toni G. trimAl: a tool for automated alignment trimming in large-scale phylogenetic analyses. Bioinformatics. 2009;**25**:1972-73.
50. Alexandros S. RAxML version 8: a tool for phylogenetic analysis and post-analysis of large phylogenies. Bioinformatics. 2014;**30**:1312-13.
51. Sudhir K, Michael S, Jack M C. et al. TimeTree 5: An Expanded Resource for Species Divergence Times. Mol Biol Evol. 2022;**39**
52. GX H, A T, BT D. et al. Phylogeny and staminal evolution of Salvia (Lamiaceae, Nepetoideae) in East Asia. Ann Bot. 2018;**122**:649-68.
53. Sander B, Mathias L, Miguel A A-N. et al. CAFE: an R package for the detection of gross chromosomal abnormalities from gene expression microarray data. Bioinformatics. 2014;**30**
54. Ziheng Y. PAML 4: phylogenetic analysis by maximum likelihood. Mol Biol Evol. 2007;**24**:1586-91.
55. Wickham H. Ggplot2: Elegant Graphics for Data Analysis: ggplot2: Elegant Graphics for Data Analysis; 2016.
56. Guillaume M, Arthur L D, Adam M P. et al. MUMmer4: A fast and versatile genome alignment system. PLoS Comput Biol. 2018;**14**:e1005944.
57. Manish G, Hequan S, Wen-Biao J. et al. SyRI: finding genomic rearrangements and local sequence differences from whole-genome assemblies. Genome Biol. 2019;**20**:227.
58. Chengjie C, Ya W, Jiawei L. et al. TBtools-II: A "one for all, all for one" bioinformatics platform for biological big-data mining. Mol Plant. 2023;**16**
59. Audano P, Sulovari A, Graves-Lindsay T. et al. Characterizing the Major Structural Variant Alleles of the Human Genome. Cell. 2019;**176**:663-75.

60. Guo J, Cao K, Deng C. et al. An integrated peach genome structural variation map uncovers genes associated with fruit traits. Genome biology. 2020;**21**:258.
61. Alonge M, Wang X, Benoit M. et al. Major Impacts of Widespread Structural Variation on Gene Expression and Crop Improvement in Tomato. Cell. 2020;**182**:145-61.
62. Hu H, Shen X, Liao B. et al. Herbgenomics: A stepping stone for research into herbal medicine. Science China Life sciences. 2019;**62**:913-20.
63. Nurk S, Koren S, Rhie A. et al. The complete sequence of a human genome. Science (New York, NY). 2022;**376**:44-53.
64. Naish M, Alonge M, Wlodzimierz P. et al. The genetic and epigenetic landscape of the Arabidopsis centromeres. Science. 2021;**374**:3.
65. Zhang H, He Q, Xing L. et al. The haplotype-resolved genome assembly of autotetraploid rhubarb Rheum officinale provides insights into its genome evolution and massive accumulation of anthraquinones. Plant communications. 2024;**5**:100677.
66. Gao M, He Y, Wang GL. et al. Ca 2+ sensor-mediated ROS scavenging suppresses rice immunity and is exploited by a fungal effector. Cell. 2021;**184**:1-14.

Figure

Click here to access/download;Figure;Figure 4.tif

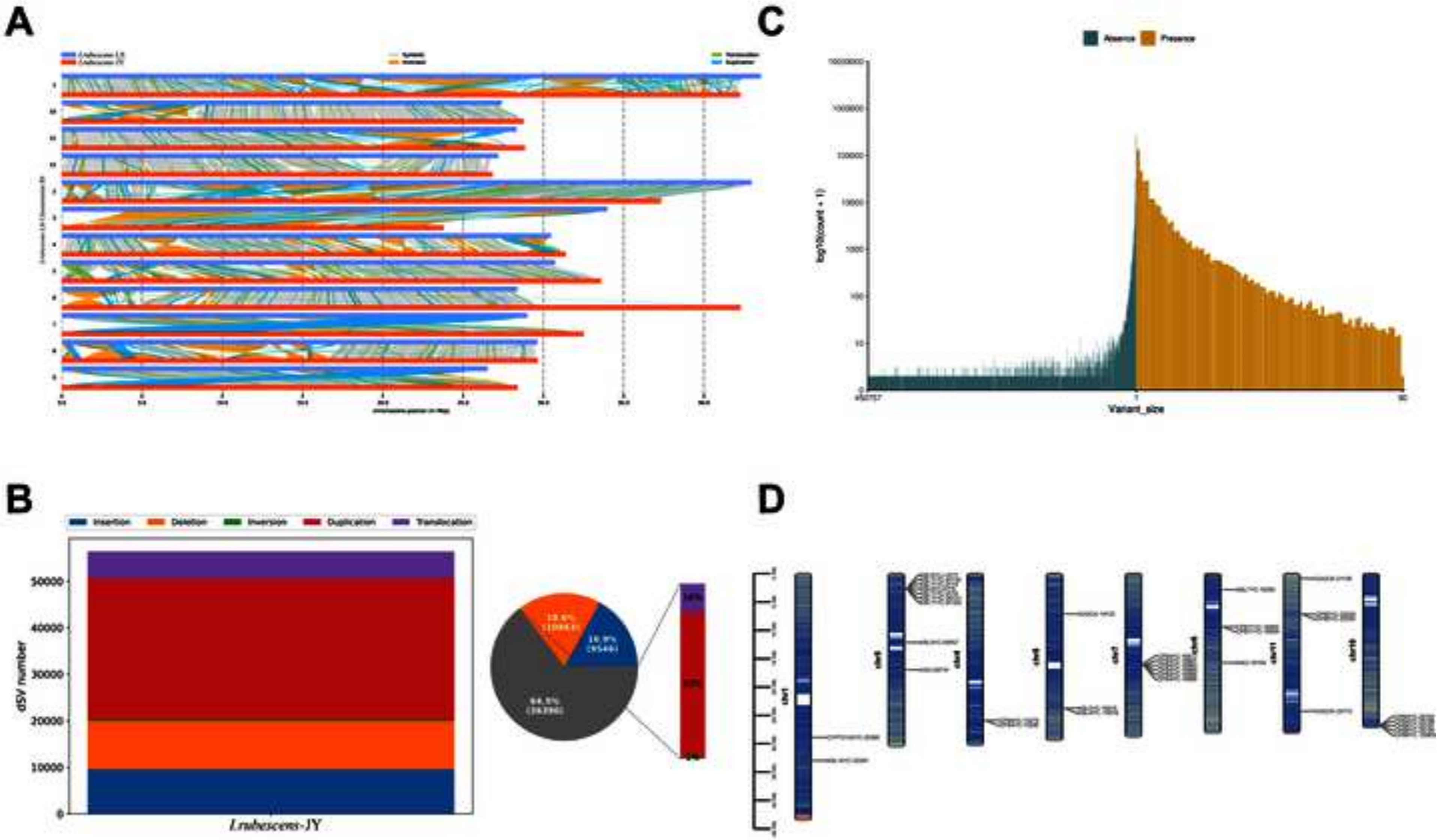

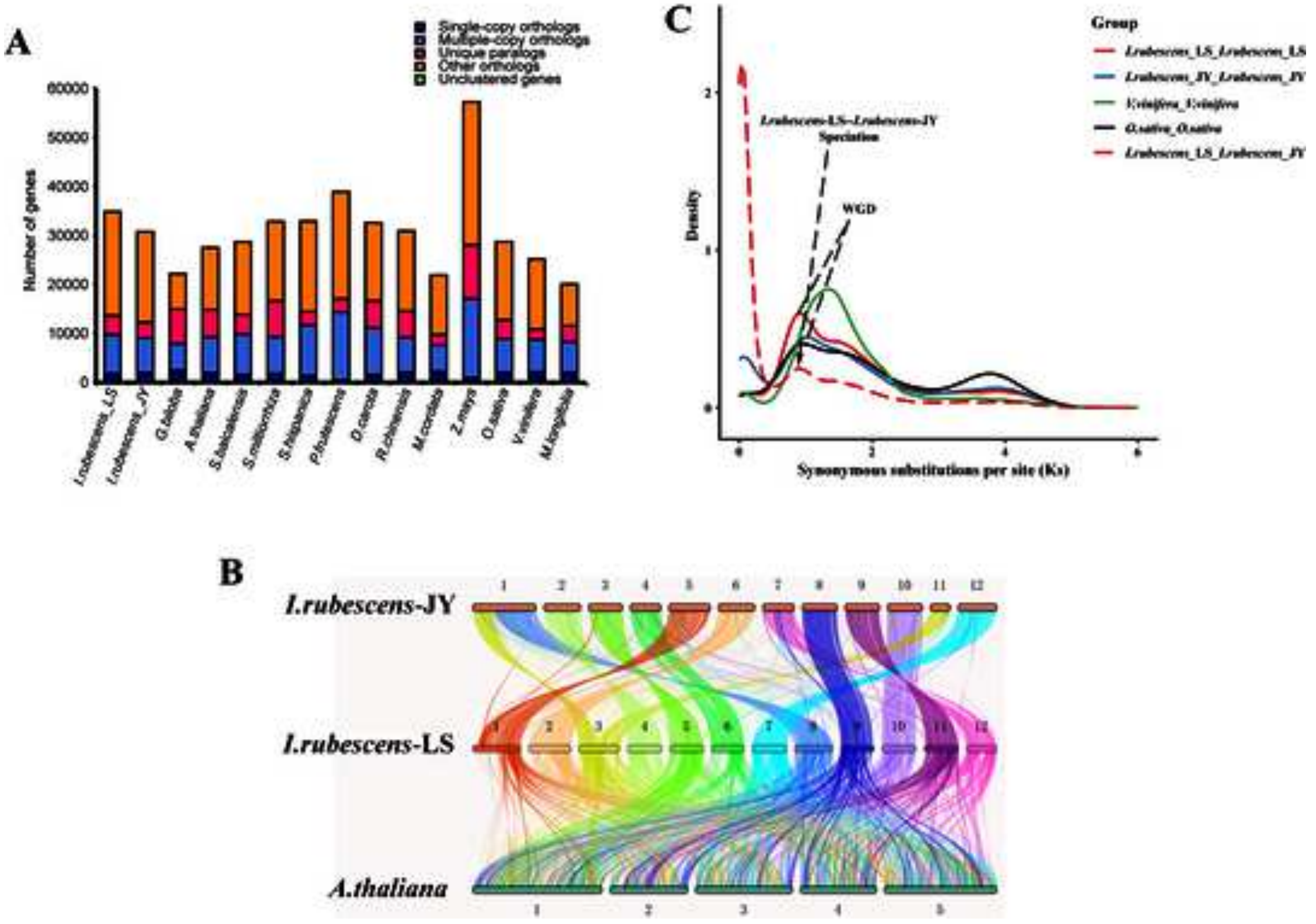
A
Single-copy orthologs
Multiple-copy orthologs
Unique paralogs
Other orthologs
Unclustered genes
Number of genes
60000
50000
40000
30000
20000
10000
0
B
I.rubescens-JY
I.rubescens-LS
A.thaliana
C
Group
WGD
Speciation
Density
Synonymous substitutions per site (Ks)

Figure Click here to access/download;Figure;Figure 2.tif

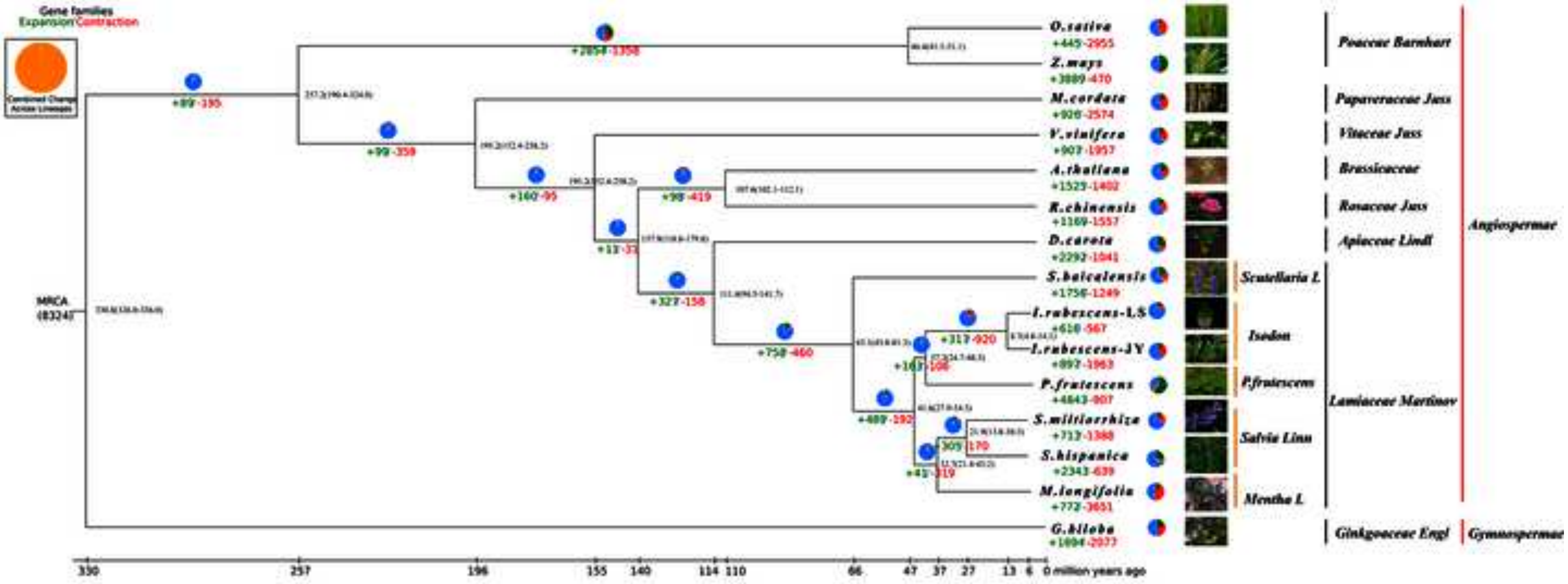

Click here to access/download;Figure;Figure 1.tif

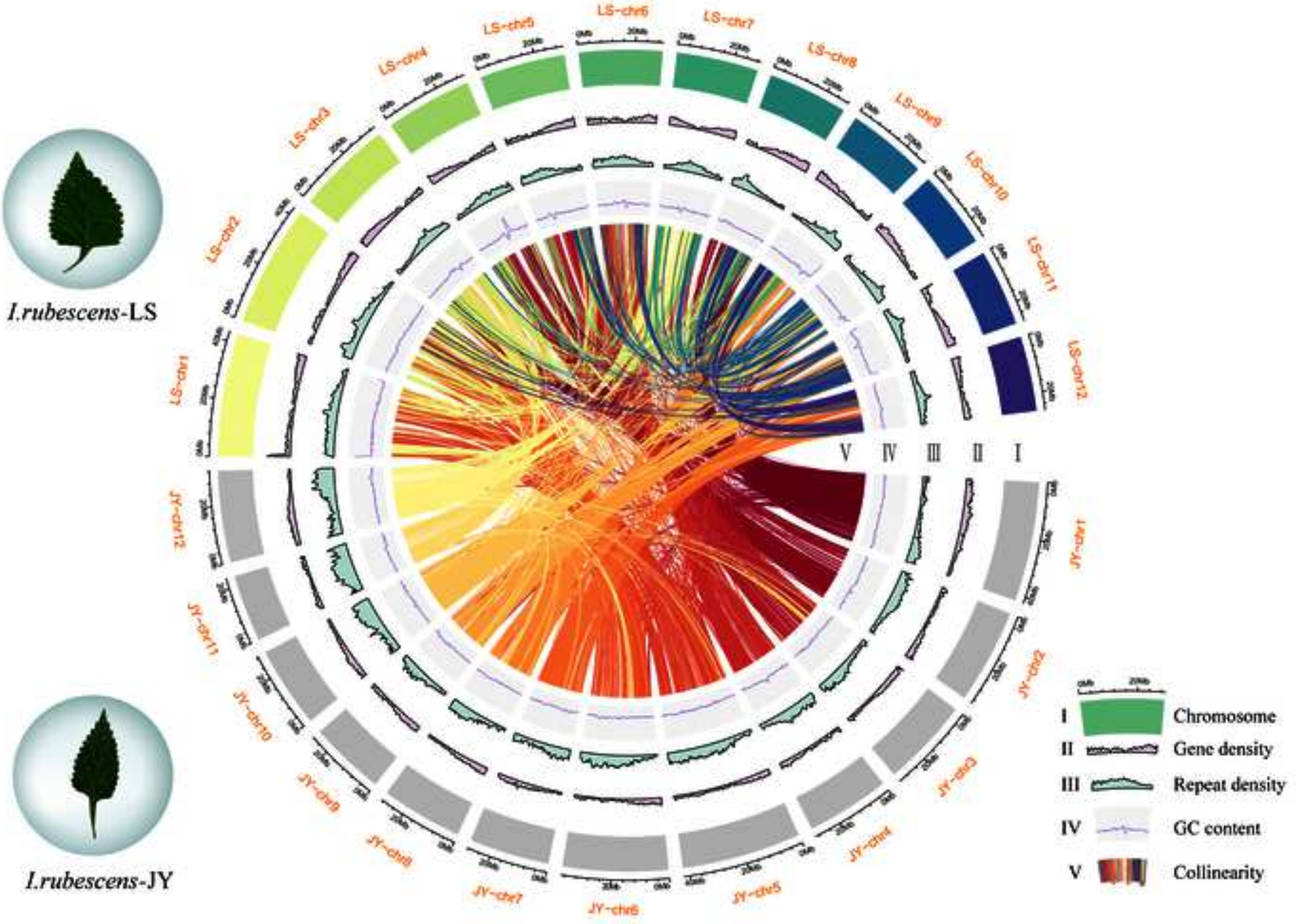

Click here to access/download
Supplementary Material
supplemental files.rar
