# Supplementary material for: High-quality assembly of the T2T genome for Isodon rubescens f. lushanensis reveals genomic structure variations between 2 typical forms of Isodon rubescens
Source: Gigascience. 2024 Oct 10;13:giae075. doi: 10.1093/gigascience/giae075 (PMC11466039; doi:10.1093/gigascience/giae075)
Supplement: giae075_Response_to_Reviewer_Comments_Original_Submission [file giae075_response_to_reviewer_comments_original_submission.pdf]

Author's Response To Reviewer Comments

Response to Reviewers' Comments

First of all, we extend our heartfelt gratitude for the editorial team's gracious offer of the opportunity to revise. We have meticulously reviewed the feedback provided by the two esteemed reviewers and crafted my responses accordingly. Additionally, we have carefully revised the manuscript. The point-to-point response to the reviewers' comments are presented below.
Reviewer 1

Q1: question about the name of the plant.

A1: Thank you for the comment. According to the botanical taxonomy, below the species level, there are divisions known as varieties, forms, and chemotypes. Variations of variety and form are two distinct approaches to characterizing the internal diversity of a species. In plant taxonomy, a variety is a rank within a species that refers to a group of plants that exhibit distinct morphological characteristics from the original species. Varieties typically possess stable genetic traits that can be maintained under natural conditions or through artificial cultivation. The form is a taxonomic rank below the variety, used to describe individual plants or groups that have minor differences in certain morphological characteristics from the variety or the original species. The subject of sequencing in this experiment is a form of Isodon rubescens, which is different from Isodon rubescens (Hemsl.) H.Hara in both plant morphology, chemical composition and Molecular phylogenetic tree. That is why we used the term "form" in the title. The species was previously recorded in the IPNI, but you need to search using the old Latin name（Rabdosia rubescens f. lushanensis）.Due to the change in the generic name of the genus from Rabdosia to Isodon, That is why we have used the new Latin name(Isodon rubescens f. lushanensisin) in this article.
In more details, Rabdosia rubescens f. lushanensis is a form of Rabdosia rubescens, which was first classified and named by Professor Zengyi Gao and Yaru Li. according to the plant morphological classification method[1]. Since the Latin name of the Isodon rubescens has been corrected (previously Rabdosia rubescens), this species has been referred to with two different Latin names in various articles. Both Rabdosia rubescens f. lushanensis and Isodon rubescens f. lushanensis refer to the same plant[2]. The group of Academician Sun handong from Kunming Institute of Botany, Chinese Academy of Sciences, extensively studied the chemical classification of Isodon rubescens[3-6], and their findings supported the classification of Isodon rubescens f. lushanensis as a distinct form of Isodon rubescens. The phylogenetic analysis of Isodon rubescens utilizing Inter-Simple Sequence Repeat (ISSR) and Random Amplified Polymorphic DNA (RAPD) techniques corroborated the taxonomic delineations[7]. This was further reinforced by chloroplast genomic analysis to distinguish between the intraspecific variation of Isodon rubescens: IR-L(I. rubescens f. lushanensis), and IR-J(I. rubescens (Hemsl.) Hara.)[8]. Therefore, the classification of I. rubescens f. lushanensis as a form of I. rubescens is supported by plant classification, chemical classification, and molecular classification.

We agree with your suggestion that DNA barcode molecular identification is useful in classification, but its ability to distinguish plants below the species level is limited. The combination of various classification methods such as traditional plant morphological classification, chemical component classification, and molecular phylogenetic classification, is usually regarded as sufficient.
To avoid future misunderstanding, we have revised the manuscript by adding the following sentence into the second paragraph of the background information section: "During the survey of I. rubescens germplasm resources, a new form was found, named I. rubescens f. lushanensis, which differs from I. rubescens (Hemsl.) Hara (the original form) in plant morphology[1], chemical composition[3-6], and molecular phylogenetic tree[7-8]."

Q2: Question about reference citation in Table 1.
A2: Thank you for the helpful comment. Sorry for our mistake. We have now corrected the reference.

Q3: Question about the selection of phylogenetic tree species.
A3: Thank you for your comment. First of all, there should be a misunderstanding. In the submitted

manuscript, we actually included the genome of Scutellaria baicalensis for the construction of the phylogenetic tree in Figure 2 (S. baicalensis). Secondly, we want to explain the reason why we included species such as Arabidopsis, rice, and grape, which are distantly related to the Lamiaceae family, in the analysis. We wanted to carry out WGD (Whole Genome Duplication) event analysis in combination with phylogenetic analysis, where species with clear WGD events should be selected as reference species, so these plants were selected due to their well-documented background of the WGD events[9]. Additionally, five representative Lamiaceae species other than Isodon rubescens were selected for phylogenetic analysis, and the number of Lamiaceae species accounted for 46.7% of the total number of species selected. The constructed phylogenetic tree correctly reflects the phylogenetic positions of each species and preliminarily reveals the phylogenetic positions of the two forms of Isodon rubescens, meeting the needs of our experiment.
To avoid future confusion, we have revised the manuscript by adding the following sentence in the first paragraph of the phylogenetic and duplication analysis of whole-genome section: "Among these, thalecress, rice, and grape were included due to their well-documented background of the Whole-genome doubling (WGD) events, which can serve as reference species in later WGD analysis."

Q4: Question about chromosome naming.
A4: Thank you for the helpful comment. Sorry for our mistake. As suggested, we have now adjusted the chromosome naming to ensure that the chromosome order in the collinearity map of the two forms of Isodon rubescens is consistent and I have replaced Arabidopsis with Salvia miltiorrhiza, a medicinal model plant from the Lamiaceae family.

Q5: Molecular clock time estimation with insufficient sample quantity, the description is not rigorous.
A5: Thank you for the constructive comment. After consulting the materials, we realize further population genetic experiments would be necessry to solidly prove this conclusion. Therefore, we have removed the description of molecular time in the revised manuscript to avoid misleading the readers.

Q6: The SNP calculation is correct, but the results, relying on the current sample size, are not rigorous enough.
A6: Thank you for the constructive comment. We agree with your comment that SNP analysis based on the small sample size may not be accurate. Since the focus of this paper is the analysis of SV (Structural Variation) and PAV (Presence/Absence Variation), we have removed this part of the description in the revised manuscript to avoid misleading the readers.

Q7: The title of the article mentions the difference in key enzymes of the diterpenoid synthesis pathway, but the main text lacks this data.
A7: Thank you for the helpful comment. We have revised the title to: "High-quality assembly of the T2T genome for Isodon rubescens f. lushanensis reveals genomic structure variations between two forms of Isodon rubescens".

Responding to Reviewer 2
This article provides a detailed description of the T2T genome sequencing results of a variant of Isodon rubescens---Isodon rubescens f. lushanensis. Overall, the sequencing results are reliable, the description is reasonable, and the sequencing quality has greatly improved compared to previous genome of I. rubescens (Hemsl.) Hara. Based on the genomes of the two I. rubescens, the author has conducted bioinformatics analysis such as systematic evolution and structural variation, explaining the phylogenetic status of the two I. rubescens from a molecular perspective and also identifying structural variation genes. It meets the basic requirements of a data analysis article, but there are still a few detailed issues in the article that need to be revised.
A: The authors thank the reviewer for the positive comments.

Q1: The author needs to provide further explanation of this statement and its significance.
The gene fragment sequence comparison results reveal a low proportion of base deletions—72 (0.3%)—in the ITS (internal transcribed spacer) sequences of the two chemotypic variations of I. rubescens. This

finding suggests that the most relevant genes for chemotype formation are those involved in the synthesis and regulation of secondary metabolites.

A1: Thank you for the helpful comment. The statement you mentioned is a direct quote from the original text of the article. To avoid future confusion, we have revised the manuscript by adding the following sentence in the section: “Since the chemical type is divided based on the chemical composition, which is based on the secondary metabolites of plants, the study of the synthetic pathways related to plant secondary metabolites and the functional genes that regulate these synthetic pathways can directly elucidate the mechanism of the formation of chemical types.”

Q2: Clarify whether the "V Chromosome" in Figure 1 represents the collinearity between the two or their respective collinearities.
A2: It demonstrates both the collinearity between the two and the internal collinearity within each one. I have made a revision to the caption in Figure 1.

Q3: Ensure that chemical formulas and gene names, such as ent, Copia, and Gypsy, are italicized.
A3: Checked and revised as suggested.

Q4: Check the writing in the article and figures for proper formatting, such as inserting a space after genus names followed by a period and avoiding italicization of family names.
A4: Checked and revised as suggested.

Q5: Writing issues in the article: Is there an extra 'd' at line 102; an empty line at line 129; the font at lines 583 and 675 needs to be modified to be consistent with other references.
A5: Checked and revised as suggested.

Q6: Typesetting issues in the article: The content under the headings 108-141 needs to be aligned.
A6: Checked and revised as suggested.

Q7: Image quality in the article: Some of the images in the article are not clear, such as Figure 4.
Q7: Checked and revised as suggested.

Reference

[1]Yaru Li. New Taxa of Rabdosia rubescens[J].Journal of Systematics and Evolution, 1986, 24(1):15-16.(in chinese)
[2]Yanghui, Liudi. Current Situation and Prospective on Resource Evaluation and Sustainable Utilization of Rabdosiae Rubescentis Herba[J]. Traditional Chinese Medicine, 2020, 9(6): 506-514. (in chinese)
[3]Sun H, Han Q. The studies on the plant resources, chemical and antitumor constituents of Isodon rubescens [C] Chinese Botanical Society. Abstracts of the 70th Anniversary Meeting of the Chinese Botanical Society (1933-2003). 2003: 2.(in chinese)
[4]Zhang, HaiBo, Du, Xue, Pu, JianXin, Wang, YuanYuan, He, Fei, Zhao, Yong, Li, XiaoNian, Luo, Xiao, Xiao, WeiLie, Li, Yan, Sun, HanDong. Two novel diterpenoids from Isodon rubescens var. lushanensis. TETRAHEDRON LETTERS[J]. 2010, 51(32): 4225-4228,
[5]Zhang, HaiBo, Pu, JianXin, Wang, YuanYuan, He, Fei, Zhao, Yong, Li, XiaoNian, Luo, Xiao, Xiao, WeiLie, Li, Yan, Sun, HanDong. Four New ent-Kauranoids from Isodon rubescens var. lushanensis and Data Reassignment of Dayecrystal B. CHEMICAL & PHARMACEUTICAL BULLETIN[J]. 2010, 58(1): 56-60,
[6]Han Q B , Xiang W , Li R T ,et al.Ent-kaurane diterpenoids from Isodon rubescens var. lushanensis.[J].Chemical & Pharmaceutical Bulletin, 2003, 51(3):269-272..
[7] Chen S, Yin L, Song J, Cui C, Meng C. Molecular analysis of different origin of rabdosia rubescens germplasm resources [J]. Asia-Pacific Traditional Medicine, 2016(16): 5-9.(in chinese)
[8] Lian C, Yang H, Lan J, Zhang X, Zhang F, Yang J, Chen S. Comparative analysis of chloroplast genomes reveals phylogenetic relationships and intraspecific variation in the medicinal plant Isodon rubescens. PLoS One. 2022 Apr 6;17(4):e0266546. doi: 10.1371/journal.pone.0266546. PMID: 35385539; PMCID: PMC8985940.
[9] Guo, L., Winzer, T., Yang, X., Li, Y., Ning, Z., He, Z., Teodor, R., Lu, Y., Bowser, T.A., Graham, I.A., et

al. (2018). The opium poppy genome and morphinan production. Science 362, 343-347.Jiao, Y. (2018). Double the Genome, Double the Fun: Genome Duplications in Angiosperms. Mol Plant 11, 357-358.
