# Supplementary material for: High-quality assembly of the T2T genome for Isodon rubescens f. lushanensis reveals genomic structure variations between 2 typical forms of Isodon rubescens
Source: Gigascience. 2024 Oct 10;13:giae075. doi: 10.1093/gigascience/giae075 (PMC11466039; doi:10.1093/gigascience/giae075)
Supplement: giae075_Reviewer_1_Report_Original_Submission — Henrik Toft Simonsen -- 6/19/2024 [file giae075_reviewer_1_report_original_submission.pdf]

Reviewer Report

Title: High-quality assembly of the T2T genome for Isodon rubescens f. lushanensis reveals genomic structure variations between two typical forms of Isodon rubescens

Version: Original Submission **Date:** 6/19/2024

Reviewer name: Henrik Toft Simonsen

Reviewer Comments to Author:

The authorsFirst thing, the name of the plant. I have search high and low, and I cannot find any formal database listing that recognize the name Isodon rubescens f. lushanensis. All databases I have search, IPNI, Kew Gardens, NCBI etc. declare that the name Isodon rubescens f. lushanensis is a synonym of Isodon rubescens. Please, if you need to publish a genome, then you need to be 120% of the species you are working with. Thus I would like to see the documentation in form of ITS, MatK or similar species ID sequences that this is truly a different variety of all other Isodon. I would recommend the authors, just to rename the species to Isodon rubescens (Hemsl.) H.Hara, following the formal nomenclarture at IPNI or Kew Gardens. If this cannot be established I would recommend a rejection, and this is vital for the whole publication. This is also the name used for the other Isodon rubescens genome. So you difference is merely a different ecotype and NOT a difference species/variety.Secondly, this then leads to the change in the title, so not to typical forms, but more ecotypes. Still interesting, but the terminology have to change.Precisely as you do the paper, you name the plants, I. rubescens Lushan and Jiyuan, so two different ecotypes. Table 1 and even more clearly in figure 2, also clearly show that the differences are way to small to be two different species, if the JY genome had been 100%. By the way, the reference wang 2022 in table 1 is NOT in the ref list, maybe this is actualy [7]. There are many species where there is several ecotype, A. thaliana is properly the best known example.Then you start on comparative genomics. Why do you compare with Arabidopsis, rice and wine (like figure 2b and c)? 1. the genome is way smaller, it is not a lamiaceae plant, and it is not even close to being related to your plant. It is like comparing a zebra fish genome with a human. Why not use the T2T genomes of Scutellaria baicalensis or lavender? At least show that you understand that plants are very very different, the evolution is very old, and that lamiaceae is not the same as brassicaceae. With new genomes coming out every day, there is no need to compare with something really far far away. This also go for your phylogenetic analysis? why? You have 5 lamiaceae genomes, maybe you can use one or two as outgroup, but we already know that this . Stick to lamiaceae, there is enough data there, also for your diterpenoid story. THus, please update all of this part of the paper. In figure 2 you add the JY genome, but you say that it is only 94% complete, thus the representation is not equal and you cannot really determine the split of the two species. You also use different numbers? WHy not say that Chr8 JY is the same a CHr9 of LS - thus should be called Chr8 in LS as well. JY as first and this set the numbers. Again you try to claim too much. Be honest, JY was first, and you base you numbers on this as much as possible. Maybe try to map your assemply to their and see magic. The same JY2, 3, 4 which is LS 4, 5, 6. Just look at figure 2 and see how simple this is? It is not honest to give new numbers to chromosomes.Maybe you can explain what happend for JY 1 and 11, maybe they had a misassembly that in your assembly gave 3 and 8. I really

encourage you to try to use a progressive thought and not just trying to build "new" knowledge, when you can use existing. We see this too much in Bioinformatics, and in the end we have to clean up the mess after several publications. There is only 12 chromosomes, thus, please please assign the to the same numbers. Like was done for Arabidopsis, human, and many other species.Likewise, the goegraphical split is only 200 km, so please rethink these claims on gentic clock splits (figure 3). We can do all kind of calculations, but they have to make sence in real life, and this do not do that. You claim a split at 8.7 mio years, +- 5 mio years??? Ok can you actually do this, with just two samples of such a closely related species? No, the statistical evidence is based on n=2. There is no significance in that. Had you sequenced 10 plants of JY and 10 of LS seperately maybe, but here you show the n=2, so no. So I. rubescens is ONE species, and two ecotypes not seperated in time as you suggest, at least not based on the presented data. Finally your SNP detection hammer in that this is one species. You find 3 mio. Between two humans there is 4-5 mio SNPs (fast methos), in the much smaller Arabidopsis genomes there are also millions of SNPs. So again, it is one species and two ecotypes (https://www.ncbi.nlm.nih.gov/pmc/articles/PMC3290786/ and latter, this is the first one, but latter have confirmed even more SNPs). You calcultaion is correct, but the conclusions are over streching your data.Where is data on the differences in diterpenoid biosynthesis?This is totally missing from the data presentation, so please delete this from the title, since you do NOT show any differences in the diterpenoid biosynthesis. I wonder why you mention this in the title when you do not show it.I was just about to reject the paper, but You get a change to make a major revision, since this is a nice T2T, but please please consider your claims very carefully.

Level of Interest

Please indicate how interesting you found the manuscript: Choose an item.

Quality of Written English

Please indicate the quality of language in the manuscript: Choose an item.

Declaration of Competing Interests

Please complete a declaration of competing interests, considering the following questions:

- Have you in the past five years received reimbursements, fees, funding, or salary from an organisation that may in any way gain or lose financially from the publication of this manuscript, either now or in the future?
- Do you hold any stocks or shares in an organisation that may in any way gain or lose financially from the publication of this manuscript, either now or in the future?
- Do you hold or are you currently applying for any patents relating to the content of the manuscript?
- Have you received reimbursements, fees, funding, or salary from an organization that holds or has applied for patents relating to the content of the manuscript?

- Do you have any other financial competing interests?
- Do you have any non-financial competing interests in relation to this paper?

If you can answer no to all of the above, write 'I declare that I have no competing interests' below. If your reply is yes to any, please give details below.

'I declare that I have no competing interests'

I agree to the open peer review policy of the journal. I understand that my name will be included on my report to the authors and, if the manuscript is accepted for publication, my named report including any attachments I upload will be posted on the website along with the authors' responses. I agree for my report to be made available under an Open Access Creative Commons CC-BY license (http://creativecommons.org/licenses/by/4.0/). I understand that any comments which I do not wish to be included in my named report can be included as confidential comments to the editors, which will not be published.

Choose an item.

To further support our reviewers, we have joined with Publons, where you can gain additional credit to further highlight your hard work (see: https://publons.com/journal/530/gigascience). On publication of this paper, your review will be automatically added to Publons, you can then choose whether or not to claim your Publons credit. I understand this statement.

Yes Choose an item.
