# Supplementary material for: High-quality assembly of the T2T genome for Isodon rubescens f. lushanensis reveals genomic structure variations between 2 typical forms of Isodon rubescens
Source: Gigascience. 2024 Oct 10;13:giae075. doi: 10.1093/gigascience/giae075 (PMC11466039; doi:10.1093/gigascience/giae075)
Supplement: giae075_Reviewer_2_Report_Original_Submission — Wei Sun -- 7/1/2024 [file giae075_reviewer_2_report_original_submission.pdf]

Reviewer Report

Title: High-quality assembly of the T2T genome for Isodon rubescens f. lushanensis reveals genomic structure variations between two typical forms of Isodon rubescens

Version: Original Submission **Date:** 7/1/2024

Reviewer name: Wei Sun

Reviewer Comments to Author:

This article provides a detailed description of the T2T genome sequencing results of a variant of Isodon rubescens---Isodon rubescens f. lushanensis. Overall, the sequencing results are reliable, the description is reasonable, and the sequencing quality has greatly improved compared to previous genome of I. rubescens (Hemsl.) Hara. Based on the genomes of the two I. rubescens, the author has conducted bioinformatics analysis such as systematic evolution and structural variation, explaining the phylogenetic status of the two I. rubescens from a molecular perspective and also identifying structural variation genes. It meets the basic requirements of a data analysis article, but there are still a few detailed issues in the article that need to be revised:The author needs to provide further explanation of this statement and its significance.The gene fragment sequence comparison results reveal a low proportion of base deletionsâ€”72 (0.3%)â€”in the ITS (internal transcribed spacer) sequences of the two chemotypic variations of I. rubescens. This finding suggests that the most relevant genes for chemotype formation are those involved in the synthesis and regulation of secondary metabolites.Additionally, here are some points that the author should consider:1. Clarify whether "Vchromosome" represents collinearity between the two chemotypes or within the genome.2. Ensure that chemical formulas and gene names, such as ent, Copia, and Gypsy, are italicized.3. Check the writing in the article and figures for proper formatting, such as inserting a space after genus names followed by a period and avoiding italicization of family names.4. Writing issues in the article: Is there an extra 'd' at line 102; an empty line at line 129; the font at lines 583 and 675 needs to be modified to be consistent with other references.5. Typesetting issues in the article: The content under the headings 108-141 needs to be aligned.6. Image quality in the article: Some of the images in the article are not clear, such as Figure 4.

Level of Interest

Please indicate how interesting you found the manuscript: Choose an item.

Quality of Written English

Please indicate the quality of language in the manuscript: Choose an item.

Declaration of Competing Interests

Please complete a declaration of competing interests, considering the following questions:

- Have you in the past five years received reimbursements, fees, funding, or salary from an organisation that may in any way gain or lose financially from the publication of this manuscript, either now or in the future?
- Do you hold any stocks or shares in an organisation that may in any way gain or lose financially from the publication of this manuscript, either now or in the future?
- Do you hold or are you currently applying for any patents relating to the content of the manuscript?
- Have you received reimbursements, fees, funding, or salary from an organization that holds or has applied for patents relating to the content of the manuscript?
- Do you have any other financial competing interests?
- Do you have any non-financial competing interests in relation to this paper?

If you can answer no to all of the above, write 'I declare that I have no competing interests' below. If your reply is yes to any, please give details below.

I declare that I have no competing interests

I agree to the open peer review policy of the journal. I understand that my name will be included on my report to the authors and, if the manuscript is accepted for publication, my named report including any attachments I upload will be posted on the website along with the authors' responses. I agree for my report to be made available under an Open Access Creative Commons CC-BY license (http://creativecommons.org/licenses/by/4.0/). I understand that any comments which I do not wish to be included in my named report can be included as confidential comments to the editors, which will not be published.

Choose an item.

To further support our reviewers, we have joined with Publons, where you can gain additional credit to further highlight your hard work (see: https://publons.com/journal/530/gigascience). On publication of this paper, your review will be automatically added to Publons, you can then choose whether or not to claim your Publons credit. I understand this statement.

Yes Choose an item.
